# Nuclear mitochondrial acetyl-CoA acetyltransferase 1 orchestrates natural killer cell-dependent antitumor immunity in colorectal cancer

**DOI:** 10.1038/s41392-025-02221-y

**Published:** 2025-04-28

**Authors:** Chen Wei, Kun Liao, Hao-Jie Chen, Zi-Xuan Xiao, Qi Meng, Ze-Kun Liu, Yun-Xin Lu, Hui Sheng, Hai-Yu Mo, Qi-Nian Wu, Yi Han, Zhao-Lei Zeng, Xin-Yuan Guan, Hui-Yan Luo, Huai-Qiang Ju, Rui-Hua Xu

**Affiliations:** 1 Department of Medical Oncology, Sun Yat-sen University Cancer Center, State Key Laboratory of Oncology in South China, Guangdong Provincial Clinical Research Center for Cancer, Sun Yat-sen University, Guangzhou, PR China; 2 Research Unit of Precision Diagnosis and Treatment for Gastrointestinal Cancer, Chinese Academy of Medical Sciences, Guangzhou, PR China; 3 Department of Clinical Oncology, Shenzhen Key Laboratory for Cancer Metastasis and Personalized Therapy, The University of Hong Kong-Shenzhen Hospital, Shenzhen, PR China

**Keywords:** Cancer metabolism, Gastrointestinal cancer, Tumour immunology, Cancer microenvironment

## Abstract

Tumor metabolism often interferes with the immune microenvironment. Although natural killer (NK) cells play pivotal roles in antitumor immunity, the connection between NK cells and tumor metabolism remains unclear. Our systematic analysis of multiomics data and survival data from colorectal cancer (CRC) patients uncovered a novel association between mitochondrial acetyl-CoA acetyltransferase 1 (ACAT1) and NK cell infiltration that influences disease progression. ACAT1, a metabolic enzyme involved in reversible conversion of acetoacetyl-CoA to two molecules of acetyl-CoA, exhibits nuclear protein acetylation activity through its translocation. Under immune stimulation, mitochondrial ACAT1 can be phosphorylated at serine 60 (S60) and enters the nucleus; however, this process is hindered in nutrient-poor tumor microenvironments. Nuclear ACAT1 directly acetylates lysine 146 of p50 (NFKB1), attenuating its DNA binding and transcriptional repression activity and thereby increasing the expression of immune-related factors, which in turn promotes NK cell recruitment and activation to suppress colorectal cancer growth. Furthermore, significant associations are found among low nuclear ACAT1 levels, decreased S60 phosphorylation, and reduced NK cell infiltration, as well as poor prognosis in CRC. Our findings reveal an unexpected function of ACAT1 as a nuclear acetyltransferase and elucidate its role in NK cell-dependent antitumor immunity through p50 acetylation.

## Introduction

Colorectal cancer (CRC) has been the third most common cancer globally and is considered as a poorly immunogenic tumor. 85% of CRC patients display microsatellite-stable (MSS) phenotype manifested by limited expression of neoantigens, impaired CD8^+^ T cell recognition and scant infiltration of immune cells,^1^ thus leading to poor response to immunotherapy. Consequently, how to improve the CRC immune microenvironment is urgently needed.

Natural killer (NK) cells are called as “professional killers” for its excellent capacity in recognition and killing, thus always playing vital roles in innate immune system. Many studies have demonstrated that tumor-infiltrating NK cells have a positive effect on CRC prognosis.^2–4^ In contrast to T and B lymphocytes, the recognition and activation of NK cells depend on the integrated signals arising from germline-encoded activating and inhibitory receptors.^5,6^ Regarding activating receptors, KIR2DS, which belongs to the killer cell Ig-like receptor (KIR) family, can recognize HLA class I histocompatibility antigen, C alpha chain (HLA-C), and lead to the activation of NK cells.^7^ In addition, the lectin-like type 2 transmembrane receptor NKG2D, expressed on the majority of NK cells, recognizes UL16-binding proteins (ULBP1/ULBP2) and MHC class I-chain-related proteins (MICA/MICB) to promote NK cell activation and cytotoxicity^8^; other receptors include the natural cytotoxicity receptors (NCRs) NKp30 (NCR3/CD337), NKp44 (NCR2/CD336), and NKp46 (NCR1/CD335).^9–11^ Moreover, accumulated studies have confirmed that NK cells exert outstanding roles in antitumor immunity^12–14^ and participate in many processes related to tumor control, including direct killing and proinflammatory cytokine secretion.^15,16^ However, NK cells are plastic and heterogeneous because they can display different phenotypes when exposed to the complex tumor microenvironment (TME).^17,18^ Dysfunction of NK cells in TME remains a major obstacle for cancer immunotherapies against solid tumors.

Cancer metabolism has been extensively accepted to influence antitumor immunity. The functions of immune cells in the TME can be affected by multiple factors associated with aberrantly regulated metabolic enzymes in tumor cells, such as intermediates generated during metabolic reactions, cellular signaling events modulated by metabolites, and alterations in nutrient uptake and utilization.^19–23^ However, little is known about the influence of tumor metabolism on NK cells. Therefore, exploring the mechanism by which tumor metabolism impacts NK cells may provide novel insights for the development of approaches to overcome CRC.

Mitochondrial acetyl-CoA acetyltransferase (ACAT1), the first purified thiolase located in the mitochondrial matrix,^24,25^ functions in both synthetic and degradative pathways to catalyze the Claisen condensation of two molecules of acetyl-CoA into acetoacetyl-CoA or the reverse reaction of acetoacetyl-CoA into two molecules of acetyl-CoA, thus participating in isoleucine degradation, ketogenesis, ketolysis, and fatty acid oxidation.^26,27^ Therefore, ACAT1 deficiency often leads to an autosomal recessive inherited metabolic disorder. ACAT1, though potentially functioning as a mitochondrial acetyltransferase, primarily targets specific mitochondrial substrates, such as acetyl-PDP1, PDHA1, and GNPAT.^28,29^ However, the understanding of its roles in extramitochondrial compartments (including the nucleus) and the broader implications of its role as an acetyltransferase are currently limited. The exact mechanisms by which ACAT1 contributes to antitumor immunity remain to be fully elucidated.

In this study, we highlight the role of nuclear ACAT1 in NK cell infiltration and activation. We demonstrate that mechanistically, metabolic enzyme ACAT1 located in mitochondrion could response to complicated signals and translocate to nucleus. Nuclear ACAT1, functioned as acetyltransferase, directly acetylates p50 at lysine 146 (K146), impairing the DNA binding and transcriptional repression activity of p50. Consequently, the expression of immune-related factors that can recruit and activate NK cells is inspired to prevent CRC growth.

## Results

### ACAT1 promotes cytotoxic NK cell infiltration to suppress CRC growth

**Fig. 1:**
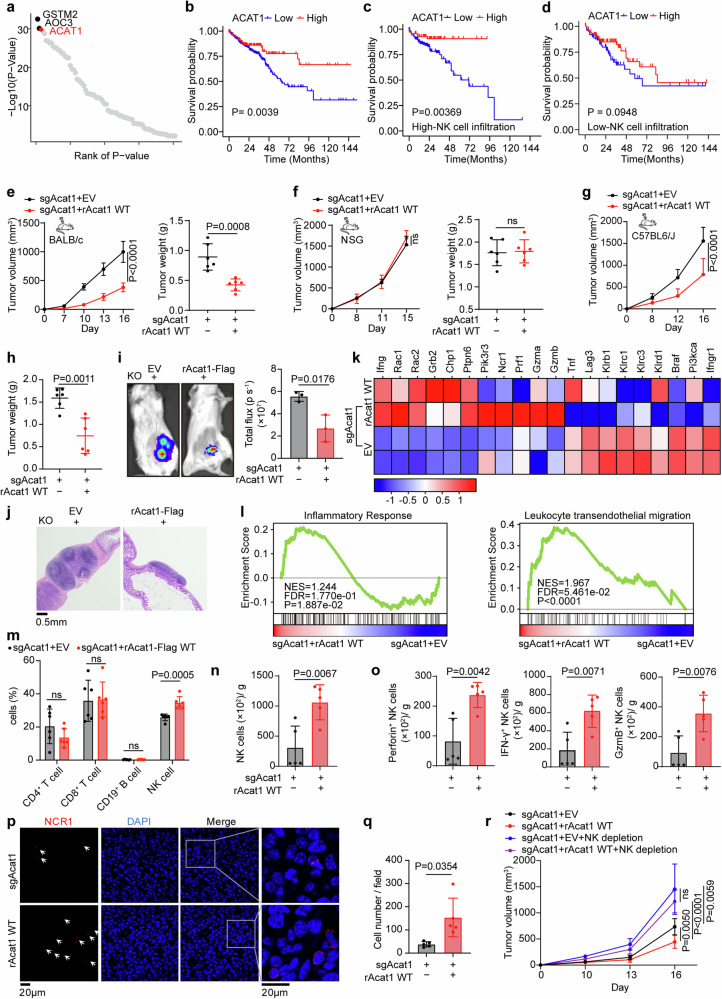
ACAT1 promotes cytotoxic NK cell infiltration to suppress CRC growth. **a** Top-ranked proteins positively correlated with NK cell infiltration in CRC. The processes were as follows: first, metabolic enzymes whose gene expression levels were significantly correlated with CRC patient survival (OS, DFS) in TCGA-COADREAD cohort were identified (p_Chisq < 0.01; Supplementary Table 1); next, NK cell infiltration in each sample was estimated by MCPcounter on transcriptomic data from TCGA-COADREAD cohort and samples were divided into high and low NK cell infiltration groups based on the median estimated NK cell infiltration levels, after this, genes shown significant prognostic associations exclusively in the high NK cell infiltration group were selected (p_Chisq < 0.01; Supplementary Table 2); finally, genes/proteins expression analysis was conducted using transcriptomic (TCGA-COADREAD) (pvalue.wilcox < 0.001; Supplementary Table 3) and proteomic (CPTAC-COAD) (p < 0.01; Supplementary Table 4) datasets (referred to Suhas and colleagues^49^ study), and only differentially expressed proteins between normal and CRC tissues were selected. The final candidates were ranked based on p-values obtained from the proteomic differential analysis (p < 0.01; Supplementary Table 5). Kaplan-Meier survival analysis of ACAT1 expression for overall survival (**b**) or based on ACAT1 expression for patients with high-NK cell infiltration or low-NK cell infiltration (**c**, **d**) (as determined via MCPcounter) in the TCGA COADREAD cohort. Acat1-KO CT26 cells rescued with Acat1-Flag WT or empty vector (EV) were subcutaneously injected into BALB/c mice (**e**, 6 mice per group) and NSG mice (**f**, 6 mice per group). Tumor growth (left) and tumor weight (right) were measured. **g**, **h** Acat1-KO B16F10 cells rescued with Acat1-Flag WT or EV were subcutaneously injected into C57BL/6J mice (6 mice per group). Tumor growth (**g**) and tumor weight (**h**) were measured. **i** Luciferase-expressing Acat1-KO CT26 cells rescued with Acat1-Flag WT or EV were injected into the cecum of BALB/c mice (3 mice per group). Bioluminescence imaging was performed 14 days after injection, and representative images of tumor growth are shown (left). Bioluminescent quantification was calculated (right). **j** Representative H&E staining of tumors from mice in **i**. **k** NK cells were isolated from tumors 12 days after orthotopic injection and subjected to Smart-seq2. Heatmap of the mRNA expression levels of the genes related to NK cell cytotoxicity was shown. **l** GSEA of inflammatory response and leukocyte transendothelial migration signaling genes based on Smart-seq2 data. **m** Acat1-KO CT26 cells rescued with Acat1-Flag WT or EV were subcutaneously injected into BALB/c mice (up, 6 mice per group), and the percentages of tumor-infiltrating CD4^+^ T cells, CD8^+^ T cells, CD19^+^ B cells and NK (NKp46^+^) cells were calculated. The absolute numbers of NK cells (**n**), Perforin^+^, IFN-γ^+^, GzmB^+^ NK cells (**o**) in tumors from BALB/c mice subcutaneously injected with Acat1-KO CT26 cells re-expressing Acat1-Flag WT or not (5 mice per group). **p** Immunofluorescence staining with an anti-NCR1 (anti**-**NKp46 (red)) antibody and DAPI (blue) was performed in tumor tissues harvested from BALB/c mice treated as described in **n**, **o**. Representative images of IF staining are shown (**p**); DAPI, 4′,6-diamidino-2-phenylindole. **q** NKp46-positive cell numbers per field based on **p** were calculated by ImageJ. **r** BALB/c mice with subcutaneous CT26 tumor model (6 mice per group) were treated with rabbit serum or anti-ASGM1antibody, and tumor growth was measured. The data are shown as the means ± SDs. Two-tailed log**-**rank test (**b**–**d**), unpaired two-tailed t test (**e**, **f** (tumor weight), **h**, **i**, **m**–**o**), Welch’s t test (**q**) or two-way ANOVA (**e**–**g**, **r** (tumor volume))

NK cells play important roles in the elimination of early tumors,^30,31^ and we also observed that tumor-infiltrating NK cells were enriched mainly in patients with early-stage colorectal cancer in our own CRC cohort (Supplementary Fig. 1a). Considering the pivotal correlation between tumor metabolism and NK cell immunity, we first investigated the metabolic proteins that play positive roles in CRC-infiltrating NK cells via systematic analysis of multiomics data and survival data from CRC patients (Supplementary Fig. 1b, Supplementary Tables 1–5). The three top-ranked proteins are shown (Fig. 1a). The immune-related functions of the two top-ranked metabolic enzymes—glutathione S-transferase mu 2 (GSTM2) and amine oxidase, copper containing-3 (AOC3)—have been widely discussed, indicating the reliability of our analytical method.^32–35^ However, the effect of the metabolic enzyme ACAT1 on the immune microenvironment in CRC remains unknown. Next, we validated the positive correlation between the protein level of ACAT1 and the abundance of tumor-infiltrating NK cells in patients with stage 1–2 colorectal cancer (Supplementary Fig. 1b). Notably, ACAT1 expression was positively associated with overall survival, especially when the abundance of tumor-infiltrating NK cells was high (Fig. 1b–d), suggesting that ACAT1 is beneficial for NK cell immunity in CRC.

To confirm the novel capacity of ACAT1 in antitumor immunity, we knocked out endogenous Acat1 in CT26 (MSS-CRC) cells and restored Acat1 expression with Acat1-Flag (Supplementary Fig. 1d, e) to examine tumor growth in BALB/c mice and NOD-scid IL2Rg^null^ (NSG) mice. Strikingly, Acat1 suppressed tumor growth in immunocompetent mice but not in immunodeficient mice (Fig. 1e, f); additionally, cells whose expression level of rAcat1 was close to wild type cells grew at the same rate of wild type cells (Supplementary Fig. 1g). These results indicate that tumor inhibitory functions of ACAT1 relies on an intact immune system. Besides, in another immunologically cold tumor model, Acat1 suppressed melanoma tumor growth (Fig. 1g, h), revalidating its antitumor function. Furthermore, orthotopic models also revealed that Acat1 impaired colorectal tumor growth (Fig. 1i, j and Supplementary Fig. 1h).

Given the heterogeneous phenotypes of NK cells in the TME, we isolated tumor-infiltrating NK cells from mice in the orthotopic CT26 tumor model and performed Smart-seq2 to ascertain whether the NK cells recruited into tumors are activated or dysfunctional. Intriguingly, Acat1-rescued tumors upregulated the mRNA expression of genes involved in NK cell-mediated cytotoxicity (IFNG, RAC1, RAC2) with a parallel decrease of key inhibitory receptors (LAG3, KLRB1 and KLRC1) on NK cells (Fig. 1k, Supplementary Table 6). Furthermore, gene set enrichment analysis (GSEA) suggested that ACAT1 increased inflammatory response, leukocyte transendothelial migration, and oxidative phosphorylation signals in NK cells (Fig. 1l and Supplementary Fig. 1i), which are crucial for the function and migration of NK cells. These results suggest that ACAT1 significantly promotes the cytotoxic effects of tumor-infiltrating NK cells and facilitates NK cell recruitment.

We further confirmed the effects of ACAT1 on NK cell-dependent antitumor immunity by flow cytometric analysis and found that the percentage of total NK cells but not that of CD4^+^ T cells, CD8^+^ T cells, or CD19^+^ B cells was increased in the Acat1-rescued group (Fig. 1m and Supplementary Fig. 1j, k). Moreover, the Acat1-rescued group presented greater proportions and absolute numbers of NK cells and cytotoxic NK cells (Fig. 1n, o and Supplementary Fig. 1l, m), consistent with our Smart-seq2 data. Intuitively, we also observed greatly increased accumulation of NK cells in Acat1-rescued tumors (Fig. 1p, q). Of note, the tumor growth of mice treated with anti-asialo-GM1 antibody demonstrated that the antitumor effect of ACAT1 was dependent on NK cells (Fig. 1r and Supplementary Fig. 1n, o). In conclusion, we confirmed that ACAT1 improved activated NK cells infiltration to restrict tumor growth.

### Nuclear ACAT1 engages activated NK cells to accumulate in the TME

**Fig. 2:**
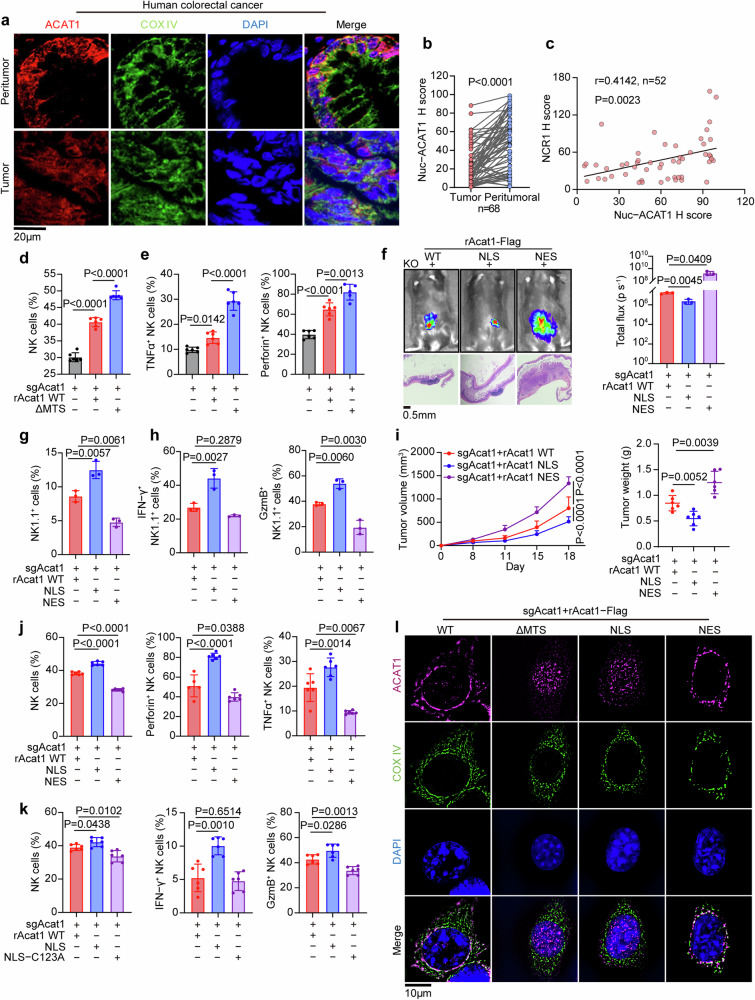
Nuclear ACAT1 engages activated NK cells to accumulate in the TME. **a**, **b** Immunofluorescence staining with anti-ACAT1 and anti-COX IV (mitochondrial marker) antibodies was performed in tumor tissues and peritumoral tissues from patients with colorectal cancer. Representative images of IF staining are shown (**a**). Semiquantitative scores of nuclear ACAT1 staining in tumor and peritumoral tissues were determined via HALO (**b**). Nuc, nuclear. **c** Semiquantitative scoring of IF and IHC staining was carried out with HALO, and the correlation between the nuclear ACAT1 (IF) and NCR1 (IHC) levels was analyzed. **d**, **e** Acat1-KO CT26 cells rescued with Acat1-Flag WT or ΔMTS were subcutaneously injected into BALB/c mice (6 mice per group), and tumors were excised 13 days after injection for flow cytometric analysis to calculate the percentage of tumor-infiltrating NK cells (**d**) and evaluate the expression of TNFα and Perforin in NK cells (**e**). **f**–**h** Acat1-KO MC38 cells rescued with Acat1-Flag WT, NLS, or NES were infected with lentivirus expressing luciferase and injected into the cecum of C57BL/6J mice. Bioluminescence imaging was performed 18 days after injection. Representative images of tumor growth and HE staining are shown (**f**, left); Bioluminescent quantification was calculated (3 mice per group) (**f**, right). Tumors were excised 13 days after injection for flow cytometric analysis to calculate the percentages of tumor-infiltrating NK1.1^+^ cells (**g**) and of IFN-γ^+^ and GzmB^+^ NK1.1^+^ cells (3 mice per group) (**h**). **i**, **j** Acat1-KO CT26 cells rescued with Acat1-Flag WT, NLS or NES were subcutaneously injected into BALB/c mice. Tumor growth and tumor volume were measured (6 mice per group) (**i**). Flow cytometric analysis was performed 13 days after injection to calculate the percentages of tumor-infiltrating NK cells, TNFα^+^ NK cells, and Perforin^+^ NK cells (6 mice per group) (**j**). **k** Acat1-KO CT26 cells rescued with Acat1-Flag WT, NLS or NLS-C123A were subcutaneously injected into BALB/c mice (6 mice per group). Flow cytometric analysis was performed 13 days after injection to calculate the percentages of tumor-infiltrating NK cells, IFN-γ^+^ NK cells, and GzmB^+^ NK cells. **l** Representative images of immunofluorescence staining for rACAT1-Flag WT and the indicated mutants in CT26 cells with anti-Flag, and anti-COX IV antibodies. Images were reconstructed by SIM (Structured Illumination Microscopy). COX IV, mitochondrial marker. The data are shown as the means ± SDs. Paired two-tailed t test (**b**), Pearson correlation test (**c**), one-way ANOVA (**d**, **e**, **f**, **g**–**k** (**i**, tumor weight)), or two-way ANOVA (**i**, tumor volume)

To date, studies on ACAT1 have focused mainly on its mitochondrial function.^28,29,36,37^ Intriguingly, we observed that ACAT1 was located not only in mitochondrion but also in the nucleus (Fig. 2a, Supplementary Fig. 2a) and the level of nuclear ACAT1 was noticeably lower in tumor tissues than in the paired peritumoral tissues (Fig. 2b). Moreover, the level of nuclear ACAT1 was positively correlated with NK cell infiltration in tumor tissues from CRC patients (Fig. 2c), implying an advantage of nuclear ACAT1 in NK cells recruitment. To validate this preliminary hypothesis, we generated Acat1-KO cells rescued with Acat1-Flag WT or Acat1-Flag tagged with a nuclear localization signal (NLS). By subcutaneous tumor models with MC38 or CT26 cells, we found that the NLS group presented the lowest tumor volume and tumor weight among the three groups (Supplementary Fig. 2b, c), and the proportions or absolute numbers of NK cells and cytotoxic NK cells were elevated in NLS group versus WT group (Supplementary Fig. 2d–f). These findings indicate that nuclear ACAT1 supports the accumulation of activated NK cells in the TME.

However, several studies have suggested the tumor-associated functions of ACAT1 in mitochondria.^29,38,39^ To confirm whether nuclear or mitochondrial ACAT1 can impact tumor growth and benefit NK cell-dependent immunity, we constructed a mitochondrial targeting sequence-deleted (ΔMTS) mutant of ACAT1. Astonishingly, tumor growth was appreciably decreased and the proportions of tumor-infiltrating NK cells as well as cytotoxic NK cells were dramatically increased after disruption of the mitochondrial location of ACAT1 (Fig. 2d, e and Supplementary Fig. 2g, h). Correspondingly, ACAT1 ΔMTS was preferentially localized to the nucleus (Fig. 2l and Supplementary Fig. 3a, g), suggesting that the antitumor immune function of ACAT1 is associated with its nuclear location. To exclude the role of mitochondrial ACAT1, we generated Acat1-KO MC38 cells rescued with Acat1-Flag WT, Acat1-Flag NLS, or Acat1-Flag tagged with a nuclear export signal (NES). Before orthotopic injection, we confirmed the ACAT1 NES mutant was no longer located in the nucleus and that the mitochondrial level of the NES mutant was the same as that of Acat1-Flag WT (Supplementary Fig. 2i). Consequently, bioluminescence images and hematoxylin and eosin (H&E) staining of tumor tissues indicated that despite the identical mitochondrial level of ACAT1 in the WT and NES groups, the antitumor capacity was deprived in NES group; moreover, tumor growth was impaired in NLS group (Fig. 2f and Supplementary Fig. 2j). In addition, the proportions of tumor-infiltrating NK cells and cytotoxic NK cells were increased in NLS group but decreased in NES group compared with the WT group (Fig. 2g, h). Furthermore, we also subcutaneously injected CT26 KO cells rescued with Acat1-Flag WT, NLS, or NES into BALB/c mice, and same quality control was conducted as mentioned above (Supplementary Fig. 2i). Notably, comparable results were obtained in tumor growth (Fig. 2i) and flow cytometric analysis of NK cells (Fig. 2j). Moreover, once we depleted NK cells in mice, there was no difference in growth rates among WT, NLS, NES groups (Supplementary Fig. 2k, l). These results further demonstrate that nuclear ACAT1 rather than mitochondrial ACAT1 promotes the recruitment of cytotoxic NK cells to suppress CRC development.

Next, we wondered whether the antitumor function of ACAT1 in the nucleus depends on its enzymatic activity. C126 (C123 in mice) is one of the four key catalytic residues in ACAT1, and is acetylated in the first step of enzyme reation.^26^ Therefore, we constructed the human Flag-ACAT1 C126A mutant and verified that its enzymatic activity was frustrated compared to Flag-ACAT1 WT via in vitro activity assay (Supplementary Fig. 2m). Surprisingly, the antitumor capacity in terms of tumor growth and NK cell infiltration was lost in NLS-C123A group (Fig. 2k and Supplementary Fig. 2n), indicating that the function of nuclear ACAT1 still requires its enzymatic activity. It was noted that the mitochondrial levels of Acat1-Flag NLS-C123A and Acat1-Flag NLS were identical (Supplementary Fig. 3c), but the antitumor effects of these two mutants were clearly different, also reflecting that nuclear but not mitochondrial ACAT1 facilitates cytotoxic NK cell recruitment and impairs tumor growth. The ACAT1 mutants used above were tested to confirm their transfection efficiency and subcellular location by immunoblotting (IB) and IF staining, respectively (Fig. 2l, Supplementary Fig. 3a–g). Besides, no matter in NSG mice or in-vitro MTS assay, tumor cells grew similarly among rAcat1 WT, NLS, NES, and ΔMTS groups (Supplementary Fig. 3h–j), demonstrating that these mutations could not affect cell-intrinsic functions of tumor cells. Collectively, these results demonstrated that nuclear ACAT1 impaired CRC development by inducing the accumulation of activated NK cells in the TME.

### Nuclear ACAT1 directly acetylates NFκB subunit p50 at K146

To investigate the mechanism through which nuclear ACAT1 regulates tumor-infiltrating NK cells. We first speculated that nuclear ACAT1 might affect the level of nuclear acetyl-CoA considering its enzymatic activity. As expected, ACAT1 nuclear translocation resulted in an increased level of nuclear acetyl-CoA (Supplementary Fig. 4a, b and Supplementary Table 7). Acetyl-CoA is a well-known substrate for lysine acetylation; we thus wondered whether the additional nuclear acetyl-CoA generated via nuclear ACAT1 functioned as a general substrate for nuclear protein acetylation. Considering that histone acetylation is highly sensitive to acetyl-CoA availability,^40,41^ we assumed that global histone H2B, H3, and H4 acetylation might be affected. However, we failed to detect obvious differences in the histone acetylation among cells expressing ACAT1-Flag WT, or NLS (Supplementary Fig. 4c). Since previous studies discovered that mitochondrial ACAT1 functioned as acetyltransferase,^28,29,36^ we further pondered that nuclear ACAT1 might also act as an acetyltransferase.

**Fig. 3:**
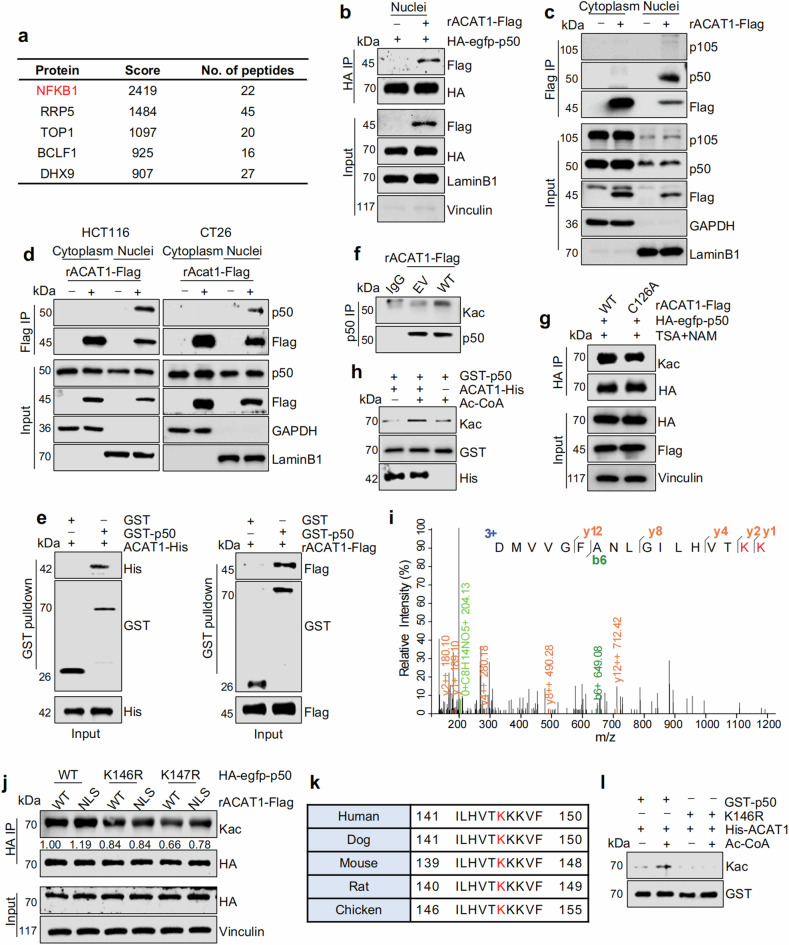
Nuclear ACAT1 directly acetylates p50 at K146 **a** Mass spectrometry (MS) analysis to explore different ACAT1-binding proteins in nucleus of HCT116 cells. The representative candidates are listed. Nuclear factor NF-kappa-B p105 subunit (NFKB1) was identified. **b** HA-egfp-p50 (1-366aa) was immunoprecipitated from HEK293T cells to detect ACAT1-Flag bound to HA-egfp-p50 (1-366aa). **c** RKO cells stably expressing EV or ACAT1-Flag were subjected to subcellular fractionation assay for a subsequent immunoprecipitation (IP) analysis. **d** HCT116 cells and CT26 cells stably expressing EV or ACAT1-Flag/Acat1-Flag were subjected to subcellular fractionation assay for a subsequent IP analysis. **e** Detection of purified ACAT1-His (left) or ACAT1-Flag (right) bound to GST or GST-p50 (1-366aa) via a GST pulldown assay. **f** Endogenous IP with anti-p50 or anti-IgG antibodies was performed to determine the level of acetylated p50 in HEK293T cells transfected with EV or ACAT1-Flag. **g** HA-egfp-p50 (1-366aa) was immunoprecipitated from HCT116 cells co-transfected with ACAT1-Flag WT or C126A and HA-egfp-p50 (1-366aa). Immunoblot analysis was performed with the indicated antibodies. **h** The acetylation level of purified GST-p50 (1-366aa) was determined by incubation with or without purified ACAT1-His in the presence or absence of 10 µM acetyl-CoA (Ac-CoA) in vitro. **i** Subcellular fractionation assay and immunoprecipitation were performed to pull down nuclear HA-egfp-p50 (1-366aa) from HCT116 cells transfected with ACAT1-Flag NLS. K146 and K147 were found to be acetylated in the nucleus via mass spectrometry analysis. **j** IP was performed with anti-HA magnetic beads in HEK293T cells co-transfected with HA-egfp-p50 (1-366aa) WT/K146R/K147R and ACAT1-Flag WT or NLS. Immunoblot analysis was performed with the indicated antibodies. **k** Sequences of the acetylated peptides in the indicated species. **l** Purified GST-p50 (1-366aa) or K146R and purified ACAT1-His were incubated with or without 10 µM Ac-CoA in vitro. Immunoblot analysis was performed with the indicated antibodies. Immunoblots representative of three independent experiments are shown

Then, we immunoprecipitated nuclear ACAT1 to analyze its interacting proteins via mass spectrometry and observed that nuclear factor-kappa B 1 (NFKB1) was the top-ranked protein (Fig. 3a and Supplementary Table 8). NFKB1 is a well-recognized subunit of the NF-κB family and includes two types of protein forms, p105 and p50. The precursor p105 is cytoplasmic, and the mature product p50 tends to translocate to the nucleus, where it dimerizes and binds to DNA.^42,43^ Subsequently, we verified that ACAT1 interacted with p50 but not p105 (Fig. 3b, c and Supplementary Fig. 4d) and this interaction only existed in the nucleus (Fig. 3d). In addition, the direct interaction between ACAT1 and p50 was also confirmed by GST pulldown assay (Fig. 3e). Hence, we wondered whether ACAT1 could acetylate p50. The acetylation levels of endogenous and exogenous p50 were elevated after ACAT1-Flag overexpression (Fig. 3f and Supplementary Fig. 4e), but decreased after the enzymatic activity of ACAT1 was impaired (Fig. 3g). Additionally, when we pretreated cells with trichostatin A (TSA; an inhibitor of class I/II HDACs) and nicotinamide (NAM; an inhibitor of sirtuins), the level of acetylated p50 noticeably increased in cells expressing ACAT1-Flag NLS than in those expressing WT (Supplementary Fig. 4f). Moreover, nuclear but not cytoplasmic p50 was acetylated after ACAT1-Flag overexpression (Supplementary Fig. 4g), suggesting that ACAT1-mediated acetylation of p50 occurs in the nucleus. Importantly, in-vitro acetylation assay showed the acetylation level of purified p50 only increased when incubated with both ACAT1 and acetyl-CoA, confirming that ACAT1 exhibits direct acetyltransferase activity toward p50 (Fig. 3h).

To identify the acetylated lysine (K) residues in p50, mass spectrometry analysis of nuclear HA-p50 immunoprecipitated from HCT116 cells overexpressing ACAT1-Flag NLS was performed, revealing that K146 and K147 were possible acetylated sites in nuclear p50 (Fig. 3i). We further mutated these two residues individually to nonacetylatable arginine (R) residues and compared the acetylation level of each mutant between the ACAT1-Flag NLS and WT expressing cells. Clearly, K146R but not K147R mutation of p50 abrogated NLS-induced acetylation (Fig. 3j). Noteworthily, K146 in p50 is evolutionarily conserved among mammals and chickens (Fig. 3k). Furthermore, the results of in-vitro acetylation assay using purified ACAT1-His incubated with purified GST-p50 or GST-p50 K146R proved that the K146R mutant could no longer be acetylated by ACAT1-His (Fig. 3l). Taken together, these findings demonstrated that nuclear ACAT1 directly acetylated p50 at K146.

### p50 K146 acetylation weakens DNA binding ability and thus promotes NK cell activation and infiltration

**Fig. 4:**
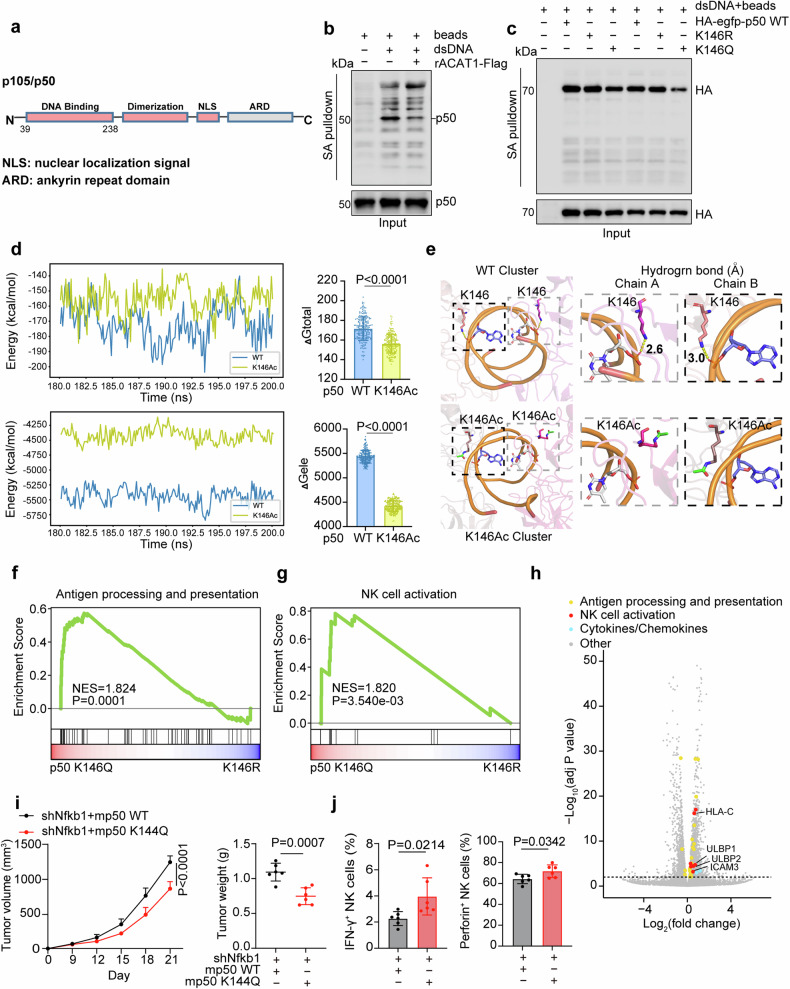
p50 K146 acetylation weakens DNA binding ability and thus promotes NK cell activation and infiltration. **a**, Schematic representation of p105/p50. **b**, **c** Subcellular fractionation assay and DNA pulldown assay were performed to detect endogenous nuclear p50 or exogenous HA-egfp-p50 (1-366aa) WT/K146R/K146Q bound to biotinylated dsDNA (5 µg) conjugated to streptavidin magnetic beads in HCT116 cells transfected with EV or ACAT1-Flag (**b**) and in HEK293T cells (**c**). **d**, **e** Molecular dynamics simulations of p50 homodimers bound to DNA. The total binding free energy (Gtotal, top) and electrostatic energy (Gele, bottom) were compared between p50 WT and K146ac (paired t test) (**d**). The potential hydrogen bonds (colored yellow) in WT-Cluster (top), and K146Ac-Cluster (bottom) are shown. p50 homodimers in WT-Cluster and K146Ac-Cluster are shown as cartoons, DNA is represented by an orange double helix, and the nucleotides bound to p50 are shown as sticks. K146 of p50 homodimer is colored magenta (chain A) or pink (chain B) in WT-Cluster and shown as sticks. K146Ac is colored in hot pink (chain A) or brown (chain B) in K146Ac-Cluster and shown as sticks, with the acetyl group labeled as green sticks (**e**). GSEA of antigen processing and presentation genes (**f**) and NK cell activation genes (**g**) based on RNA-seq data from NFKB1-depleted HCT116 cells rescued with HA-egfp-p50 K146Q/K146R. **h** Volcano plots showing the log_2_-fold changes in gene expression and adjusted P values between HA-egfp-p50 K146Q versus K146R in NFKB1-depleted HCT116 cells. **i**, **j** Nfkb1-depleted CT26 cells rescued with mp50-HA (1-364aa) WT or K144Q were subcutaneously injected into BALB/c mice (6 mice per group). Tumor growth and tumor weight were measured (**i**). Tumors were excised 13 days after injection for flow cytometric analysis to calculate the percentage of tumor-infiltrating cytotoxic NK cells (**j**). Immunoblots representative of three independent experiments are shown. The data are shown as the means ± SDs. Paired t test (**d**), unpaired two-tailed t test (**i** (tumor weight), **j**), or two-way ANOVA (**i** (tumor volume))

The p50 subunit lacks a transactivation domain (TAD) but possesses a DNA-binding domain (Fig. 4a), which facilitates the localization of other transcription factors or coregulators to target genes. However, whether p50 activates or represses transcription depends on the partner p50 assembled with. For instance, p50:p65 heterodimers always stimulate gene expression, but p50:p50 homodimers are generally thought to inhibit gene expression as a result of a basal chromatin binding without TAD or histone deacetylase 1 (HDAC1) recruitment.^38,39,43,44^

Encouragingly, K146 is just located in the DNA-binding domain of p50. Moreover, we found ACAT1 had no effect on upstream signal activation or cellular distribution of p50 (Supplementary Fig. 4h, i). These points implied that the ability of p50 binding to DNA might be impacted after its acetylation by ACAT1. Therefore, DNA pulldown assay was performed and the abundance of DNA-bound p50 decreased obviously in HEK293T cells overexpressing ACAT1-Flag (Fig. 4b). In addition, the DNA-binding ability of HA-egfp-p50 K146Q, a lysine-to-glutamine acetyl-mimetic mutant, was lower than that of the WT or K146R (Fig. 4c). However, there was no significant difference in DNA-binding ability between HA-egfp-p50 WT and K146R (Fig. 4c), potentially because of the low acetylation level of nuclear HA-egfp-p50 WT in vivo. Next, we performed molecular dynamics simulations of p50 homodimers bound to DNA. We added acetyl group to p50 K146 to mimic the natural state of acetylation and also changed p50 K146 to Q146 to ensure consistency with our experimental methods. Specifically, we constructed three protein‒DNA complex structures—WT-Dimer, K146Ac-Dimer, and K146Q-Dimer, and performed structural clustering analysis of these complexes to obtain average structures, namely, WT-Cluster, K146Ac-Cluster, and K146Q-Cluster, respectively. The overlapping structures of the Dimers and Clusters were visualized, and the values of the root mean square deviation (RMSD), a common index for measuring the similarity of two conformations, were 2.133 Å (WT), 2.921 Å (K146Ac), and 2.842 Å (K146Q) (Supplementary Fig. 5a–c). This finding indicated the structures of the Clusters and Dimers were different in the mutation of K146Ac and K146Q. Interestingly, the total binding free energy (Gtotal) and electrostatic energy (Gele) of p50 K146Ac were clearly lower than those of p50 WT (Fig. 4d), and the same trends were observed for K146Q compared with WT (Supplementary Fig. 5d). In fact, hydrogen bonds could no longer be formed between p50 K146 and DNA on either protein chain of K146Ac-Cluster (Fig. 4e). Similarly, Q146 on neither chain of K146Q-Cluster could bind DNA because the distance of hydrogen bonds was over 2.8 Å (Supplementary Fig. 5e). Altogether, these results demonstrate that p50 K146 acetylation decreases the DNA binding capacity of the p50 homodimer.

Considering that p50 homodimers always inhibit gene transcription, we sought to identify the genes potentially upregulated by p50 K146 acetylation. By RNA-seq analysis, we found that the K146Q mutation in p50 altered the expression of a substantial number of genes (2396 upregulated and 1863 downregulated genes). Furthermore, GSEA suggested that antigen processing and presentation and NK cell activation were the main processes upregulated in NFKB1-depleted HCT116 cells rescued with HA-egfp-p50 K146Q (Fig. 4f, g, Supplementary Fig. 5j and Supplementary Table 9, 10). Volcano plots were generated to visualize the changes in the expression of individual genes within these pathways and chemokines or cytokines related to NK cell infiltration (Fig. 4h). We then examined the genes related to NK cell activation and found that the expression of HLA-C, ICAM3, ULBP1, and ULBP2 was increased in cells expressing p50 K146Q compared with cells expressing p50 WT (Supplementary Fig. 5f). Moreover, increased levels of nuclear ACAT1 also promoted the expression of these genes (Supplementary Fig. 5g), suggesting that acetylation of p50 at K146 by nuclear ACAT1 facilitates NK cell activation. In addition, we verified the expression of genes that are well established to be involved in NK cell recruitment.^45^ IL17D, which was also detected by RNA-seq, as well as other chemokines, such as CCL5, CXCL10, and CXCL11, were obviously upregulated in p50 K146Q- or ACAT1 NLS-expressing cells (Supplementary Fig. 5f–g), indicating that nuclear ACAT1 can also stimulate the expression of chemokines via p50 K146 acetylation to attract NK cells.

Furthermore, subcutaneous tumor model revealed that mp50 K144Q (equivalent to K146Q in humans) clearly restricted tumor growth (Fig. 4i) and elevated the percentages of cytotoxic NK cells but not CD4^+^ or CD8^+^ T cells in tumors (Fig. 4j and Supplementary Fig. 5k), indicating that the antitumor capacity of p50 K146ac relies on NK cell activation. Of note, the antigen processing and presentation not only are the keystones of adaptive immunity but also contribute to NK cell activation.^46^ Therefore, we assumed that the antigen processing and presentation genes upregulated in response to the K146Q mutation partially support NK cell activation via crosstalk with adaptive immune cells. Taken together, these findings indicated that the DNA-binding capacity of p50 was impaired by K146 acetylation, thus promoting the transcription of genes related to NK cell activation and recruitment to inhibit tumor growth.

### ACAT1 pS60 facilitates its nuclear translocation

**Fig. 5:**
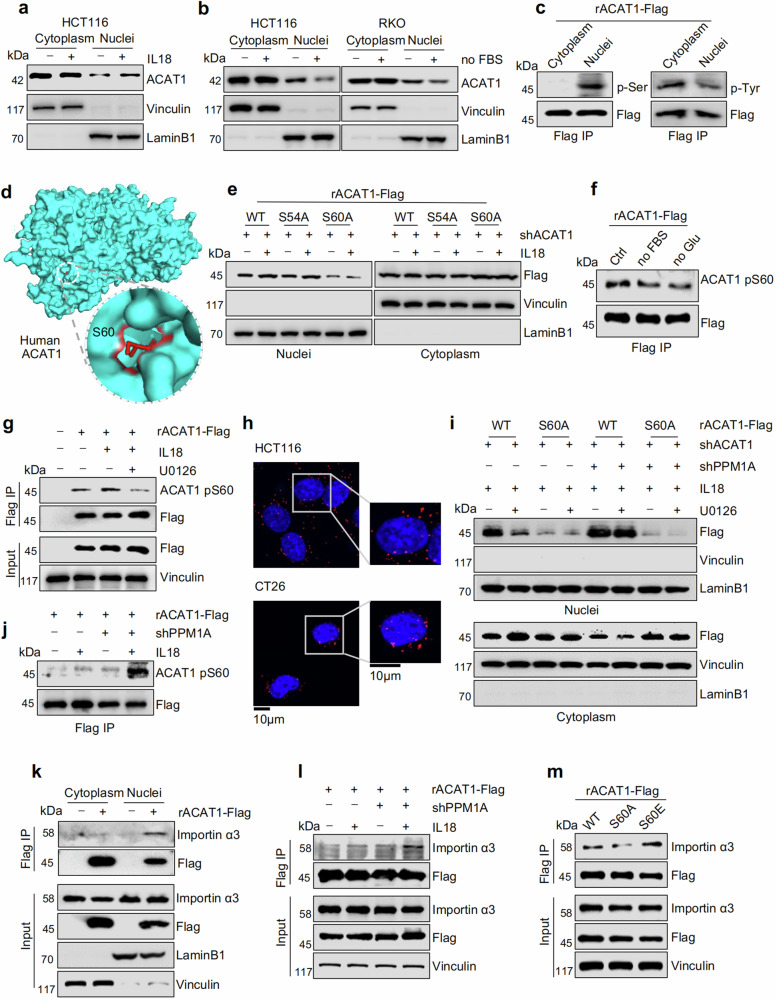
ACAT1 pS60 facilitates its nuclear translocation. **a** HCT116 cells were treated with or without IL18 for 12 h, and subcellular fractionation assay was subsequently performed. **b** HCT116 and RKO cells were cultured in RPMI-1640 medium with or without 10% fetal bovine serum (FBS) for 12 h, and subcellular fractionation assay was subsequently performed. **c** Subcellular fractionation assay was performed for IP analysis in HCT116 cells stably expressing ACAT1-Flag, and immunoblot analysis was then performed with the indicated antibodies. **d** Representative image of structure of S60 in human ACAT1 (PDB: 2F2S). Tetrameric ACAT1 is shown as surface pattern in blue, and S60 is shown as a red stick. **e** ACAT1-depleted HCT116 cells were rescued with ACAT1-Flag WT, S54A, or S60A and treated with or without 50 ng/ml IL18 for 12 h. Subcellular fractionation assay was performed for subsequent immunoblot analysis. **f** RKO cells transfected with ACAT1-Flag were cultured in RPMI-1640 medium supplemented with 10% dialyzed FBS (DFBS), or without DFBS and glucose separately for 12 h. ACAT1-Flag was immunoprecipitated with an anti-ACAT1 pS60 antibody to evaluate the phosphorylation level of S60. **g** HCT116 cells transfected with ACAT1-Flag were pretreated with or without 10 μM U0126 for 4 h before IL18 treatment for another 12 h. Immunoprecipitation assay was performed to detect the phosphorylation level of S60 with anti-ACAT1 pS60 antibody. **h** Representative images showing the interaction between endogenous PPM1A/Ppm1a and ACAT1-Flag/Acat1-Flag exogenously expressed in HCT116 cells and CT26 cells, as detected by the Duolink PLA assay. n = 3 biological replicates. **i** ACAT1-depleted HCT116 cells were rescued with ACAT1-Flag WT or S60A and infected with a lentivirus expressing shNC or shPPM1A and were then treated with IL18 or U0126. Subcellular fractionation assay was performed for subsequent immunoblot analysis. **j** HCT116 cells stably expressing ACAT1-Flag were infected with lentivirus expressing shNC or shPPM1A, followed by IL18 treatment or not. Immunoprecipitation assay was performed with an anti-ACAT1 pS60 antibody to evaluate the phosphorylation level of S60. **k** Subcellular fractionation assay was performed for IP in HCT116 cells stably expressing EV or ACAT1-Flag, and immunoblot analysis was then performed with the indicated antibodies to evaluate the interaction between ACAT1 and importin α3. **l** HCT116 cells stably expressing ACAT1-Flag were infected with lentivirus expressing shNC or shPPM1A, followed by IL18 treatment or not. IP and immunoblot analysis were performed with the indicated antibodies. **m** ACAT1-Flag was immunoprecipitated from HCT116 cells stably expressing ACAT1-Flag WT, S60A, or S60E. IP and immunoblot analysis were performed with the indicated antibodies. Immunoblots representative of three independent experiments are shown

We subsequently investigated how the nuclear translocation of ACAT1 is regulated. Considering that the level of nuclear ACAT1 is higher in peritumoral tissues than in CRC tumor tissues, we hypothesized that a benign immune microenvironment might facilitate ACAT1 nuclear localization. Therefore, we treated cells with a series of cytokines that promote antitumor immunity and found that IL12 and IL18, especially IL18, increased the nuclear ACAT1 level (Fig. 5a, and Supplementary Fig. 6a–e). Since nutrient deficiency is a common feature in the TME, we also treated CRC cells with medium lacking glucose, glutamine, or serum to mimic a nutrient-poor TME. As a result, glucose or serum deprivation dramatically decreased the nuclear ACAT1 level (Fig. 5b and Supplementary Fig. 6f). These findings indicate that the nuclear translocation of ACAT1 is a widespread event driven by the complex conditions in the TME.

Subcellular location of target protein is most often regulated by phosphorylation,^20^ so we compared the phosphorylation levels of cytoplasmic (including mitochondrial) and nuclear ACAT1. Unexpectedly, tyrosine-phosphorylated ACAT1 was concentrated in cytoplasm, whereas serine-phosphorylated ACAT1 was enriched in nucleus (Fig. 5c). Additionally, we observed increased level of serine-phosphorylated ACAT1 in HCT116, Caco2 and RKO cells treated with IL18, suggesting that IL18 promotes the nuclear translocation of ACAT1 by increasing its serine-phosphorylation (Supplementary Fig. 6g, h). The phosphoserine levels of target proteins are regularly modulated via the MEK1/2, PI3K, and JAK pathways; thus, we treated cells with inhibitors of these pathways. U0126 (a MEK1/2 inhibitor) decreased the level of serine-phosphorylated ACAT1and abrogated the translocation of ACAT1 from the mitochondria to the nucleus (Supplementary Fig. 6i–k), indicating serine phosphorylation is important to nuclear ACAT1. Therefore, we performed mass spectrometry analysis of nuclear ACAT1, and two residues S54 and S60 were found phosphorylated (Supplementary Fig. 7a). Coincidentally, the human ACAT1 structure downloaded from the Protein Data Bank (PDB, 2F2S) revealed that S60 but not S54 is located on the surface of ACAT1 (Fig. 5d), implying that S60 was more accessible for phosphorylation. Moreover, we independently mutated S60 and S54 to alanine (A) and found that S60A failed to respond to IL18 treatment, but ACAT1 WT and S54A were still stimulated to translocate to the nucleus (Fig. 5e). We then mutated S60 into glutamic acid (E) to mimic hyperphosphorylation and found that the level of nuclear ACAT1 was increased by S60E but decreased by S60A (Supplementary Fig. 7b, c). Furthermore, we generated an antibody to specifically identify ACAT1 phosphorylated at S60 (ACAT1 pS60) and tested the specificity (Supplementary Fig. 7d). After glucose or serum deprivation, the level of ACAT1 pS60 was dramatically decreased (Fig. 5f and Supplementary Fig. 7e). However, IL18 stimulation induced higher level of ACAT1 pS60, which was abolished by treatment with U0126 (Fig. 5g). These results indicate that ACAT1 pS60 facilitates its nuclear translocation.

To identify the direct regulator of ACAT1 pS60, we performed mass spectrometry analysis of the proteins bound to ACAT1 and identified protein phosphatase 1A (PPM1A). Duolink in situ proximity ligation assay (PLA) verified the interaction between ACAT1 and PPM1A (Fig. 5h). Additionally, the interaction between ACAT1 and PPM1A was attenuated by IL18 stimulation and reversed by U0126 (Supplementary Fig. 7f–h). Furthermore, after we depleted endogenous PPM1A, the nuclear ACAT1 WT was increased and could not be decreased by U0126. Besides, PPM1A depletion no more had effect on the location of ACAT1 S60A, despite pretreatment with U0126 (Fig. 5i and Supplementary Fig. 7i) but elevated the level of ACAT1 pS60 (Fig. 5j). Altogether, these results demonstrate that IL18 restricts PPM1A dephosphorylating ACAT1 S60.

Importin α functions in nuclear protein import and we fortunately found that ACAT1 bound to importin α3 (Fig. 5k and Supplementary Fig. 7j). Of note, the interaction between ACAT1 and importin α3 was strengthened by IL18 as well as PPM1A depletion, but was attenuated by U0126 (Fig. 5l and Supplementary Fig. 7k). Additionally, we observed increased binding of importin α3 to ACAT1 S60E relative to WT, which was decreased for S60A (Fig. 5m). In conclusion, these findings prove that ACAT1 pS60 upregulated by IL18 via alleviating the dephosphorylation functions of PPM1A promotes ACAT1 nuclear translocation, which could be inhibited by other environmental factors, such as nutrient deficiency.

### ACAT1 pS60 promotes NK cell activation and recruitment

**Fig. 6:**
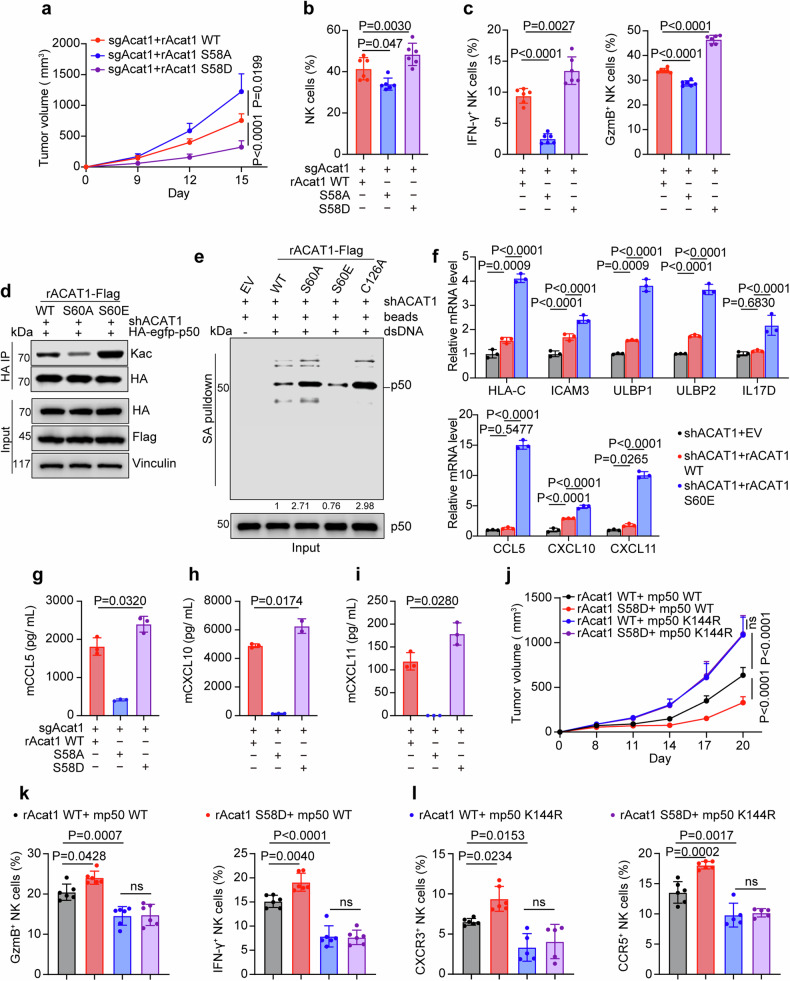
ACAT1 pS60 promotes NK cell activation and recruitment. **a**–**c** Acat1-KO CT26 cells rescued with Acat1-Flag WT, S58A or S58D were subcutaneously injected into BALB/c mice (6 mice per group). Tumor growth was measured (**a**). Flow cytometric analysis was performed 13 days after injection to calculate the percentage of tumor-infiltrating NK cells (**b**) and evaluate the expression of IFN-γ and GzmB (**c**) in the NK cells. **d** ACAT1-depleted HCT116 cells rescued with ACAT1-Flag WT, S60A, or S60E were co-transfected with HA-egfp-p50 (1-366aa), and HA-egfp-p50 was immunoprecipitated to evaluate its acetylation. **e** ACAT1-depleted HCT116 cells were rescued with ACAT1-Flag WT, S60A, S60E, C126A or EV (as a control). Subcellular fractionation assay and DNA pulldown assay were performed to detect nuclear p50 bound to biotinylated dsDNA (5 µg) conjugated to streptavidin magnetic beads. Each band was quantified via ImageJ. **f** ACAT1-depleted HCT116 cells were rescued with ACAT1-Flag WT or S60E and subjected to real-time qPCR analysis. n = 3 biological replicates. **g**–**i** Acat1-KO CT26 cells rescued with Acat1-Flag WT, S58A or S58D were cultured for 48 h, after which the supernatants were collected. Enzyme-linked immunosorbent assays (ELISAs) were then performed to quantify the secretion of mCCL5 (**g**), mCXCL10 (**h**), and mCXCL11 (**i**). n = 3 biological replicates. **j**–**l** Nfkb1-depleted CT26 cells rescued with HA-mp50 (1-364aa) or K144R were coinfected with lentiviruses expressing Acat1-Flag WT or S58D and subcutaneously injected into BALB/c mice (6 mice per group). Tumor growth was measured (**j**). Flow cytometric analysis was performed 13 days after injection to calculate the percentages of tumor-infiltrating IFN-γ^+^, GzmB^+^ NK cells (k) and CXCR3^+^, CCR5^+^ NK cells (**l**). Immunoblots representative of three independent experiments are shown. The data are shown as the means ± SDs. Two-way ANOVA (**a**, **f**, **j**) or one-way ANOVA (**b**, **c**, **g**–**i**, **k**, **l**)

To determine the role of pS60-dependent ACAT1 translocation in antitumor immunity, we generated Acat1-KO CT26 cells rescued with Acat1-Flag WT, S58A or S58D (aspartic acid, a hyperphosphorylation mimic), which is equivalent to S60A or S60E in humans (Supplementary Fig. 8a). Our subcutaneous tumor model showed that the tumor growth was clearly inhibited in S58D group but promoted in S58A group compared to the WT group (Fig. 6a and Supplementary Fig. 8b). Consistent with this finding, the proportions of tumor-infiltrating NK cells, including cytotoxic NK cells, were increased in S58D group but decreased in S58A group compared with WT group (Fig. 6b, c). Moreover, IHC staining with anti-NCR1 antibody revealed greater infiltration of NK cells in S58D group than in WT group, with the weakest infiltration observed in S58A group (Supplementary Fig. 8c). Given that the level of ACAT1 pS60 is regulated by PPM1A, we subsequently investigated whether PPM1A could restrain the antitumor effect of ACAT1 pS60. Notably, Ppm1a depletion suppressed tumor growth only in WT group but not S58A group (Supplementary Fig. 8d, e). Moreover, tumor-infiltrating NK cells exhibited the greatest increase in WT group after Ppm1a-depletion (Supplementary Fig. 8f). Taken together, these results suggest that ACAT1 pS60 facilitates activated NK cells to impair tumor growth.

Subsequently, we found that S60E dramatically increased the acetylation levels of p50, but S60A downregulated it compared to ACAT1-Flag WT (Fig. 6d). Moreover, DNA-bound p50 was enriched by S60A or C126A (a catalytically inactive mutant) but declined by S60E (Fig. 6e), indicating that ACAT1 pS60 could attenuate the binding of p50 to DNA by regulating its acetylation. Additionally, ACAT1 S60E obviously enhanced the expression levels of HLA-C, ICAM3, ULBP1, ULBP2 and IL17D, as well as chemokines CCL5, CXCL10 and CXCL11 (Fig. 6f). Meanwhile, the protein levels of IκBα and p50 and the location of p50 were not affected by S60A or S60E (Supplementary Fig. 8g, h). Moreover, ELISA assay verified that Acat1 S58D increased secretion of the chemokines CCL5, CXCL10, and CXCL11, which was abrogated by Acat1 S58A (Fig. 6g–i). We then knocked down CCL5 in Acat1-KO CT26 or MC38 cells, and conducted transwell migration assay of NK cells. As a result, CCL5 knockdown could totally inhibit the NK cell infiltration caused by rAcat1 NLS re-expressing in MC38 cells. In CT26 cells, CCL5 knockdown could decrease the overall level of the infiltrated NK cells and abolish the increase of NK cells caused by Acat1 WT compared to KO group. However, the difference in infiltrated NK cells between NLS group and WT group was just reduced, not abolished by shCCL5 (Supplementary Fig. 8j, k). The possible reason is that CCL5 is not the only downstream target of nuclear ACAT1 to promote NK cell infiltration, other chemokines such as CXCL10, CXCL11 should also take part in this process.

To further confirm that p50 K146ac contributed to the antitumor immune function of ACAT1 pS60, we induced Acat1-Flag WT or S58D expression in Nfkb1-depleted CT26 cells rescued with HA-mp50 (1-364aa) WT or K144R (equivalent to K146R in humans) and performed subcutaneous tumor model. When HA-mp50 WT expressing, Acat1 S58D significantly restrained tumor growth compared to Acat1 WT; however, when HA-mp50 K144R expressing, Acat1 S58D failed to inhibit tumor growth (Fig. 6j and Supplementary Fig. 8l). Along with these results, the proportions of tumor-infiltrating cytotoxic NK cells were boosted up by S58D expression in mp50 WT group, but declined in both of the HA-mp50 K144R groups, regardless of whether Acat1 WT or S58D was expressed (Fig. 6K). CXCL10 and CXCL11 are ligands for C-X-C chemokine receptor type 3 (CXCR3), and CCL5 is a ligand for C-C chemokine receptor type 5 (CCR5), which participates in T-cell and NK-cell recruitment.^45^ Hence, we evaluated the expression of CXCR3 and CCR5 on NK cell surface, and the same trends were observed as those for cytotoxic NK cells (Fig. 6L). Taken together, these findings demonstrate that ACAT1 pS60 enhances the abundance of tumor-infiltrating cytotoxic NK cells via p50 K146ac to inhibit tumor growth.

### Clinical relevance of the ACAT1 pS60 level in CRC

**Fig. 7:**
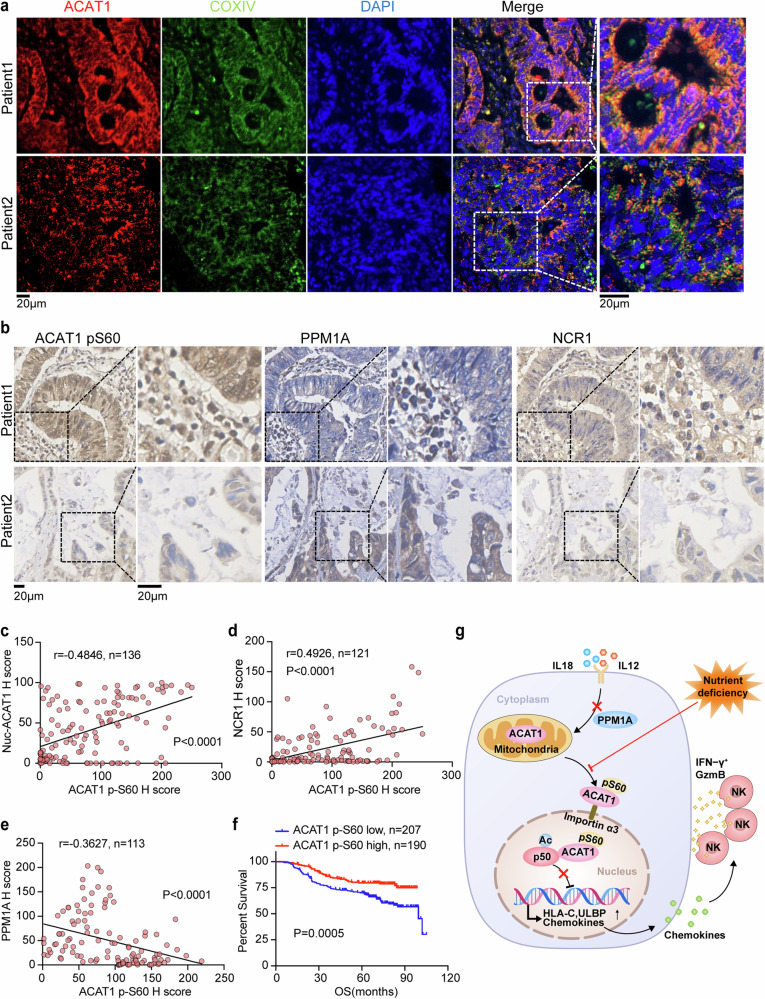
Clinical relevance of the ACAT1 pS60 level in CRC. IF analysis with anti-ACAT1 and anti-COX IV antibodies (**a**) and IHC analysis with anti-ACAT1 pS60, anti-PPM1A, and anti-NCR1 antibodies (**b**) of specimens from patients with colorectal cancer were performed. Representative images of IF and IHC staining of tumors from two patients with colorectal cancer are shown. **c**–**e** Semiquantitative scoring of IF and IHC staining was carried out via HALO, and the correlations between nuclear ACAT1 (IF) and ACAT1 pS60 (IHC) levels (**c**), NCR1 and ACAT1 pS60 levels (**d**), PPM1A and ACAT1 pS60 levels (**e**) were analyzed. **f** IHC analysis with anti-ACAT1 pS60 antibody was performed in tumor tissues of patients with colorectal cancer. Overall survival durations of 397 patients (stage1–4) with low (n = 207, blue curve) or high (n = 190, red curve) levels of ACAT1 pS60 were compared. **g** Mechanism through which ACAT1 pS60 promotes NK cell activation and infiltration to impair tumor growth. The data are shown as the means ± SDs. Pearson correlation test (**c**–**e**), two-tailed log-rank test (**f**)

To elucidate the clinical significance of our findings that ACAT1 pS60 facilitates NK cell infiltration and activation, IF analysis with anti-ACAT1, anti-COX IV antibodies and IHC analysis with anti-ACAT1 pS60, anti-PPM1A, and anti-NCR1 antibodies were performed on tumor tissues from patients with CRC. Notably, the ACAT1 pS60 level was strongly positively correlated with the nuclear ACAT1 level and the infiltration of NK cells but negatively correlated with the PPM1A level (Fig. 7a–e). In addition, a higher level of ACAT1 pS60 indicated a better overall survival in CRC patients (Fig. 7f), suggesting the prognostic value of ACAT1 pS60 in patients with CRC.

## Discussion

Over the past decades, extensive studies have strengthened the pivotal roles of metabolic reprogramming in tumor progression. Cancer cells undergo metabolic rearrangements to support their continuous proliferation and high energy demand. During this process, some abnormal metabolic enzymes or their downstream metabolites could affect gene expression to facilitate tumor immune evasion; also, tumor cells in hypermetabolic state might compete with immune cells for the nutrient substance, hampering the function of immune cells.^22^ While, how tumor metabolism influences surrounding immune cells in the TME remains poorly understood. Indeed, as indispensable effector cells of the innate immune system, NK cells can rapidly identify and kill infected or abnormally transformed cells. Moreover, the tumor cell recognition and killing capacity of NK cell are superior to T cell. Therefore, NK cells exhibit remarkable antitumor effects,^12–14^ and NK cell–based immunotherapies have shown excellent therapeutic effect in hematologic cancers. However, due to the complex tumor microenvironment, obstacle still remains when develop these therapies for solid tumors. Consequently, exploring how tumor metabolism impacts NK cells could provide valuable insights for the development of therapeutic approaches to overcome CRC. Considering that NK cells in the TME have been reported to exhibit different phenotypes, including dysfunctional states,^18,47^ we examined NK cells isolated from orthotopic tumors and performed Smart-seq2 to ensure that the elevated tumor-infiltrating NK cells induced by ACAT1 were activated. Furthermore, by mouse tumor models and flow cytometric analysis, we confirmed that ACAT1 promoted cytotoxic NK cell infiltration into tumors and impacted colorectal tumor growth.

Some studies have shown the oncogenic roles of ACAT1. For example, mitochondrial ACAT1 can acetylate PDP1 and PDHA1, thus downregulating the enzymatic activity of these two subunits of the pyruvate dehydrogenase complex (PDC) to promote glycolysis and support tumor growth.^28^ However, this conclusion was made without consideration of the immune environment. In contrast, another study demonstrated that ACAT1 exerts its antitumor effect on melanoma by increasing the abundance of cytokine-producing T cells in tumors;^48^ whereas, the mechanism by which ACAT1 activates antitumor immunity remains unknown. Here, we showed that ACAT1 was more abundant in cell nuclei in peritumoral tissues than in tumor tissues. Nuclear ACAT1 directly acetylated p50 at K146 and decreased its DNA-binding capacity, thus attenuating the transcriptional inhibitory effects of p50 homodimers. Indeed, as a subunit of NF-κB complex, p50 homodimers act mainly as transcriptional repressors, because they can recruit corepressors such as HDAC1 and Bcl-3 or can simply occupy a DNA sequence without TAD. As a result, chemokines such as CCL5, CXCL10, and CXCL11 are secreted to recruit NK cells when p50 fails in binding DNA sequence. Although these three chemokines were absent in our RNA-seq data, they have been confirmed as p50-regulating genes by many researchers previously. Moreover, ligands supporting NK cell activation, such as ULBP1 and ULBP2, as well as genes related to antigen processing and presentation signaling pathways, are upregulated to activate NK cells.

In fact, p50 homodimers are primarily considered as tumor suppressors, which seems to contradict our findings. However, p50 K146ac is not equivalent to loss or downregulation of p50, and we are inclined to explain this regulatory relationship as a site-specific regulatory model in which the expression of a set of genes related to NK cell recruitment and activation is upregulated under the control of nuclear ACAT1.

Accumulated studies elaborate the association between posttranslational modifications, especially phosphorylation, and protein subcellular translocation. Here, we find that the nuclear ACAT1 is enriched for serine phosphorylation, while mitochondrial ACAT1 is enriched for tyrosine phosphorylation, hinting that the upregulation of serine phosphorylation level might sustain the nuclear location of ACAT1. Further experiments verify that nuclear translocation of ACAT1 depends on its phosphorylation at S60. It is worth noting that ACAT1 pS60 could be upregulated by MEK1/2 pathway, which often exhibits high expression level in cancer. However, the phenomenon we observed in human CRC tissues is contradictory to this. The nuclear ACAT1 level in peritumoral tissues is relatively higher compared to tumor tissues, suggesting that multiple kinases may regulate ACAT1 pS60. In early stage of tumor tissues or normal tissues, where the microenvironment is still subjected to immune surveillance, cytokines secreted by immunocytes, such as IL18 and IL12, can stimulate nuclear translocation of ACAT1 to strengthen antitumor immunity. While, characteristics of the malignant TME such as nutritional deficiency and glucose deprivation can downregulate ACAT1 pS60 to promote immune evasion. These findings indicate that nuclear ACAT1 level doesn’t depend on MEK1/MEK2 alone under the complex environmental conditions, but on multiple signals that collectively regulate the status of ACAT1 pS60 to determine its subcellular localization. Moreover, we identified PPM1A as an upstream phosphatase of ACAT1 pS60 and found that it was negatively regulated by IL18 signaling. However, other regulators modulating ACAT1 pS60 may exist, and we are optimistic that PPM1A or other yet-unidentified regulators will hold promise for inhibiting colorectal tumor growth via specific induction of ACAT1 nuclear translocation.

In summary, our study demonstrates that mitochondrial ACAT1 encourages NK cell infiltration and activation via its phosphorylation-dependent nuclear function. Nuclear ACAT1 directly acetylates p50 at K146 and decreases the DNA-binding capacity of p50, thus attenuating its transcriptional inhibitory functions and facilitating NK cell recruitment and activation (Fig. 7g). Notably, a low level of ACAT1 pS60 corresponds to a low abundance of NK cells in CRC tumor tissues and unfavorable outcomes in patients with CRC, indicating the prognostic value of ACAT1 pS60. Together, our findings highlight a novel function of ACAT1 as a nuclear protein acetyltransferase and delineate its role in NK cell-dependent antitumor immunity through p50 acetylation. Additionally, we expect that CRC patients with higher levels of nuclear ACAT1 and ACAT1 pS60 may benefit from NK cell-based immunotherapy.

## Materials and methods

### Ethics statements

For animal study, all the related experimental procedures were approved by the Institutional Animal Care and Use Committee of Sun Yat-Sen University. The ethics approval ID is L102012023001A.

The use of human CRC tissues and paired peritumoral normal tissues was approved by the institutional review board at Sun Yat-sen University Cancer Center and followed all relevant ethical regulations. The ethics approval ID is G2023-287-01.

### Cell culture

HEK293T, Caco2 and MC38 cells were cultured in Dulbecco’s modified Eagle’s medium (Gibco) supplemented with 10% fetal bovine serum (FBS) and 1% penicillin-streptomycin (Gibco). CT26, B16F10 and HCT116 cells were cultured in RPMI-1640 medium (Gibco) supplemented with 10% FBS and 1% penicillin-streptomycin. RKO cells were cultured in Minimum Essential Medium (Gibco) supplemented with 10% FBS, 1% non-essential amino acids solution (NEAA), and 1% penicillin-streptomycin. All the cell lines were confirmed without mycoplasma contamination. All cells were incubated at 37 °C in a humidified incubator with 5% CO_2_.

ACAT1 knockout cell clones were generated by CRISPR-Cas9 gene editing technology. In brief, CT26, MC38 and B16F10 cells were infected with lentivirus carrying pCRISPR-LvSG06 (GeneCopoeia, MCP238089-LvSG06-3-10-c) constructs with guide sequences (TGGATGCATAACTTCGTTCC) and incubated with 8 μg/ml polybrene for 24 h. Puromycin was used to select the virus-infected cells 48 h after infection, and single clones were then propagated for further analysis.

### Construction of relevant plasmids and lentivirus

Short hairpin RNA (shRNA) was constructed via ligation of an oligonucleotide targeting human ACAT1 into pCLenti-U6-shRNA-CMV-Puro-WPRE vector (OBiO). The shRNA for human NFKB1, PPM1A or mouse Nfkb1, Ppm1a, CCL5 were generated with pLVX-shRNA-Neo, pLKO.1-shRNA-Puro.

Polymerase chain reaction (PCR)-amplified human ACAT1 or its mutants was cloned into the pCDNA3.1-FLAG or pLVX-Neo-Flag vector. PCR-amplified mouse Acat1 or its mutants was cloned into the pLVX-Neo-Flag vector. PCR-amplified human EGFP-p50 (1-366aa), p50 (1-366aa), or its mutants was cloned into the pCDNA3.1-HA vector. PCR-amplified mouse p50 (1-364aa) or its mutants was cloned into the pCDH-Puro-HA vector.

Lentiviral particles were generated from HEK293T cells co-transfected with expressing vectors (pLVX, pLKO.1 pCDH, or sgRNA), and packaging vectors (psPAX2 (Addgene, 12260) and pCMV-VSV-G (Addgene, 8454)) using PEI at a 1:3 ratio. The supernatant was collected 48–72 h after transfection and filtered through a 0.45 μm filter to obtain the viral stock.

### Cell transfection and lentivirus infection

For transient transfection, cells were plated at appropriate density 18 h before transfection. The plasmid and DNA transfection reagent were added with a 1:1 ratio into the Opti-MEM. The mixture was mixed thoroughly and incubated 15–20 min at room temperature before added into cell media. And the subsequent experiments could be performed 48 h after transfection.

For lentivirus infection, cells were infected with lentivirus and incubated with 8 μg/ml polybrene for 24 h. Puromycin or neomycin were used to select the infected cells.

### Animal studies

Six-week-old female BALB/c, C57BL/6J, or five-week-old female NOD-scid IL2Rg^null^ (NSG) mice were purchased from Beijing Vital River Laboratory Animal Technology Co., Ltd. and maintained under pathogen-free standard conditions.

### Orthotopic model of colon cancer

BALB/c or C57BL/6J mice (six-week-old, female) were anaesthetized and placed in the supine position. 2 × 10^6^ luciferase-expressing CT26 cells or MC38 cells were resuspended in PBS with an equal volume of growth factor-reduced Matrigel in a total volume of 100 μL and injected into the cecum of the mice with a 30-gauge needle. About 16 days (BALB/c mice) or 18 days (C57BL/6J mice) after inoculation, bioluminescence imaging was performed by a Tanon-5200 Chemiluminescent Imaging System (Tanon). Then the tumors were dissected from mice for flow cytometry analyses or fixed in 4% formaldehyde for H&E staining.

### Flow cytometry analysis of tumor-infiltrating mouse lymphocytes (TILs)

Tumors were dissected into small pieces and digested with enzymes A, R and D from the Mouse Tumor Dissociation Kit (Miltenyi Biotec) and incubated on a rotator at 37 °C for 30 min. Digested tumors were then filtered through 70 μm strainer and washed with MACS buffer (PBS containing 2% FBS). Then, cells were resuspended in 40% Percoll and centrifuged to obtain immunocytes for subsequent analysis.

Before staining of cell surface molecules, cells were pre-blocked with rat serum and stained with indicated antibodies for 30 min at 4 °C.

For staining of intracellular cytokines or granules, cells were stimulated for 3 h with cell stimulation cocktail (plus protein transport inhibitors), and followed by surface markers staining. After fixation and permeabilization with BD Cytofix/Cytoperm™ solution according to manufacturer’s instructions, cells were stained with indicated antibodies. Samples were then analyzed with BECKMAN COULTER CytoFLEX LX cell analyzer and CytoExpert software version 2.0.

### Tumor infiltrating NK cells isolation

To isolate tumor-infiltrating NK cells from mice, tumors were dissected from ceca were cut into small pieces and subjected to enzymatic digestion. The digestion solution was filtered with 70 μm strainer and centrifuged at 500 × *g* for 5 min. Cells were resuspended in MACS buffer and were Fc-blocked for 10 min. And then, cells were stained with Zombie UV™ Fixable Viability Kit following the manufacturer’s instruction and incubated with primary antibodies as follows: APC-CD45, FITC-CD3 with PE/Cyanine7-NKp46 for 30 min at 4 °C. NK cells (CD3^-^ NKp46^+^) were sorted by fluorescence-activated cell sorting (FACS) using MoFlo Astrios (BECKMAN). The purity (>98%) of sorted cells was checked on BECKMAN COULTER CytoFLEX LX cell analyzer.

### Analyses of metabolic enzymes correlated to NK cell infiltration

To obtain the metabolic enzymes positively correlated with the levels of tumor-infiltrating NK cells in CRC patients, we first filtered the 1773 metabolic genes with the survival analysis, and about only 570 genes associated with survival ability were chosen. Then, 217 genes which solely benefited survival in condition of high infiltration level of NK cells but not low infiltration level of NK cells were picked. At last, because we mainly concerned the protein levels of the metabolic enzymes, we matched these 217 genes with the condition that the target proteins must possess statistical differences in mRNA and protein expression levels between normal and tumor tissues, and finally 81 proteins were obtained.

The clinical information and mRNA expression data was collected from the TCGA COADREAD cohort. The relative frequency of NK cells in TCGA COADREAD tumors were analyzed by MCPcounter (Microenvironment Cell Populations counter). The data of protein expression was loaded from Vasaikar and colleagues^49^ study in CRC. Survival analysis of patients between ACAT1 high expression and low expression with CRC stratified by the infiltration levels of NK cells was based on MCPcounter analysis results. The optimal cutoff was defined as the point with the most significant (log-rank test) split. The Kaplan–Meier survival curves were generated using Prism 9 software with log-rank test.

## Quantification and statistical analysis

Data are shown as mean ± SD. All statistics were performed using GraphPad Prism software (v.8.0). P < 0.05 was considered to be statistically significant. Three independent biological replicates have been performed for each experiment. The comparisons between two groups were performed with a two-sided unpaired Student’s t test. ANOVA models were performed for the comparisons of continuous outcomes across multiple experimental groups. Kaplan-Meier methods and log rank test were used to assess the difference in overall survival. Pearson correlation was used to assess the correlation between two variables.

## Supplementary information

The original and uncropped films of Western blots

supplementary materials new

Supplementary Fig.1

Supplementary Fig.2

Supplementary Fig.3

Supplementary Fig.4

Supplementary Fig.5

Supplementary Fig.6

Supplementary Fig.7

Supplementary Fig.8

Supplementary Table1

Supplementary Table2

Supplementary Table3

Supplementary Table4

Supplementary Table5

Supplementary Table6

Supplementary Table7

Supplementary Table8

Supplementary Table9

Supplementary Table10

Supplementary Table11

## Data Availability

All sequencing data involved in this study have been deposited in the Gene Expression Omnibus database under accession number GSE291785. The mass spectrometry proteomics data have been deposited to the ProteomeXchange Consortium (https://proteomecentral.proteomexchange.org) via the iProX partner repository with the dataset identifier PXD061959.
