# Supplementary material for: Nuclear mitochondrial acetyl-CoA acetyltransferase 1 orchestrates natural killer cell-dependent antitumor immunity in colorectal cancer
Source: Signal Transduct Target Ther. 2025 Apr 28;10:138. doi: 10.1038/s41392-025-02221-y (PMC12034769; doi:10.1038/s41392-025-02221-y)

Fig. 3

Fig. 3b

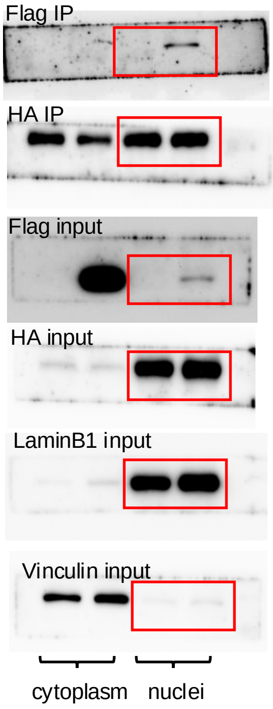

Fig. 3c

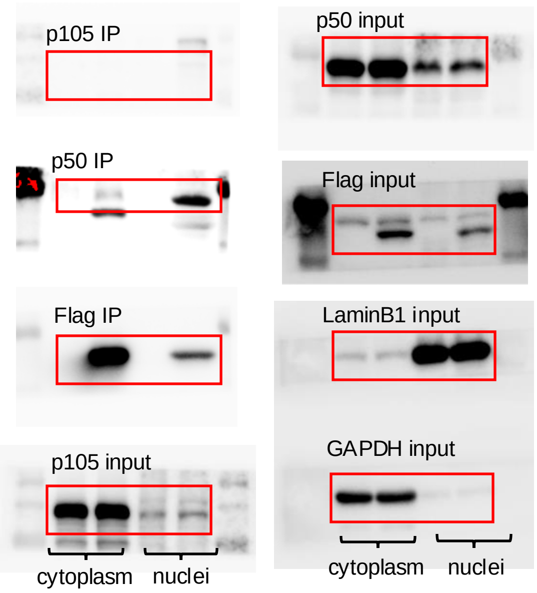

Fig. 3d HCT116

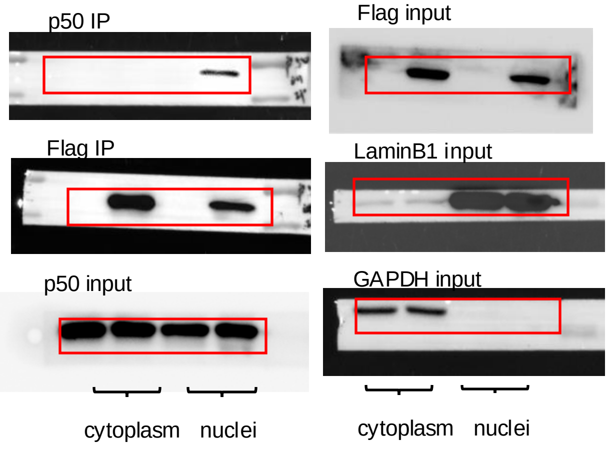

Fig. 3d CT26

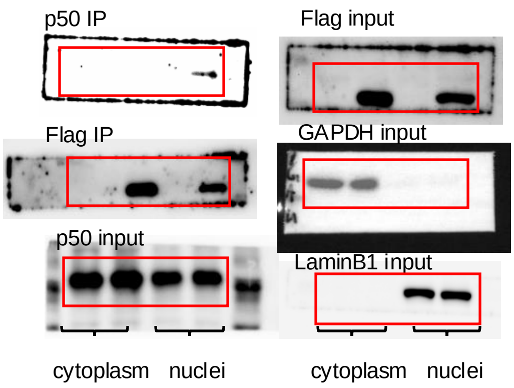

Fig. 3e left

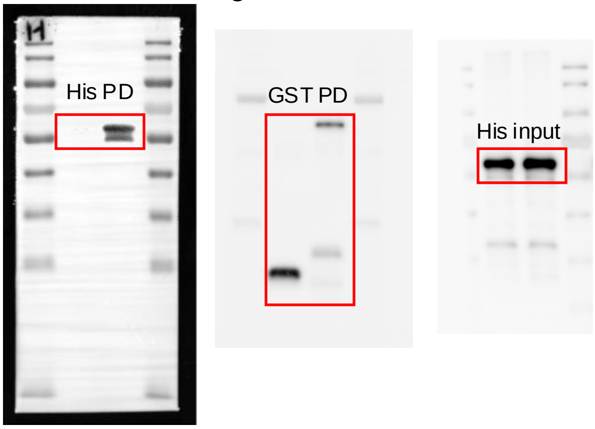

Fig. 3e right

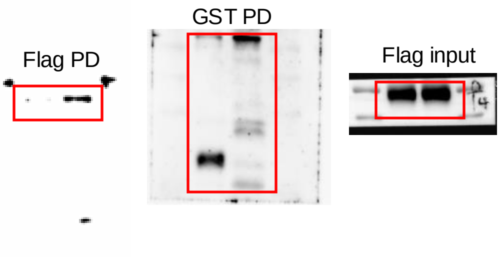

Fig. 3

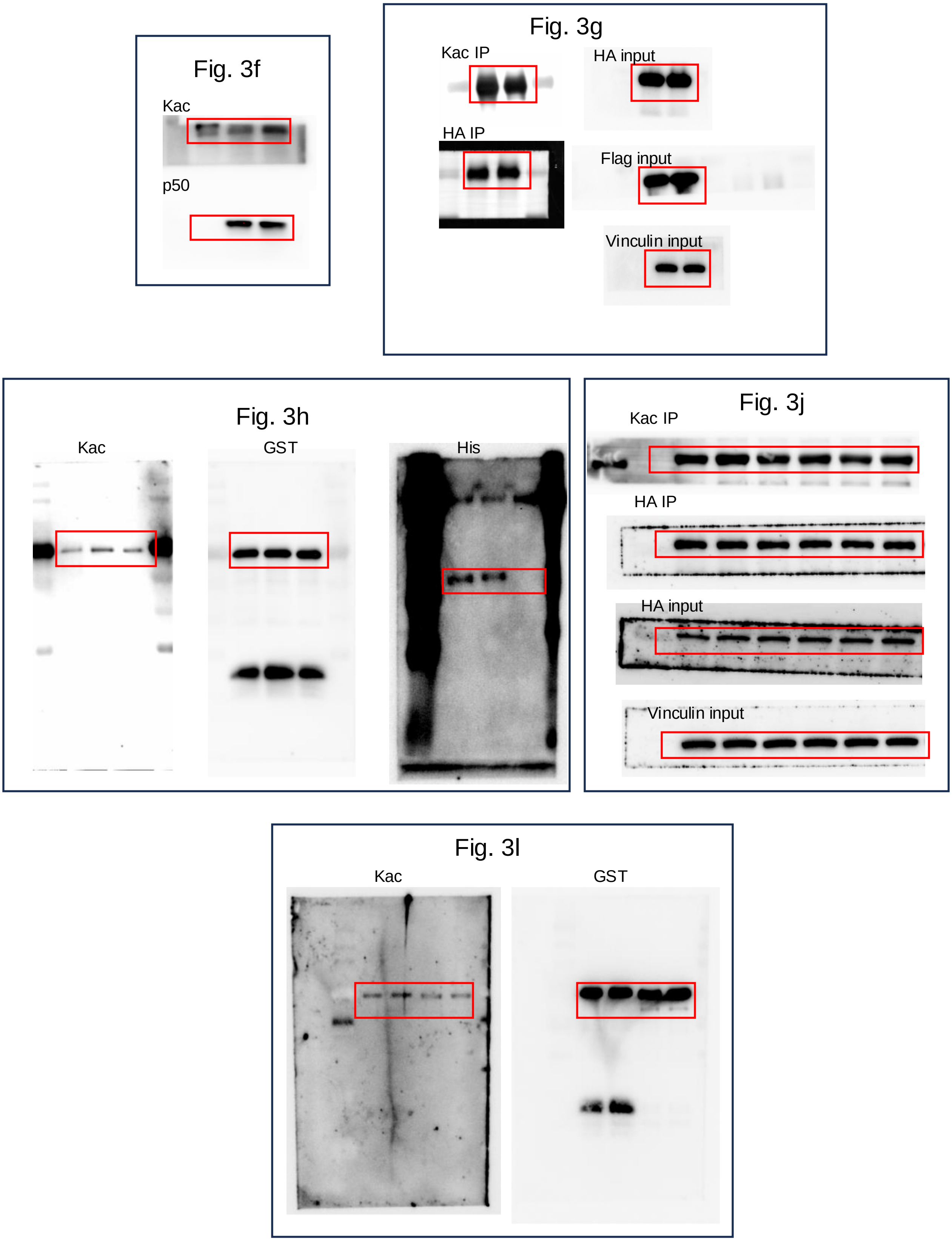
Fig. 3f
Kac
p50
Fig. 3g
Kac IP
HA input
HA IP
Flag input
Vinculin input
Fig. 3h
Kac
GST
His
Fig. 3j
Kac IP
HA IP
HA input
Vinculin input
Fig. 3l
Kac
GST

Fig. 4

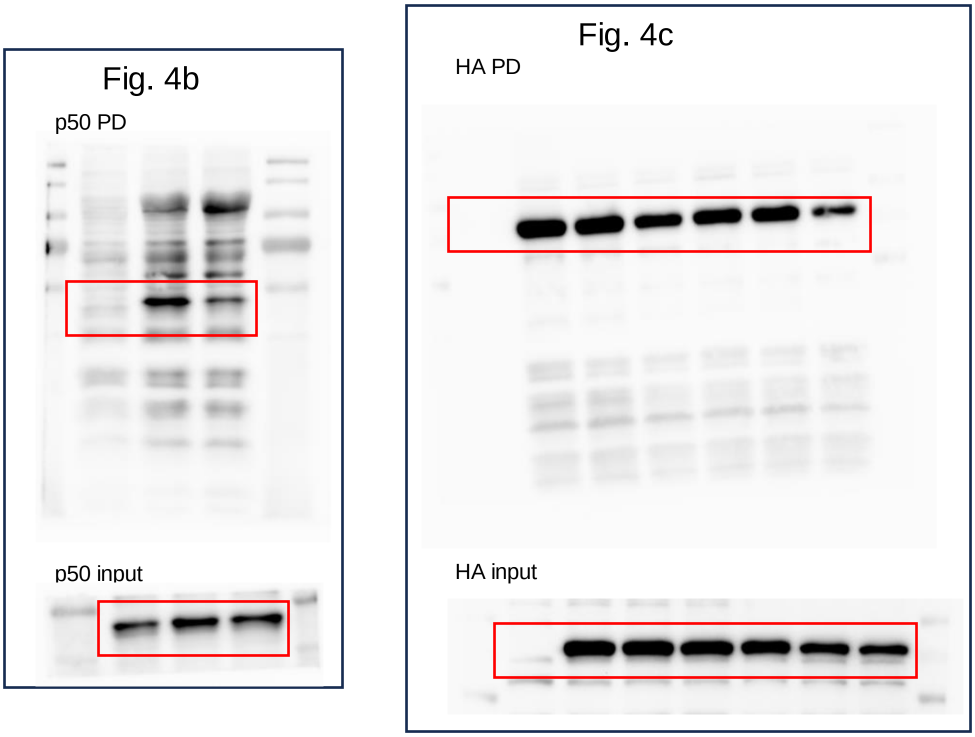

Fig. 5

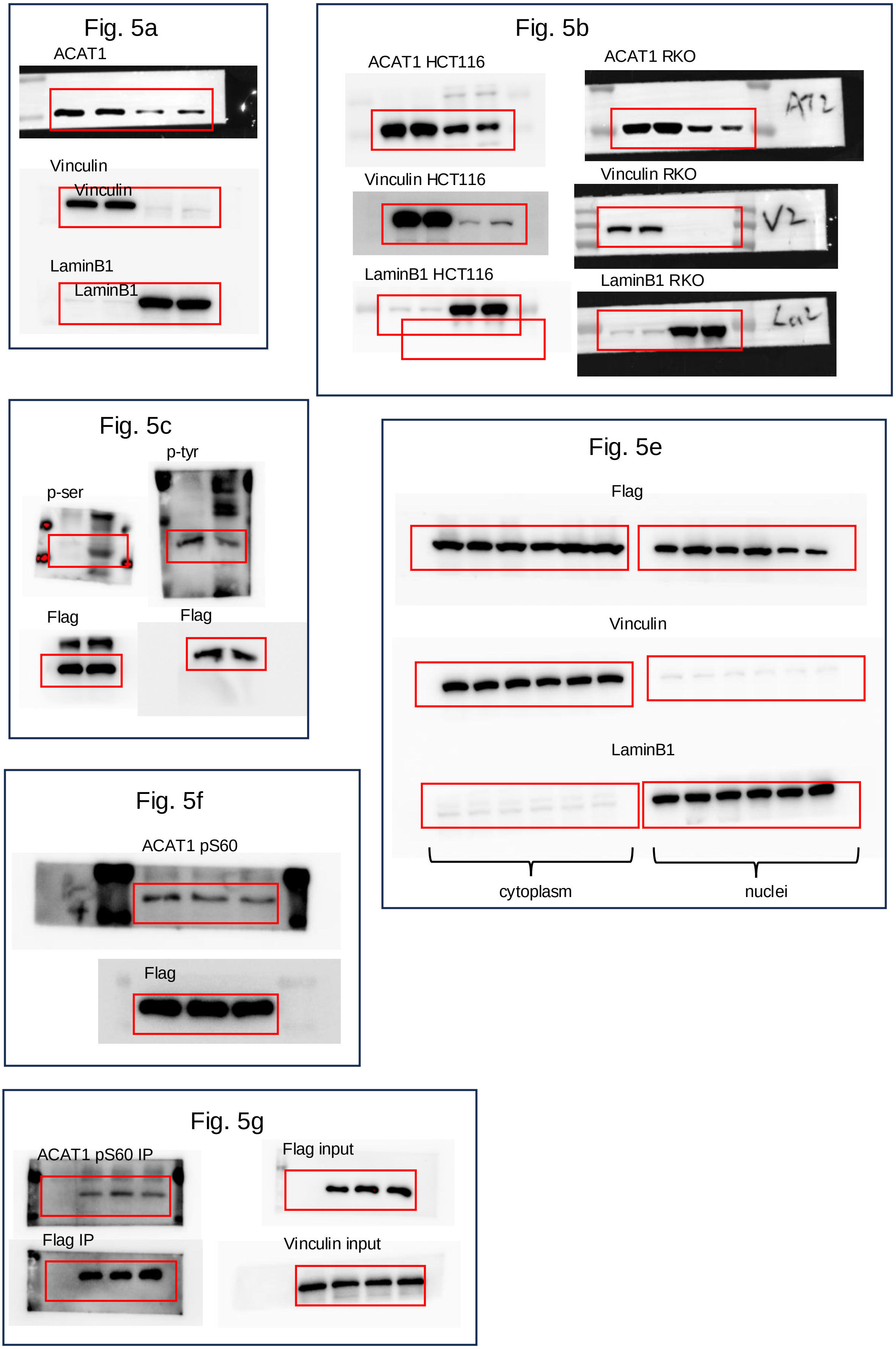
Fig. 5a
ACAT1
Vinculin
Vinculin
LaminB1
LaminB1
Fig. 5b
ACAT1 HCT116
ACAT1 RKO
Vinculin HCT116
Vinculin RKO
LaminB1 HCT116
LaminB1 RKO
Fig. 5c
p-tyr
p-ser
Flag
Flag
Fig. 5e
Flag
Vinculin
LaminB1
cytoplasm
nuclei
Fig. 5f
ACAT1 pS60
Flag
Fig. 5g
ACAT1 pS60 IP
Flag input
Flag IP
Vinculin input

Fig. 5

Fig. 5i

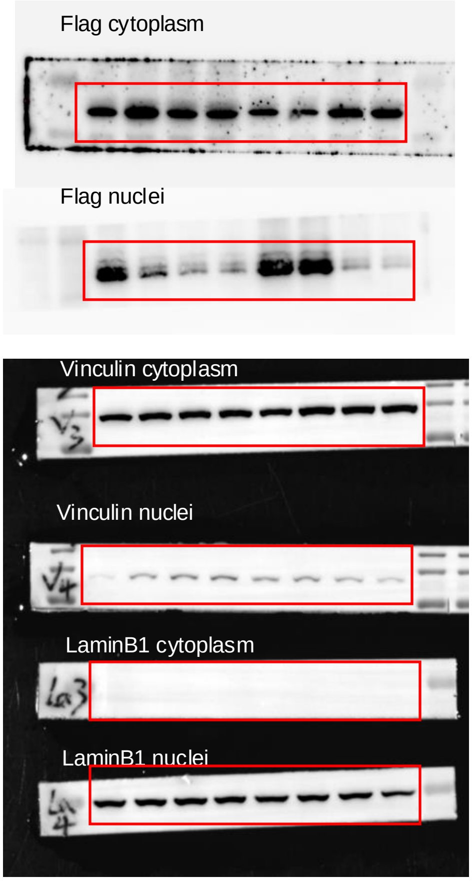

Fig. 5j

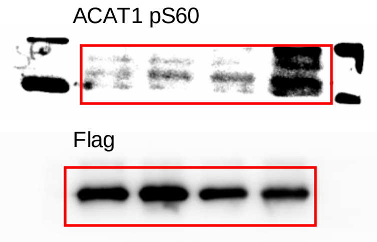

Fig. 5k

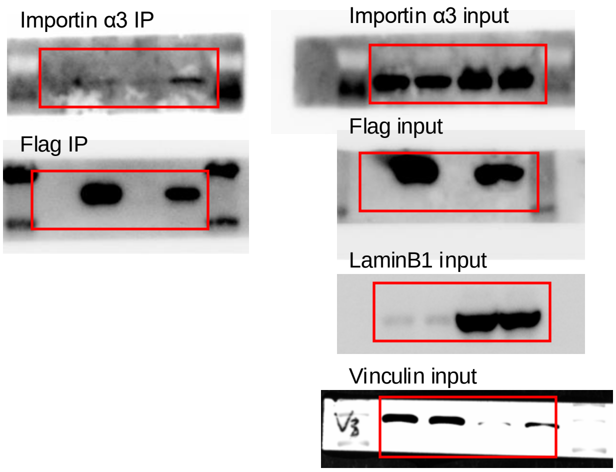

Fig. 5l

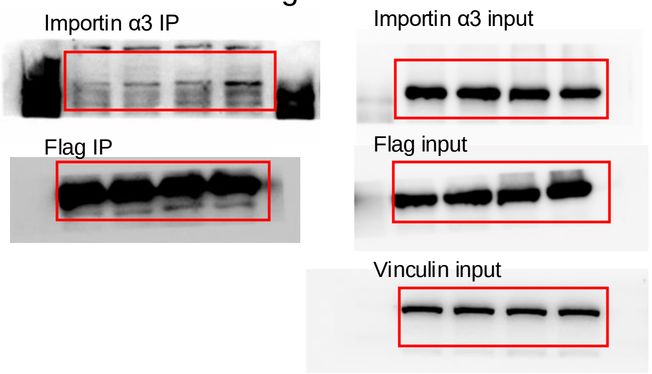

Fig. 5m

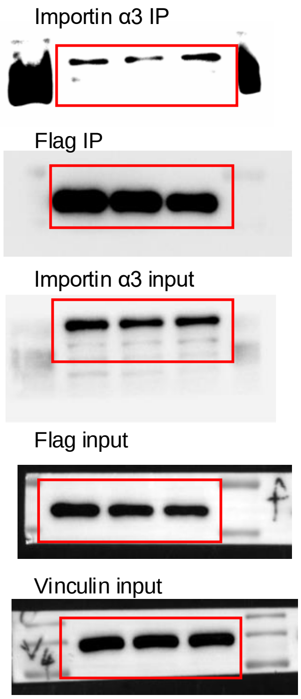

Fig. 6

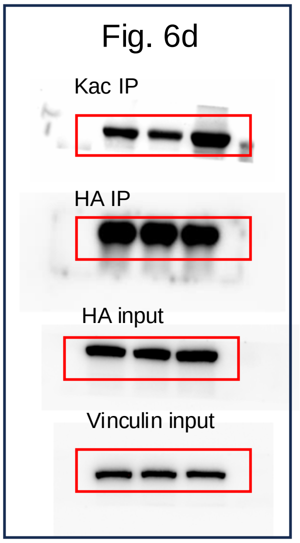
Fig. 6d
Kac IP
HA IP
HA input
Vinculin input

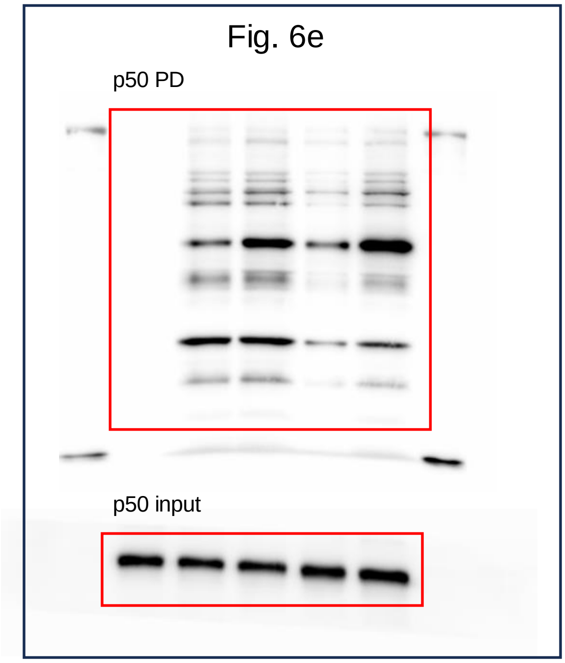
Fig. 6e
p50 PD
p50 input

Supplementary Fig. 1

Supplementary Fig. 1d

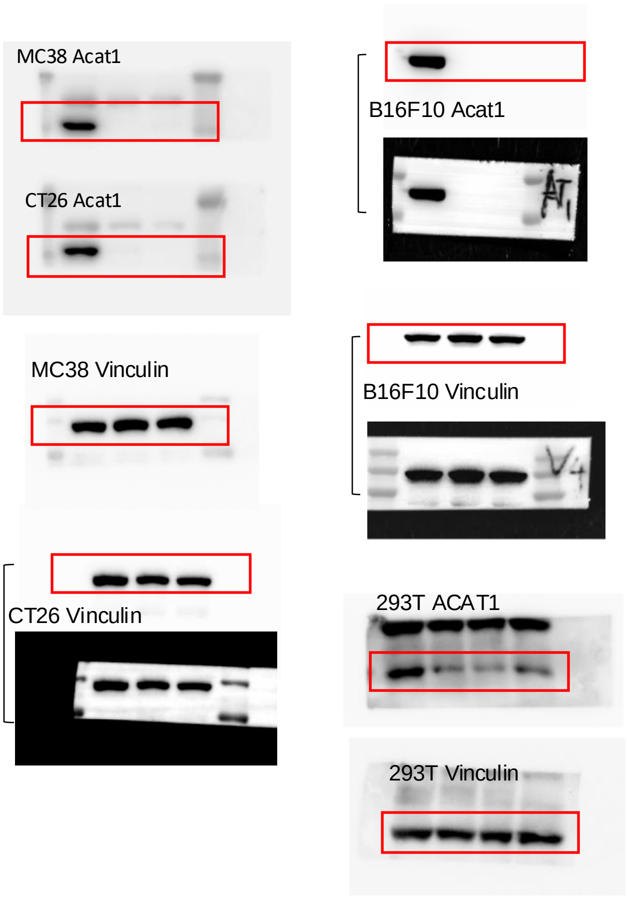

Supplementary Fig. 1e

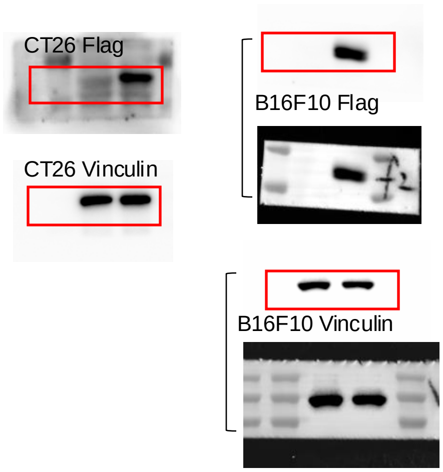

Supplementary Fig. 1f

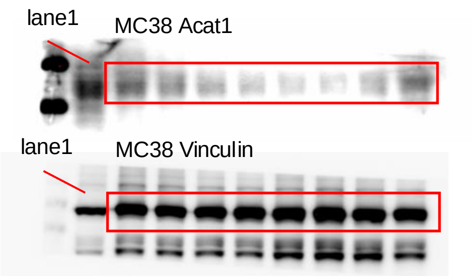

Lane1: a positive control, which we infected with the maximum volume of rAcat1 virus. However, the strong signal of the lane1 could interfere the exposure image of others lanes. In order to present more beautiful WB image, we cut out the lane1

Supplementary Fig. 2

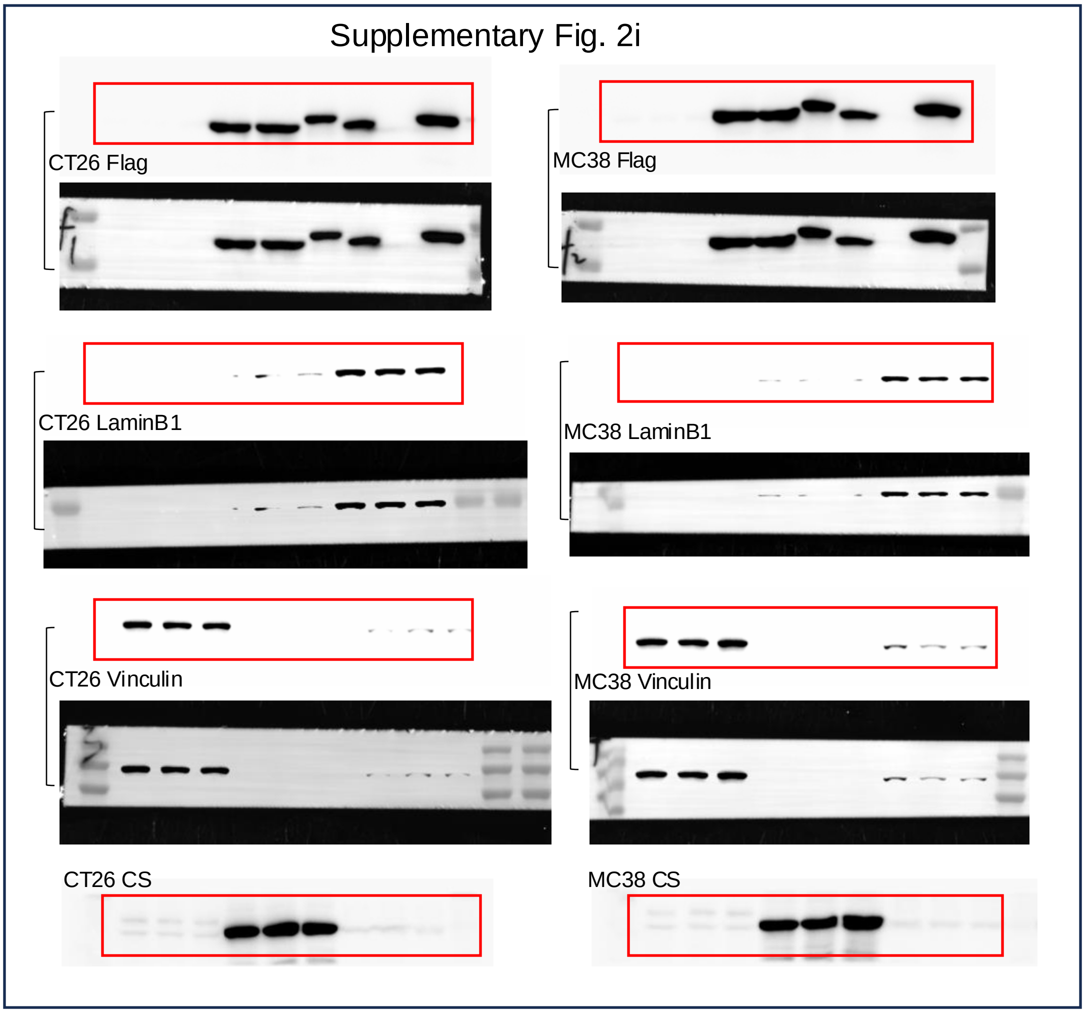

Supplementary Fig. 3

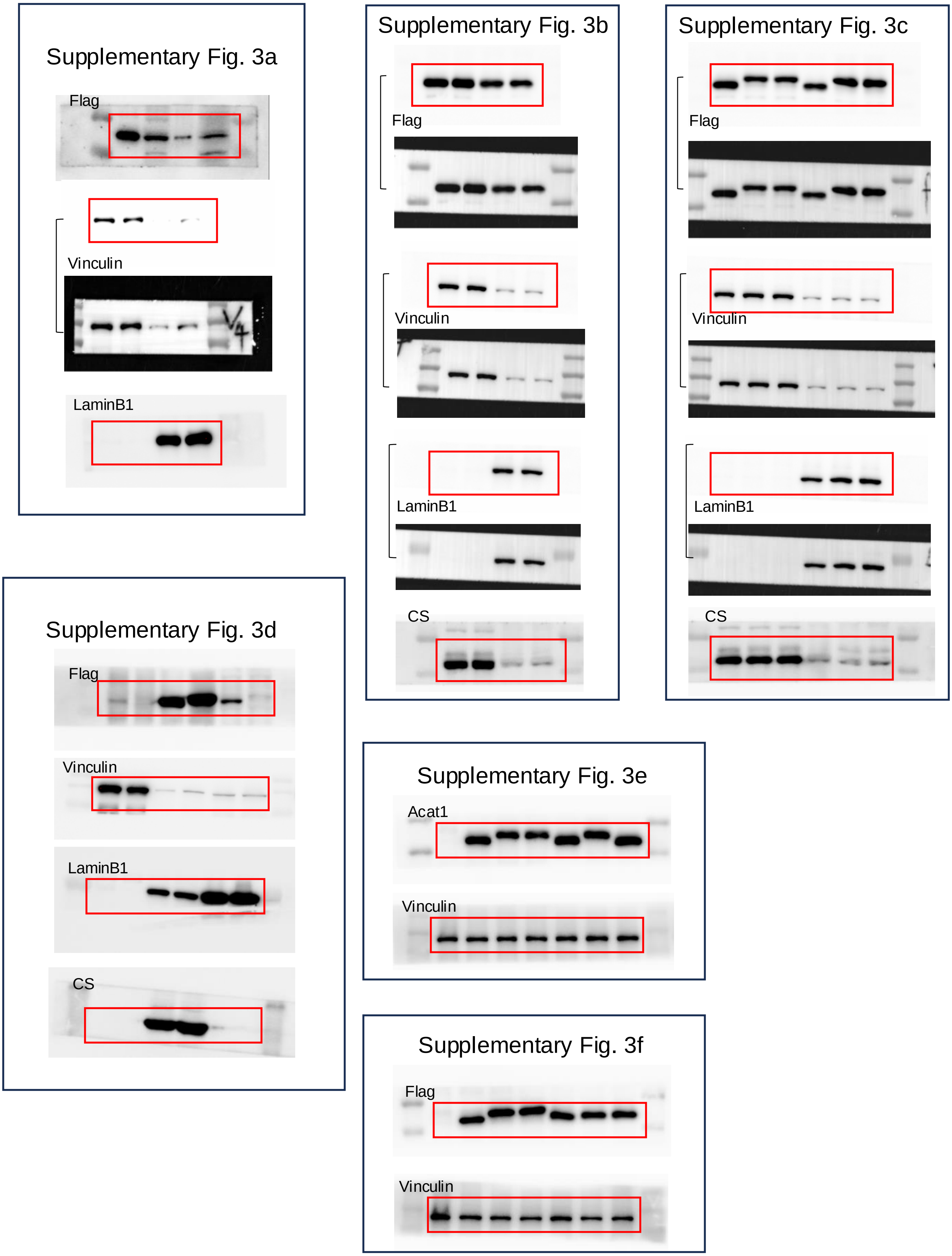

Supplementary Fig. 4

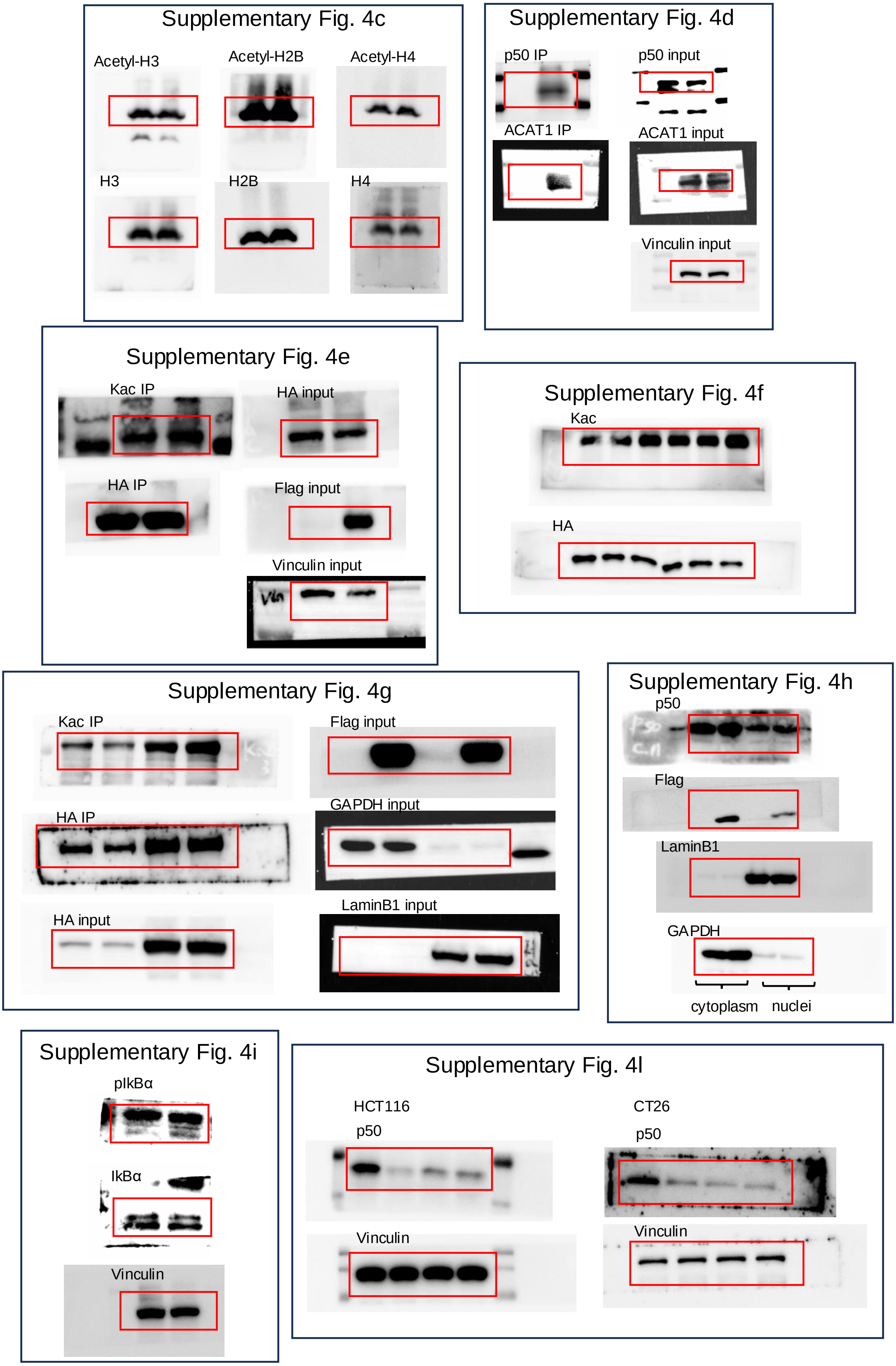

Supplementary Fig. 6

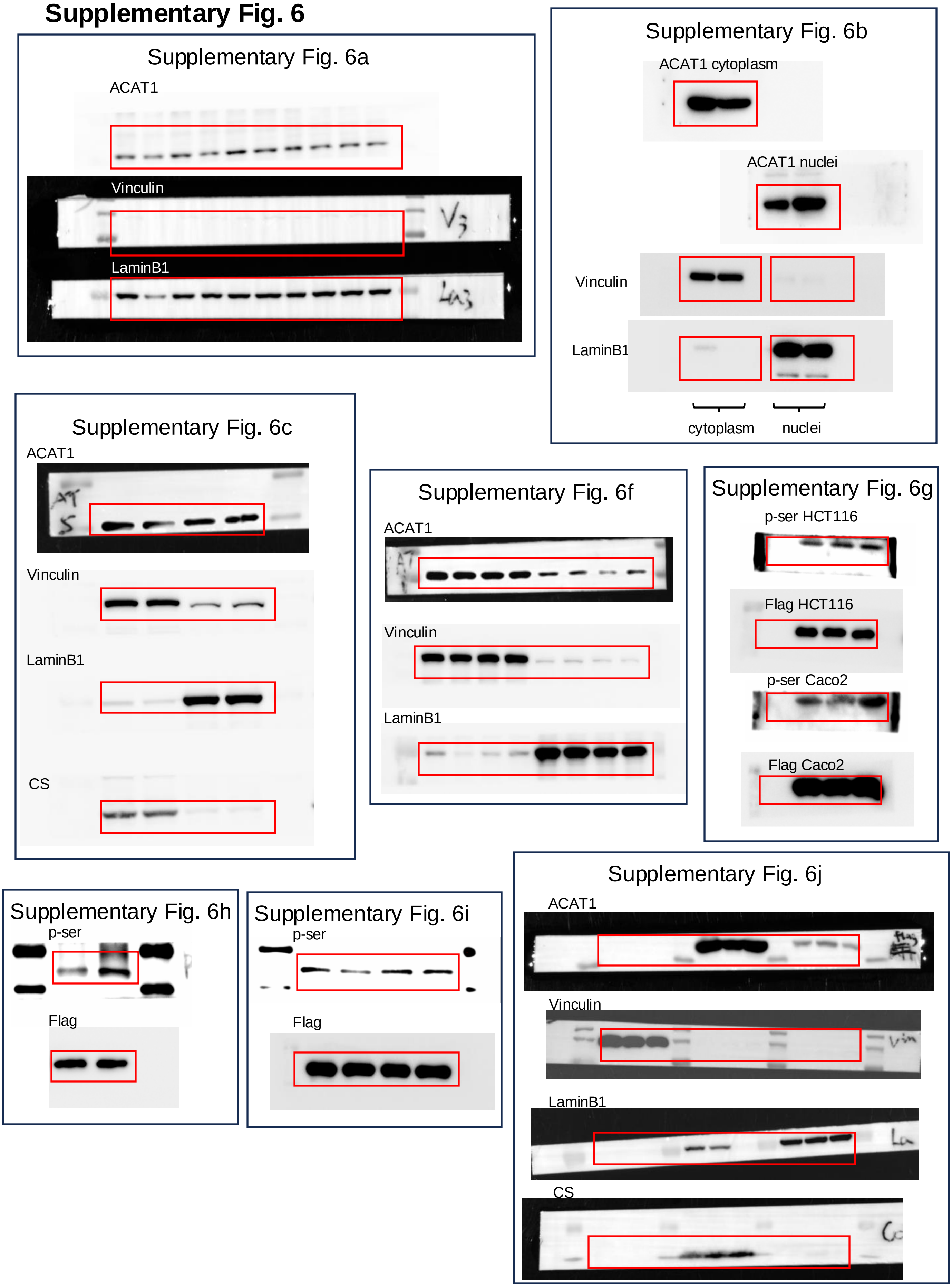

Supplementary Fig. 6

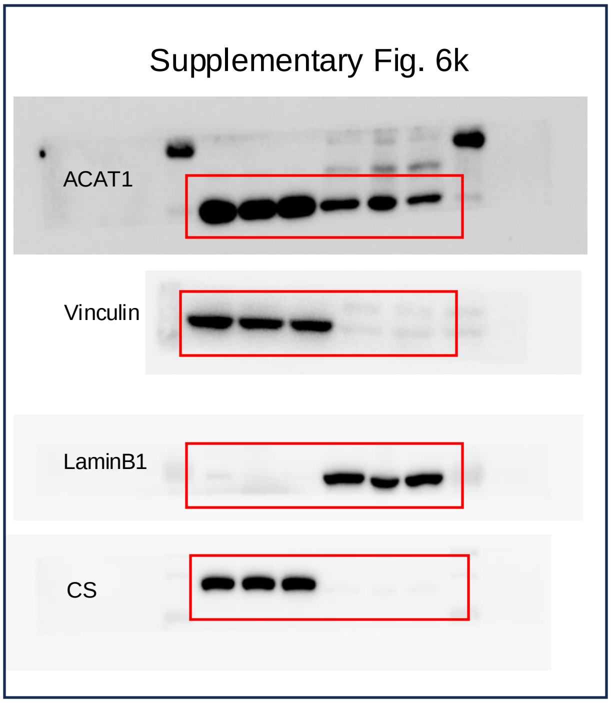

Supplementary Fig. 7

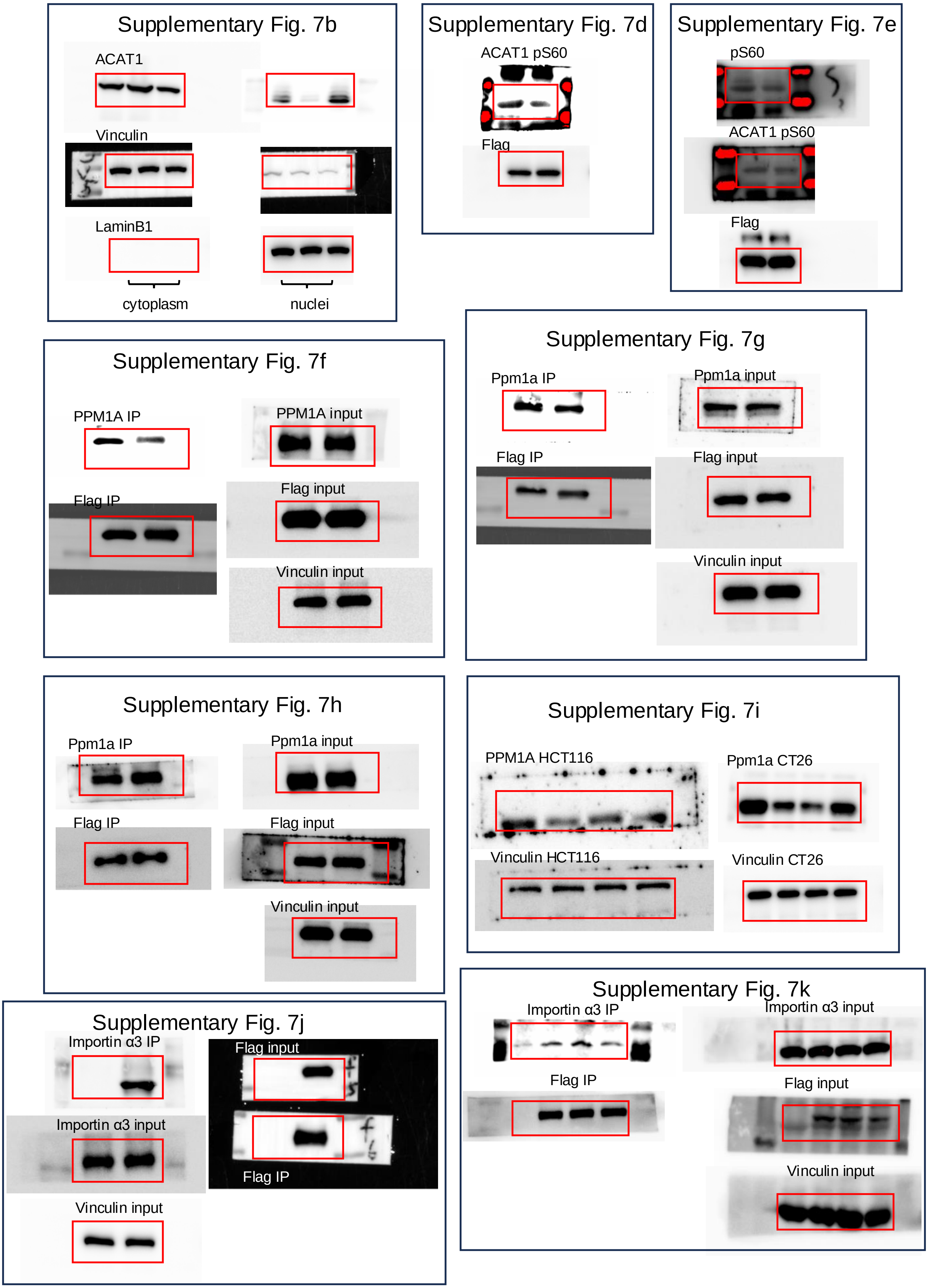

Supplementary Fig. 8

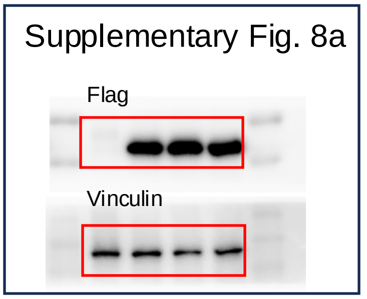

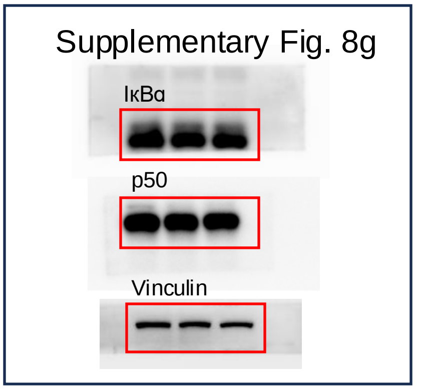

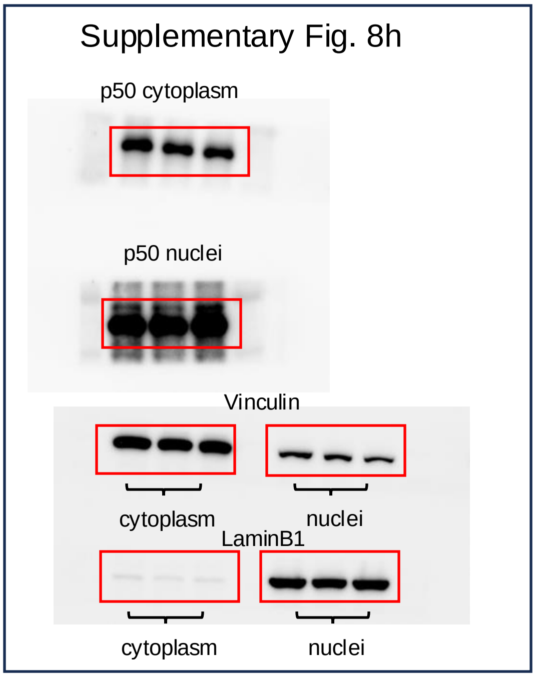

Supplement: Supplementary file 1 — The original and uncropped films of Western blots [file 41392_2025_2221_MOESM1_ESM.pdf]
