# Supplementary material for: Nuclear mitochondrial acetyl-CoA acetyltransferase 1 orchestrates natural killer cell-dependent antitumor immunity in colorectal cancer
Source: Signal Transduct Target Ther. 2025 Apr 28;10:138. doi: 10.1038/s41392-025-02221-y (PMC12034769; doi:10.1038/s41392-025-02221-y)
Supplement: Supplementary file 2 — supplementary materials new [file 41392_2025_2221_MOESM2_ESM.docx]

Supplementary Materials for

**Nuclear mitochondrial acetyl-CoA acetyltransferase 1 orchestrates natural killer cell-dependent antitumor immunity in colorectal cancer**

Chen Wei^1,*^, Kun Liao^1,*^, Hao-Jie Chen^1,*^, Zi-Xuan Xiao^1,*^, Qi Meng^1,*^, Ze-Kun Liu^1^, Yun-Xin Lu^1^, Hui Sheng^1^, Hai-Yu Mo^1^, Qi-Nian Wu^1^, Yi Han^1^, Zhao-Lei Zeng^1,2^, Xin-Yuan Guan^3^, Hui-Yan Luo^1,2^, Huai-Qiang Ju^1,3,#^, Rui-Hua Xu^1,2, 4,#^

Correspondence to: [juhq@sysucc.org.cn,](mailto:juhq@sysucc.org.cn,) [xurh@s](mailto:xxxxx@xxxx.xxx)ysucc.org.cn.

**This PDF file includes:**

Materials and Methods

Supplementary Fig. 1 to 8 (with figure legends below picture)

Supplementary Table 1 to 11 (These table files are uploaded as “Data set” form, and we provide table titles in “**Other Supplementary Materials for this manuscript include the following” below.**)

**Other Supplementary Materials for this manuscript include the following:**

Data S1 to S10 [Namely, Supplementary Table 1 to 11]

Supplementary Table 1. Metabolic genes associated with CRC prognosis (Fig. 1a).

Supplementary Table 2. Prognostic genes only within high-nk infiltration patients (Fig. 1a).

Supplementary Table 3. Differentially expressed genes (Fig. 1a).

Supplementary Table 4. Differentially expressed proteins (Fig. 1a).

Supplementary Table 5. The ranked list of proteins associated with Fig. 1a.

Supplementary Table 6. Gene list of Fig. 1k (NK cell-mediated cytotoxicity and KEGG analysis of global genes).

Supplementary Table 7. Differential nuclear metabolites between ACAT1 NLS and WT in ACAT1-depleted HCT116 cells.

Supplementary Table 8. Nuclear ACAT1 immunoprecipitation mass spectrum analysis (Top 50 candidate proteins).

Supplementary Table 9. Gene list of antigen processing and presentation pathway and NK cell activation in Fig. 4f, g.

Supplementary Table 10. KEGG analysis of genes upregulated in p50 K146Q-expressing HCT116 cells (related to Fig. 4f, g).

Supplementary Table 11. Key resources table.

Materials and Methods

**Supplemental materials and methods**

**Subcutaneous tumor model**

8×10^5^ CT26 cells were subcutaneously injected into six-week-old female BALB/c mice or five-week-old female NSG mice (six mice per group). 1×10^6^ MC38 or 5×10^5^ B16F10 cells were subcutaneously injected into six-week-old female C57BL/6J mice.

For NK cells depletion experiment, 100 μL of polyclonal anti-asialo-GM1 antibody were injected into BALB/c mice via intraperitoneal injection twice a week for total two weeks. Normal rabbit serum was used as control.

Tumor volume was measured every 3-4 days using the formula L×W^2^×0.5 (L, the longest diameter; W, the shortest diameter). At the endpoint, mice were euthanized by cervical dislocation and tumors were dissected for subsequent analysis.

**MTS analysis**

Cell proliferations of MC38 and CT26 cells were evaluated by the MTS Assay Kit (Abcam, ab197010) following the manufacturer’s instructions. Briefly, cells were seeded at a density of 1×10^4^ cells/ml into a 96-well plate, and incubated with MTS reagent for two hours at 37°C on day 1, 2, 3, 4, 5, 6. OD values were measured at 490 nm.

**Immunoprecipitation and immunoblotting analysis**

Extraction of proteins from cultured cells using a modified buffer was followed by immunoblotting or immunoprecipitation with corresponding antibodies, as described previously^1^.

**Immunohistochemistry and immunofluorescence**

For immunohistochemistry staining, tissues were stained with indicated antibodies and DAB.

For immunofluorescence staining, cells or tissues were fixed with 4% paraformaldehyde and incubated with primary antibodies (at a 1:100 or 1:200 dilution), Alexa Fluor dye-conjugated secondary antibodies and DAPI according to standard protocols. Cell imaging was performed using LSM880 with fast airyscan (ZEISS) or SIM. Tissue imaging was performed using KF-PRO-020 (KFBIO) with sCMOS.

For semiquantitative scoring of tissues stained with immunohistochemistry or immunofluorescence, the H-Score method^2^ was used.

**ACAT1 activity assay**

The activity of the ACAT1 was measured as previously described^3^. Briefly, the ACAT1-Flag or its mutant was immunoprecipitated from 5-6×10^6^ HCT116 cells by Anti-Flag Magnetic Beads, and was disassociated from the beads by 3×FLAG peptide. Then, disassociated ACAT1-Flag or its mutant was added to the assay buffer containing 50 mM Tris-HCl (pH 8.0), 20 mM MgCl_2_, 50 µM CoA, 10 µM acetoacetyl-CoA, and 40 mM KCl. The change in absorbance was detected at 303 nm by TECAN Spark 10M.

ACAT1 activity assay was also performed using mitochondria isolated from 1.5×10^7^ HCT116 cells expressing ACAT1-Flag or its mutant, and subsequent procedure was same as the method mentioned above.

**Measurement of nuclear acetyl-CoA concentrations**

Nuclei were isolated by Nuclei PURE Prep kit. Briefly, 3×10^6^ cells washed with PBS were scraped from the dishes and incubated in lysis buffer. Then, cell lysate was placed on top of a 1.8 M sucrose gradient and centrifuged at 30,000 g for 45 min at 4°C. Nuclei were collected and washed with the nucleus storage buffer. The acetyl-CoA concentrations in the isolated nuclei were measured by acetyl-CoA fluorometric assay kit immediately.

**Subcellular fractionation analyses**

For immunoblot analyses, cytosol and mitochondria fractions were isolated using the Qproteome Mitochondria Isolation Kit according to manufacturer’s instructions. Cytoplasm and nuclear protein were separated using Nuclear and Cytoplasmic Protein Extraction Kit according to manufacturer’s instructions.

For IP and DNA pull-down assays, nuclear proteins were extracted as previously described^4^. Briefly, cells were washed twice with ice-cold 1x PBS and lysed in buffer A (10 mM HEPES, pH 7.4, 10 mM KCl, 1.5 mM MgCl_2_, 0.5 mM EDTA, 0.5 mM EGTA) supplemented with fresh protease and phosphatase inhibitor and 0.1% NP-40. The lysates were kept on ice for 15 min and followed by centrifugation to isolate the cytoplasmic fraction. The nuclei pellet was then washed twice in buffer A (without NP-40) and lysed on ice for 30 min in buffer B (10 mM HEPES pH 7.4, 0.42 M NaCl, 2.5% ultra-pure glycerol, 1.5 mM MgCl_2_, 0.5 mM EDTA, 0.5 mM EGTA) supplemented with fresh protease and phosphatase inhibitors and 1mM DTT. The nuclear protein was isolated from supernatant after centrifugation and diluted with 2 volumes PBS or IP lysis buffer for IP and DNA pull-down assays.

**Purification of recombinant proteins**

GST-p50 (1-366aa), its mutant K146R and His-SUMO-ACAT1 were expressed in bacteria and purified. Briefly, the vectors expressing the GST-p50 (1-366aa) or its mutant K146R were used to transformed BL21 (DE3) bacteria. The vectors expressing His-SUMO-ACAT1 were used to transformed Rosetta (DE3) bacteria. Then 0.5 mM isopropyl-beta-D thiogalactopyranoside was added to induce protein expression (16 h, 16°C). After the addition of proteasome inhibitors, cell pellets were sonicated in PBS and centrifuged at 12,000 g for 30 min, 4°C. The cleared lysates were loaded onto glutathione resin (Genescript, L00206) or Ni-NTA resin (Genescript, L00250), and were rotated at 4°C for 4 h. After the extensive wash of beads, proteins were eluted for 1 h in GST elution buffer (50 mM Tris-HCl pH 8.0, 10 mg/ml glutathione, 150 mM NaCl) or His elution buffer (PBS pH 7.4, gradient 20-500 mM imidazole), and then dialyzed against PBS.

**In vitro acetylation assay**

2 µg purified GST-p50 (1-366aa) or its mutant K146R was mixed with 400 ng His-SUMO-ACAT1 in acetylation assay buffer (40 mM Tris-HCl pH 8.0, 75 mM KCl) containing 10 µM acetyl-CoA or not in a final volume of 60 µL for 45 min at 30°C. The reaction was terminated by the addition of SDS-loading buffer and the incubation at 95°C for 10 min. Immunoblot analyses were performed to detect the acetylated proteins.

**GST pull-down assay**

Purified GST-p50 (1-366aa) was incubated with purified His-SUMO-ACAT1 or ACAT1-Flag exogenously expressed in HEK293T cells, and loaded to the glutathione agarose beads (MCE, HY-K0222). The mixture was then rotated at 4°C overnight. After washing the beads five times with washing buffer (50 mM Tris-HCl pH 7.4, 150 mM NaCl, 0.5% Tween-20), the bound proteins were eluted by 1×SDS-loading buffer and performed to immunoblot analyses.

**Biotin DNA pull-down assay**

Streptavidin magnetic beads (MCE, HY-K0208) alone or with 5 µg 5’-biotinylated dsDNA were per-incubated in blocking buffer (25 mM Tris-HCl pH 7.4, 50 mM NaCl, 1 mM DTT, 5% glycerol and 10 mg/ml BSA) for 30 min at room temperature. The nuclear proteins extracted as mentioned above were then loaded to the beads and rotated overnight at 4°C. After washing the beads twice with washing buffer (25 mM Tris-HCl pH 7.4, 50 mM NaCl, 1 mM DTT, and 5% glycerol), the bound proteins were eluted by 1×SDS-loading buffer and performed to immunoblot analyses.

**Duolink in situ proximity ligation assay (PLA)**

PLA was performed with the Duolink in Situ Orange Starter Kit Mouse/Rabbit (Sigma Aldrich, DUO92102). Briefly, after fixation and permeabilization, HCT116 and CT26 cells were incubated with primary antibodies (at a 1:100 dilution), secondary PLA-probes, and DAPI according to the manufacturer’s instruction. The positive signal is captured only when the distance between the two antigens is less than 40 nm. Images of the cells were pictured by Spining Disk Confocal microscopes (Nikon CSU-W1).

**Molecular dynamics simulations**

3GUT, a heterodimer formed by nuclear factor p105 subunit and transcription factor p65, was chosen as the structural template for the human p50 homodimer construction. We replaced p65 in the primary structure of 3GUT into p50, and obtained the human p50 homodimer preserved as WT-Dimer.pdb. Residues K146 on the two chains of the p50 homodimer were then mutated into Q146 preserved as K146Q-Dimer.pdb or added with the acetyl-groups preserved as K146ac-Dimer.pdb. Molecular dynamics simulations of the WT-, K146Q- and K146ac-Dimer were performed to compare the binding differences with DNA. To ensure the integrity of the protein and DNA, 3 nucleotides were retained at the 3 ′ end of the 5 ′ -GGGACTTTCC-3 ′, with the following sequence: 5’-GGGACTTTCCGCT-3’, 3’-CCCTGAAAGGCGA-5’.

Gromacs (2022.3) was used to conduct the molecular dynamics simulations. The prepared protein structure was solvent filled with SPC explicit solvent model, and the system shape was set as cube, the closest distance between the solvent edge and the protein structure was set as 1 nm. To ensure the charge balance of the system, Cl^-^or Na^+^ was added so that the electrostatic charge of the system was 0.

Gradient descend method was used to realize energy minimization with the performance of 50000 steps. The threshold of energy difference between each step was set as 0.01 kJ / mol, and the convergence ended early when the total energy of the system was less than 1000 kJ/mol.

In the meantime, we need to pre-equilibrate the solvent and ions around the protein. First, the temperature was equilibrated under NVT ensemble (canonical ensemble): time 100 ps, time step size 2 fs. The chemical bonds (including hydrogen bonds) were limited by LINCS (LINear Constraint Solver) algorithm, the temperature coupling was conducted by V-rescale algorithm, the temperature of system was set as 300 K. The second stage was conducted in the NPT ensemble (constant-pressure, constant-temperature) to stabilize the pressure and the density of the system: time 100 ps, time step size 2 fs. LINCS algorithm was used to limit the chemical bonds (including hydrogen bonds), and V-rescale algorithm was used for temperature coupling. Isotropic Parrinello-Rahman algorithm was used for pressure coupling.

Finally, the system after being equilibrated was simulated for 200 ns, the time step size was 2.0 fs, the constant temperature was 300 K, the constant-pressure was one atmospheric pressure (atm), the cut-off radius was 1.4 nm. PME (Particle Mesh Ewald) was used to calculate long-range static power, LINCS algorithm was used to limit the hydrogen bonds, V-rescale algorithm was used for temperature coupling and isotropic Parrinello-Rahman algorithm was used for pressure coupling.

**ELISA**

Cells were plated on six-well plate and the supernatant was collected 48 h later. After centrifugation, the concentration of CCL5, CXCL10, CXCL11 in the supernatant was measured by ELISA kits according to manufacturer’s instruction.

**Smart-seq2, RNA seq and data analyses**

For Smart-seq2 (switching mechanism at 5’ end of the RNA transcript), single NK cells were sorted and processed as described in previous study^5^. Briefly, total RNA was extracted and mixed with anchored oligo-dT primer (10 µM, 5′-AAGCAGTGGTATCAACGCAGAGTACT_30_VN-3′, where “N” is any base and “V” is either “A”, “C” or “G”) and dNTP mix (10 mM), denatured at 72 °C for 3 min and placed on ice immediately. Then, 7 µL of the first-strand reaction mix, containing 0.50 µL SuperScript II reverse transcriptase (200 U/µL), 2 µL Superscript II First-Strand Buffer (5×), 0.25 µl RNAse inhibitor (40 U/µL), 0.9 µL MgCl_2_ (100 mM), 0.25 µL DTT (100 mM), 2 µl betaine (5 M), 1 µL TSO (10 µM) and 0.1 µL nuclease-free water were added for reverse transcription. After this, samples were followed by PCR preamplification with the mixture containing: 25 µL KAPA HiFi HotStart ReadyMix, 1 µL ISPCR primers (10 µM, 5′-AAGCAGTGGTATCAACGCAGAGT-3′) and 14 µL nuclease-free water (Gibco). Finally, after tagmentation reaction and PCR amplification, samples were sequenced on Illumina HiSeq 2000.

For RNA-seq, total RNA was extracted with Trizol reagent (Invitrogen) and mRNA was purified from total RNA using poly-T oligo-attached magnetic beads. Sequencing libraries were generated using NEBNext® Ultra^TM^ RNA Library Prep Kit for Illumina® (NEB, USA) following manufacturer’s recommendations and index codes were added to attribute sequences to each sample. High-throughput sequencing was performed on an Illumina Novaseq platform and 150 bp paired-end reads were generated.

We first used Trim Galore (v0.6.10) to trim 3’ end of reads. Resulting paired end cleaned reads were then aligned to the hg38/mm10 reference genome using STAR (2.7.11a). The uniquely mapped reads were assigned to UCSC annotated genes using featureCounts (v2.0.6). DESeq2 was used to performed differential gene expression analysis. Gene set enrichment analysis was performed using R packages fgsea (v1.27.1).

**Quantitative real-time PCR**

Total RNA was extracted with RNA Quick Purification kit (ESscience, RN001) and the reverse transcription was performed with PrimeScript™ RT Master Mix (TAKARA, RR036A) according to manufacturer’s instructions. Quantitative real-time PCR analysis was performed with GoTaq Green Master Mix (Promega, M7122) using a Roche LightCycler96/384. Data were normalized with the expression of a reference gene (β-actin/GAPDH) for each experiment.

**Detection of targeted nuclear metabolites**

Cells were pretreated with culture medium (supplemented with 10% DFBS) lacking sodium pyruvate. Nuclei were then isolated from 4×10^7^ HCT116 cells and thawed on ice, lysed with 500 µL of 80% methanol/water (precooled at -20°C) and vortexed for 2 min under the condition of 2500 r/min. The sample was then frozen in liquid nitrogen for 5 min, removed on ice for 5 min, after that, the sample was vortexed for 2 min (repeated for 3 times). The sample was centrifuged at 12000 rpm for 10 min at 4°C, the supernatant was transferred to a new centrifuge tube and placed in -20°C refrigerator for 30 min. Then, the supernatant was centrifuged at 12000 rpm for 10 min at 4°C. After centrifugation, transfer 200 μL of supernatant through Protein Precipitation Plate for further LC-MS/MS analysis.

All those metobolites were detected by MetWare (http://www.metware.cn/) based on the AB Sciex QTRAP 6500 LC-MS/MS platform.

**Mass spectrometry analysis**

ACAT1-Flag proteins or HA-p50 (1-366aa) were immunoprecipitated from HCT116 cells. The bound proteins were disassociated from beads with the incubation at 95°C for 10 min. And the factions were processed as previously described^6^. Peptides were dissolved in 0.1% FA (Formic acid) and 2% ACN (Acetonitrile), then directly loaded onto a reversed-phase analytical column (75 µm i.d.×150 mm, packed with Acclaim PepMap RSLC C18, 2 µm, 100 Å, nano-Viper). The gradient was comprised of solvent B (0.1% FA in 80% ACN) ranging from 5% to 50% with in 40 min, and climbing to 90% in 5 min, then holding at 90% for the 5 min. All at a constant flow rate of 300 nL/min.

The MS analysis was performed on Q Exactive hybrid quadrupole-Orbitrap mass spectrometer (ThermoFisher Scientific). The peptides were subjected to NSI source followed by tandem mass spectrometry (MS/MS) in Q ExactiveTM (Thermo) coupling with the UPLC. Intact peptides were detected in the Orbitrap at a resolution of 70,000. Peptides were selected for MS/MS using NCE setting as 27; ion fragments were detected in the Orbitrap at a resolution of 17,500. A data-dependent procedure that alternated between one MS scan followed by twenty MS/MS scans was applied for the top 20 precursor ions which threshold value were 1E4, and the dynamic exclusion time was 30.0 s. The electrospray voltage was 2.0 kV. AGC (Automatic gain control) was used to prevent overfilling of the ion trap; 1E5 ions were accumulated for generation of MS/MS spectra. For MS scans, the m/z scan range was 350 to 1800 m/z. Fixed first mass was set as 100 m/z.

Protein identification was performed with MASCOT (http://[www.matrixscience](http://www.matrixscience). com/) software by searching Uniprot database.

**Transwell migration assay**

NK cells were separated from spleens of BALB/c mice and C57BL/6J mice with EasySep Mouse NK Cell Isolation Kit (Stemcell, 19855) and the purity (>80%) was checked on BECKMAN COULTER CytoFLEX LX cell analyzer. The separated NK cells were cultured in MEMα (Gibco) and supplemented with 0.1 mM β-mercaptoethanol, 0.2 mM inositol, 0.02 mM folic acid, 100- 200 U/mL recombinant IL-2 and IL15, 10% horse serum, 10% FBS and 1% penicillin-streptomycin.

CT26 or MC38 cells were seeded at 1×10^5^ cell/well in 24 well plate in 600 µL culture medium. Two days later 1.5×10^5^ NK cells pre-stained with CFSE were added to the upper chamber (5 µm pore size), and the plates were incubated for 6 h at 37°C. Then, the medium in lower chamber were collected and NK cells number was determined by flow cytometry (counting beads were used to calculate absolute number of NK cells).

**References**

1. Zhang, Y. et al. Macrophage-Associated PGK1 Phosphorylation Promotes Aerobic Glycolysis and Tumorigenesis. *Mol Cell* **71**, 201-215 e207 (2018).

2. Zhang, Y. et al. Cul4A-DDB1-mediated monoubiquitination of phosphoglycerate dehydrogenase promotes colorectal cancer metastasis via increased S-adenosylmethionine. *J Clin Invest* **131** (2021).

3. Haapalainen, A.M. et al. Crystallographic and kinetic studies of human mitochondrial acetoacetyl-CoA thiolase: the importance of potassium and chloride ions for its structure and function. *Biochemistry* **46**, 4305-4321 (2007).

4. Sivanand, S. et al. Nuclear Acetyl-CoA Production by ACLY Promotes Homologous Recombination. *Mol Cell* **67**, 252-265 e256 (2017).

5. Picelli, S. et al. Smart-seq2 for sensitive full-length transcriptome profiling in single cells. *Nat Methods* **10**, 1096-1098 (2013).

6. Wang, X. et al. UDP-glucose accelerates SNAI1 mRNA decay and impairs lung cancer metastasis. *Nature* **571**, 127-131 (2019).


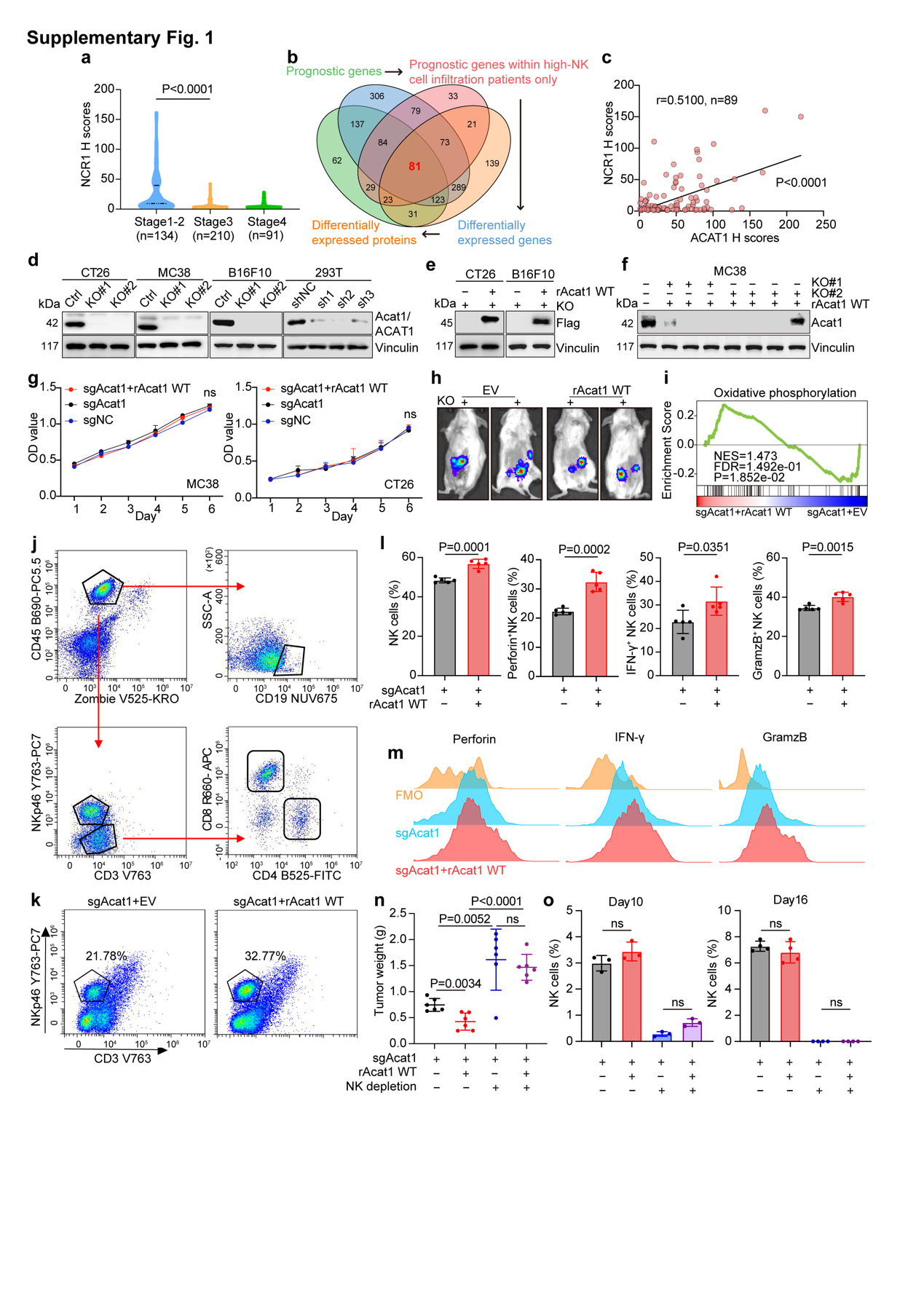


Supplementary Fig. 1: Related to Fig. 1

**ACAT1 promotes cytotoxic NK cell infiltration to suppress CRC growth. a,** The correlation between NCR1 level and tumor stage was determined by IHC staining. **b,** Veen plot showing the filtering rules for proteins positively correlated with NK cell infiltration in CRC. **c,** Immunohistochemistry (IHC) analysis of 355 samples from patients with colorectal cancer was performed and the correlation between ACAT1 and NCR1 levels was analyzed in stage1-2 tumor tissues. **d,** CT26, MC38 and B16F10 cells were infected with lentivirus for knockout (KO) of endogenous Acat1. The selected single clones of KO cells were evaluated via immunoblotting. HEK293T cells were infected with lentivirus expressing shACAT1 to knock down endogenous ACAT1 and subjected to immunoblot analysis. **e,** Acat1-KO CT26 and B16F10 cells rescued with Acat1-Flag WT were subjected to immunoblot analysis before injection. **f,** Acat1-KO MC38 cells were infected with different concentrations of rAcat1 WT lentivirus to ensure the expression level of rAcat1 was equivalent to endogenous Acat1 in wild type MC38 cells. **g,** MTS analysis of MC38 and CT26 cells. **h,** Bioluminescence imaging of mice in (**Fig. 1i**) were shown. **i,** GSEA of oxidative phosphorylation signaling genes based on Smart-seq2 data. **j,** Gating strategy for the detection of mouse CD4^+^ T cells, CD8^+^ T cells, CD19^+^ B cells and NK (NKp46^+^) cells. **k,** Representative plots showing the expression of NKp46 and CD3 in tumor-infiltrating CD45^+^ immune cells from the BALB/c mice described in (**Fig. 1n**). **l,m,** The percentages of tumor-infiltrating NK cells and Perforin^+^, IFN-γ^+^, GzmB^+^ NK cells (5 mice per group) related to **Fig. 1n,o** (**l**); the histograms including FMO (Fluorescence Minus One) were shown below (**m**). **n,** Tumor weight of mice described in (**Fig. 1r**). **o,** The percentage of peripheral blood NK cells (CD45^+^CD3^-^NKp46^+^) was calculated to determine the efficiency of NK cell depletion with an anti-ASGM1 antibody in the BALB/c mice described in (**Fig. 1r**). The data are shown as the means ± SDs. Unpaired two-tailed t test (**a, l**), pearson correlation test (**c**), one-way ANOVA (**n, o**), two-way ANOVA (**g**). Immunoblots representative of three independent experiments are shown.


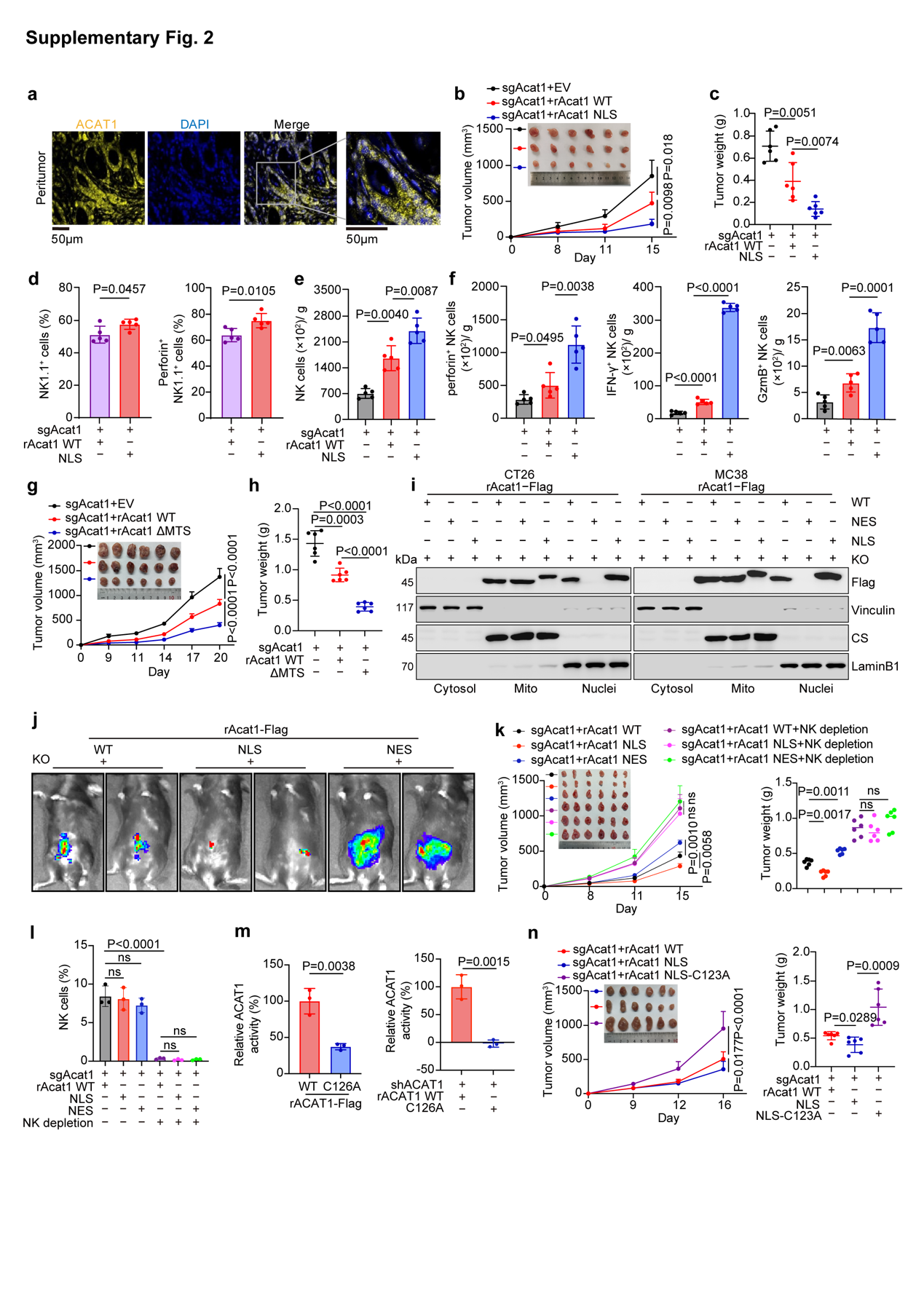


Supplementary Fig. 2: Related to Fig. 2

**Nuclear ACAT1 engages activated NK cells to accumulate in the TME. a,** Immunofluorescence staining with anti-ACAT1 antibody was performed in peritumoral tissues from patients with colorectal cancer. Representative images of IF staining are shown. **b,c** Tumor growth (**b**) and tumor weight (**c**) of CT26 cells subcutaneously injected into BALB/c mice (6 mice per group). **d-f,** Acat1-KO MC38/CT26 cells rescued with indicated mutants were subcutaneously injected into C57BL/6J or BALB/c mice (5 mice per group). Percentages of tumor-infiltrating NK1.1^+^ and Perforin^+^ NK1.1^+^ cells in C57BL/6J (**d**) or tumor-infiltrating NK cells and Perforin^+^, IFN-γ^+^, GzmB^+^ NK cells in BALB/c (**e, f**) mice were calculated by flow cytometric analysis. **g,h,** Acat1-KO CT26 cells rescued with Acat1-Flag WT or ΔMTS were subcutaneously injected into BALB/c mice (6 mice per group), tumor growth (**g**) and tumor weight (**h**) were measured. **i,** Subcellular fractionation assay of Acat1-KO CT26 and MC38 cells rescued with Acat1-Flag WT, NES or NLS. **j,** Representative images of tumor growth in (**Fig. 2f**) are shown. **k-m,** Acat1-KO CT26 cells rescued with indicated mutants were subcutaneously injected into BALB/c mice (6 mice per group) and then treated with rabbit serum or anti-ASGM1antibody. Tumor volume (**k,** left) and tumor weight (**k,** right) were measured. The percentage of peripheral blood NK cells (CD45^+^CD3^-^NKp46^+^) was calculated to determine the efficiency of NK cell depletion at day 15 (**l**). **m,** ACAT1 activity assay with purified Flag-ACAT1 WT and C126A in vitro (**m,** left), n = 3 biological replicates; ACAT1 activity assay using mitochondria lysate from ACAT1-depleted HCT116 cells rescued with indicated mutants was performed (**m,** right). n = 3 biological replicates. **n,** Acat1-KO CT26 cells rescued with Acat1-Flag WT, NLS or NLS-C123A were subcutaneously injected into BALB/c mice (6 mice per group). Tumor growth (left) and tumor weight (right) were measured. The data are shown as the means ± SDs. One-way ANOVA (**c, e, f, h, l**; tumor weight in **k** and **n**), two-way ANOVA (**b, g,** tumor growth in **k** and **n**) or unpaired two-tailed t test (**d, m**). Immunoblots representative of three independent experiments are shown.


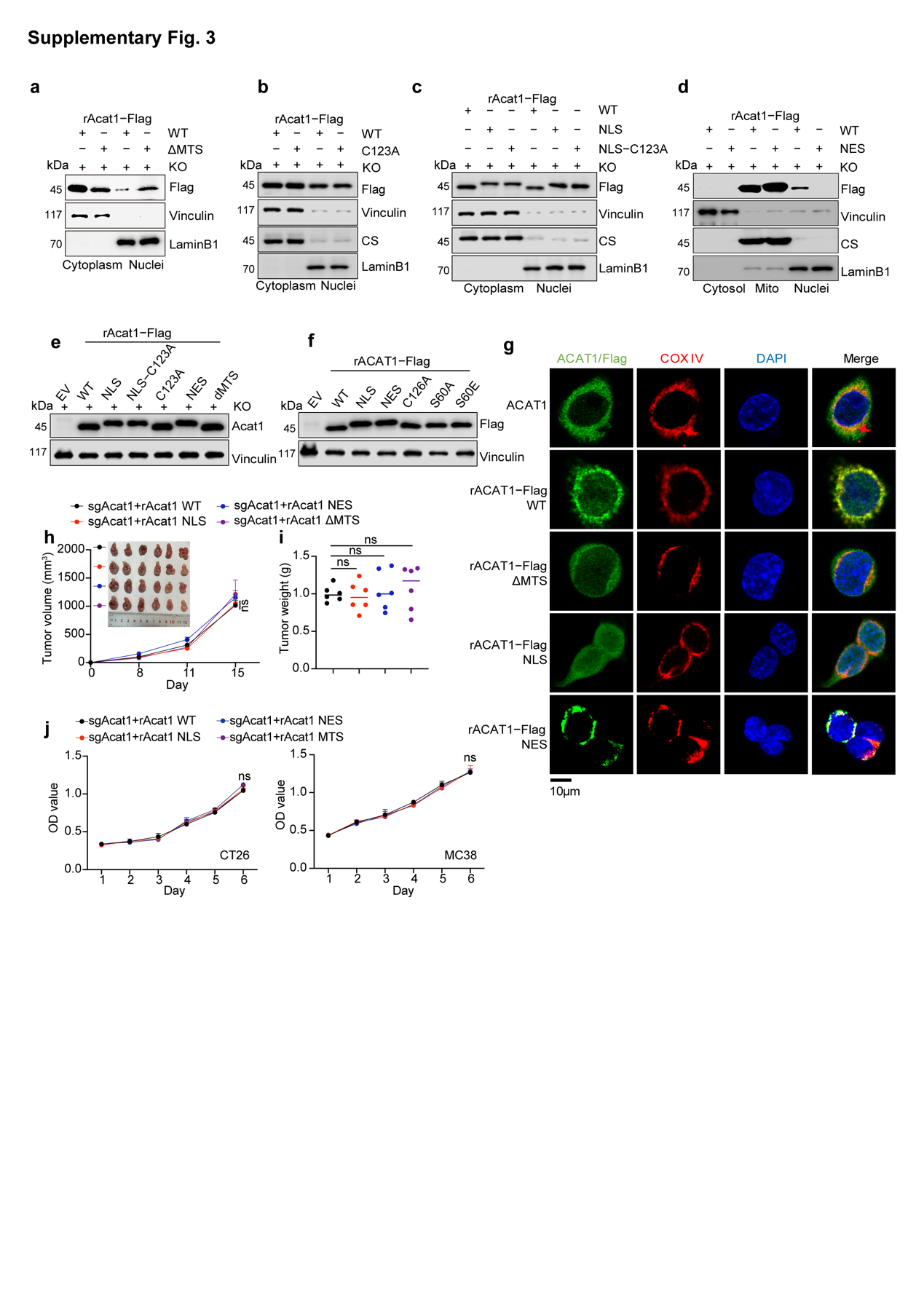


Supplementary Fig. 3: Related to Fig. 2

**Quality control of ACAT1-Flag reconstitution with different mutants. a-d**, Subcellular fractionation assay was performed with Acat1-KO CT26 cells rescued with Acat1-Flag WT or the indicated mutants. Immunoblot analysis was performed with the indicated antibodies (mito, mitochondrial). **e,f,** Acat1-KO CT26 cells and HCT116 cells were infected with lentiviruses expressing different ACAT1-Flag mutants. Immunoblot analysis was performed to test the transfection efficiency. **g,** Representative images of immunofluorescence staining for endogenous ACAT1, exogenous recombinant ACAT1-Flag WT and the indicated mutants in HCT116 cells with anti-ACAT1, anti-Flag, and anti-COX IV antibodies. **h,i,** Acat1-KO CT26 cells rescued with indicated mutants were subcutaneously injected into NSG mice (6 mice per group), tumor volume (**h**) and tumor weight (**i**) were measured. **j,** MTS analysis of CT26 and MC38 cells. The data are shown as the means ± SDs. One-way ANOVA (**i**), two-way ANOVA (**h, j**) Immunoblots representative of three independent experiments are shown.


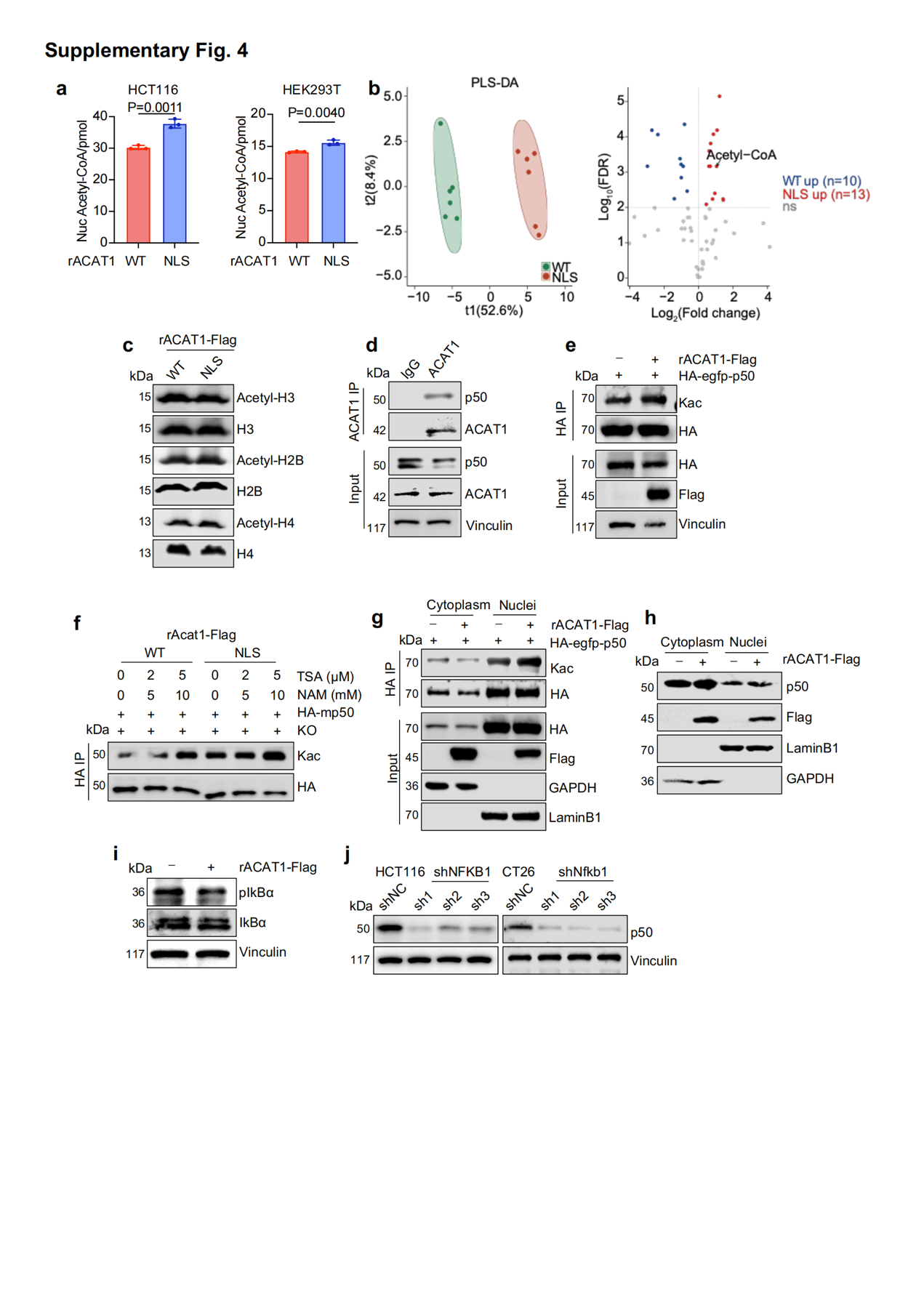


Supplementary Fig. 4: Related to Fig. 3

**Nuclear ACAT1 directly acetylates p50 at K146. a,** Nuclei were isolated from HCT116 (left) or HEK293T (right) cells expressing ACAT1-Flag WT or NLS, and acetyl-CoA was then detected with an acetyl-CoA fluorometric assay kit (nuc, nuclear). **b,** The significant differential nuclear metabolites between ACAT1-depleted HCT116 cells re-expressing ACAT1 WT and NLS (FDR<0.01 and VIP>1). **c,** Immunoblot analysis was performed with the indicated antibodies in HCT116 cells expressing ACAT1-Flag WT or NLS. **d,** Endogenous ACAT1 binding with p50 in HEK293T cells was detected by immunoblot analysis. **e-g,** Total lysates of HEK293T (**e**) and CT26 (**f**) cells or subcellular fractions of HEK293T cells (**g**) transfected with the indicated plasmids were subjected to IP. CT26 cells were treated with or without the indicated concentrations of trichostatin A (TSA) and nicotinamide (NAM) for 12 h. Immunoblot analysis was performed with the indicated antibodies. **h,** Subcellular fractionation assay was performed to detect the cellular distribution of p50 in HCT116 cells transfected with EV or ACAT1-Flag. **i,** Immunoblot analysis was performed in HCT116 cells expressing EV or ACAT1-Flag to measure the levels of pIkBα and IkBα. **j,** The levels of endogenous p50 were measured after HCT116 cells (left) or CT26 cells (right) infected with lentivirus expressing shNFKB1 (left) or shNfkb1 (right). Immunoblots representative of three independent experiments are shown.


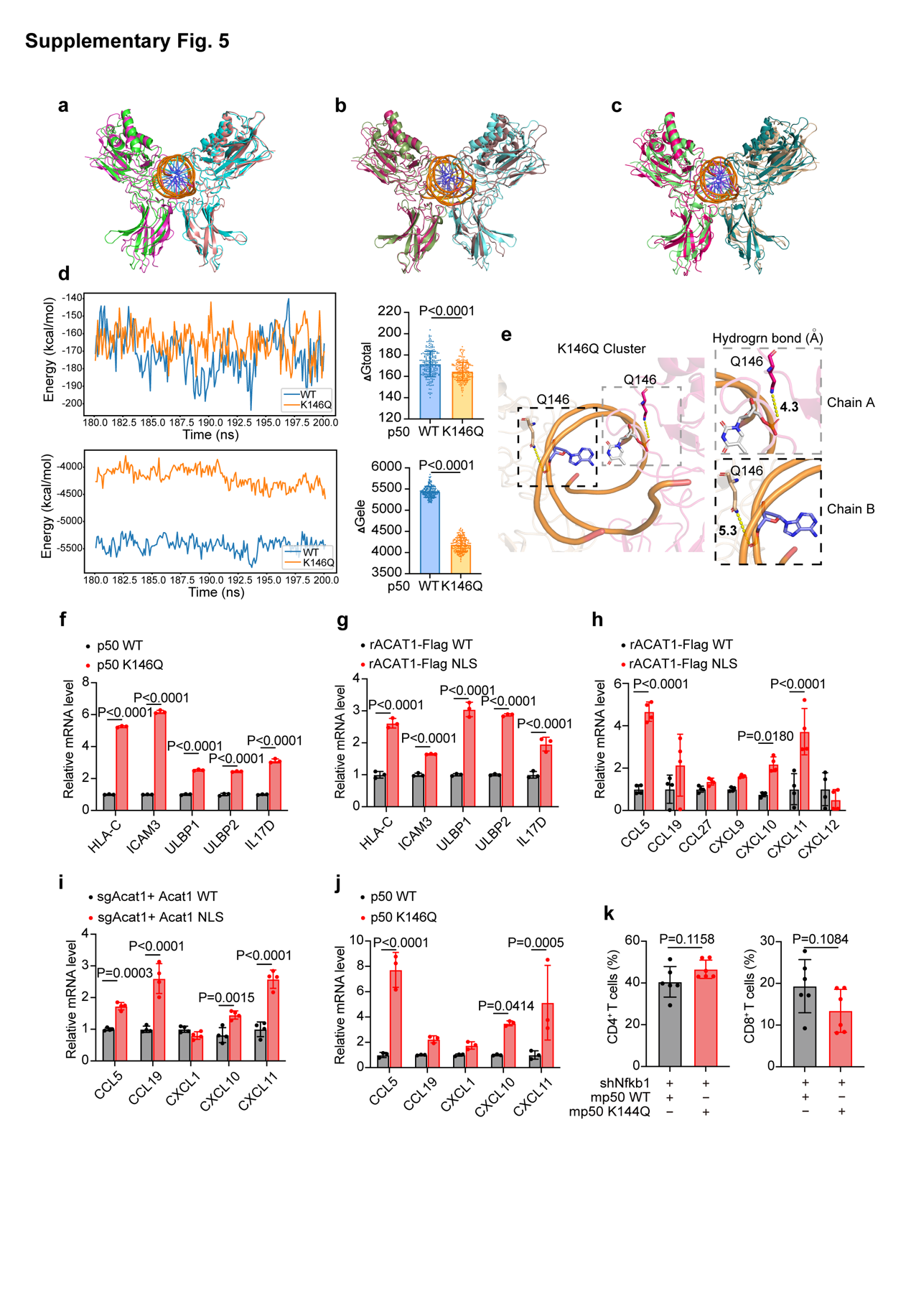


Supplementary Fig. 5: Related to Fig. 4

**p50 K146 acetylation weakens DNA binding ability and thus** **promotes NK cell activation and infiltration. a-c,** Overlapping structures of WT-Dimer and WT-Cluster (**a**)**,** K146Ac-Dimer and K146Ac-Cluster (**b**), and K146Q-Dimer and K146Q-Cluster (**c**). **d,e,** Molecular dynamics simulations of p50 homodimers bound to DNA. The total binding free energy (Gtotal, top) and electrostatic energy (Gele, bottom) were compared between p50 WT and K146Q (paired t test) (**d**). The potential hydrogen bonds (yellow) in K146Q-Cluster are shown. p50 homodimers in K146Q-Cluster are shown as cartoons, DNA is represented by an orange double helix, and the nucleotides bound to p50 are shown as sticks. Q146 is colored dark magenta (chain A) or wheat (chain B) in K146Q-Cluster and shown as sticks (**e**). **f,g,** qPCR analysis of gene expression based on RNA-seq data in HCT116 cells transfected with indicated plasmids. **h-j,** qPCR analysis of chemokines expressed in HCT116 cells (**h, j**) and CT26 cells (**i**) transfected with the indicated plasmids. **k,** Flow cytometric analysis showing the percentage of tumor-infiltrating CD4^+^, CD8^+^ T cells from mice in **(Fig. 4j)**. The data are shown as the means ± SDs. Paired t test (**d**), two-way ANOVA (**f-j**), or unpaired two-tailed t test (**k**).


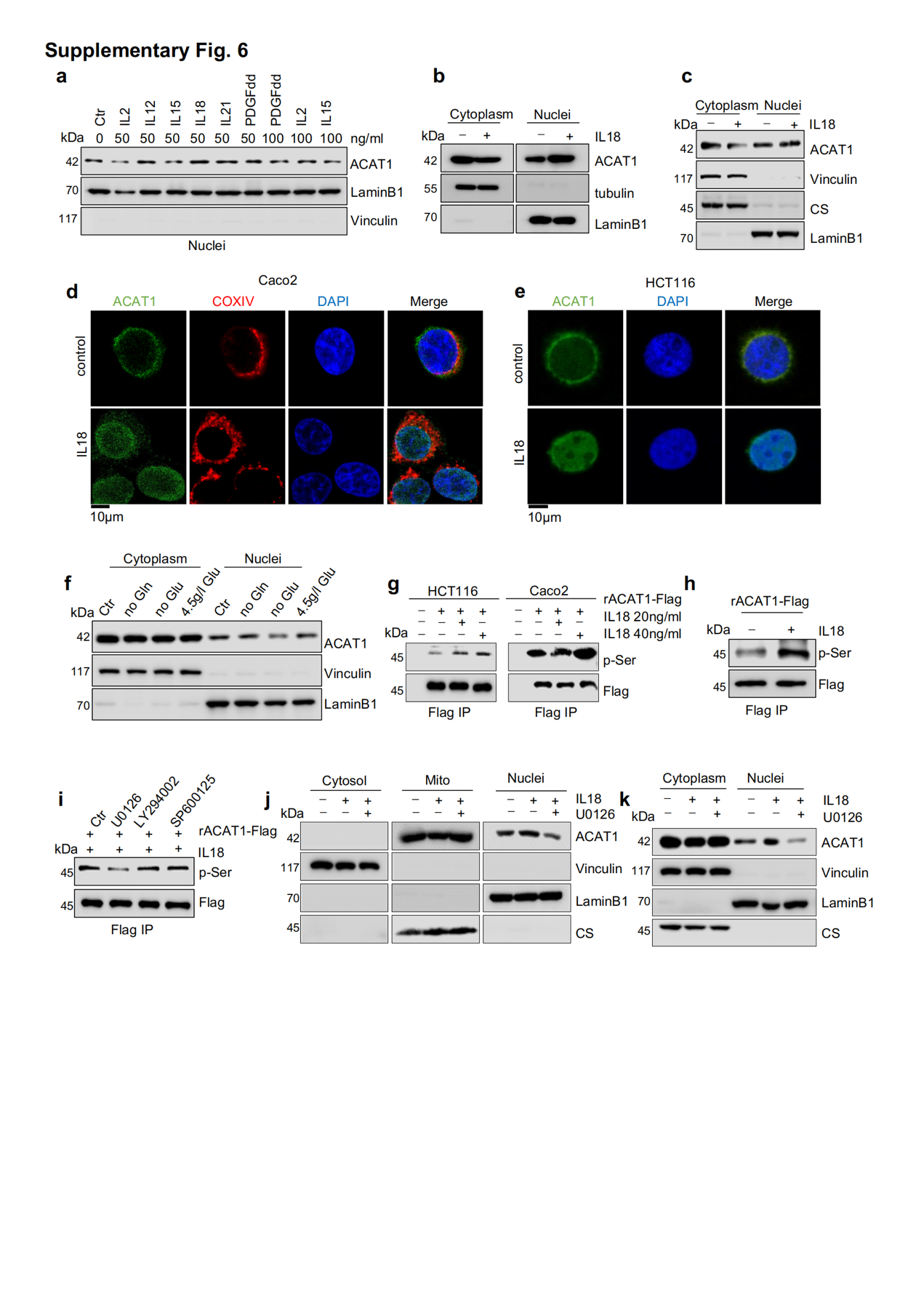


Supplementary Fig. 6: Related to Fig. 5

**The phosphorylation of ACAT1 facilitates its nuclear translocation. a,** HCT116 cells were treated with different cytokines for 12 h, and subcellular fractionation assay was performed to detect nuclear ACAT1. **b,c,** Subcellular fractionation assay was performed with RKO cells (**b**) and Caco2 cells (**c**) treated with or without 50 ng/ml IL18 for 12 h. Immunoblot analysis was performed with the indicated antibodies. **d,e,** Immunofluorescence staining with anti-ACAT1 and anti-COX IV (mitochondrial marker) antibodies was performed in Caco2 cells (**d**) and HCT116 cells (**e**) treated with or without 50 ng/ml IL18 for 12 h. Representative images are shown. **f,** Subcellular fractionation assay was performed with HCT116 cells cultured in RPMI-1640 medium supplemented with 10% DFBS, or under the conditions of no glutamine (Gln), no glucose (Glu), 4.5 g/L Glu for 12 h. Immunoblot analysis was performed with the indicated antibodies. **g-i,** HCT116 cells, Caco2 cells, and RKO cells stably expressing ACAT1-Flag were treated with or without IL18 (**g** and **h**), and HCT116 cells stably expressing ACAT1-Flag were pretreated with 10 μM U0126, 10 μM LY294002, 10 μM SP600125 or not for 4 h before IL18 treatment (**i**). ACAT1-Flag was immunoprecipitated, and immunoblot analysis was performed to detect the phosphorylation level of ACAT1-Flag. **j,k,** Subcellular fractionation assay was performed with HCT116 (**j**) and RKO (**k**) cells treated with or without IL18, or pretreated with U0126 before IL18 treatment. Immunoblot analysis was performed with the indicated antibodies (CS, citrate synthase, a mitochondrial marker). Immunoblots representative of three independent experiments are shown.


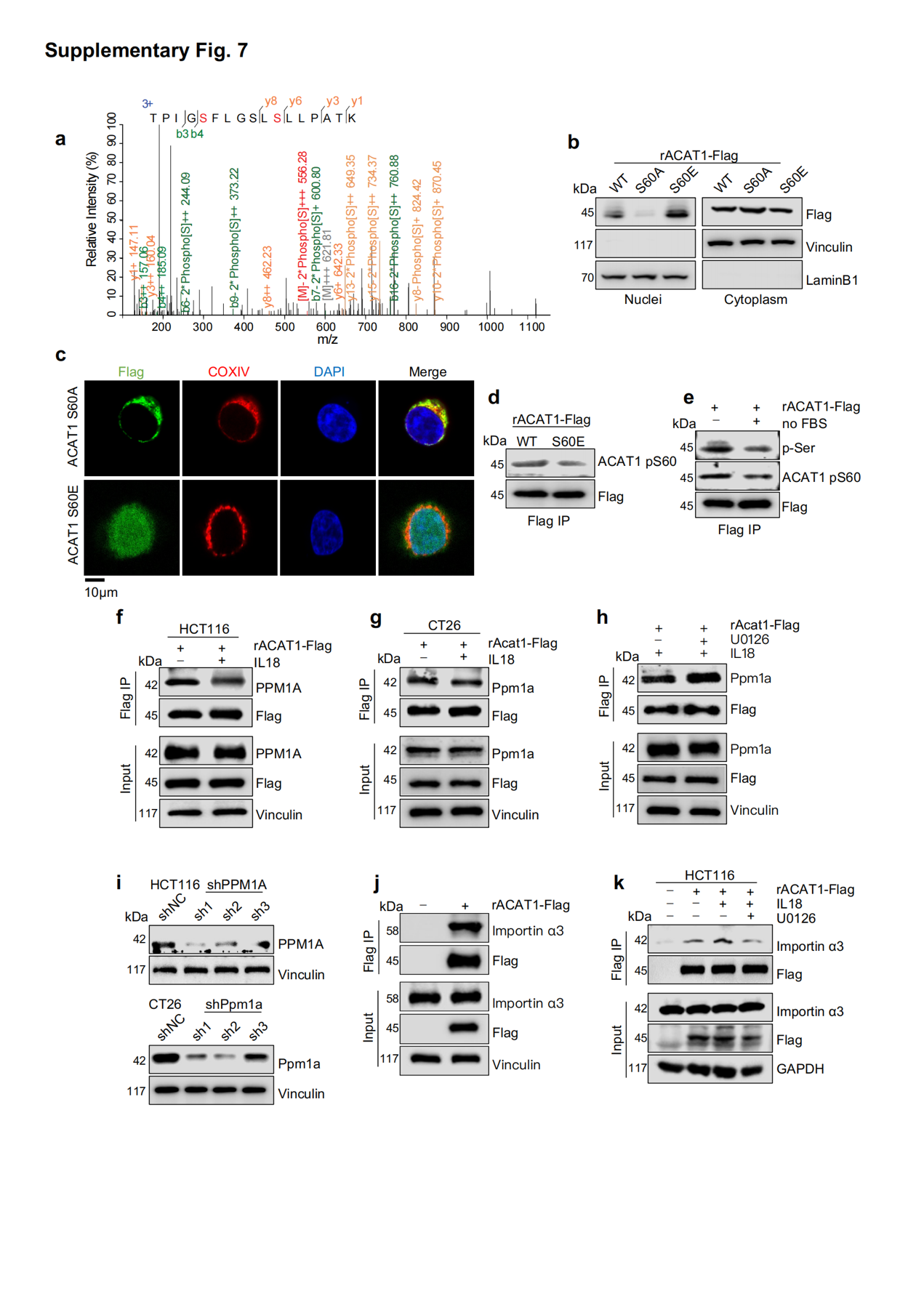


Supplementary Fig. 7: Related to Fig. 5

**PPM1A regulates the nuclear translocation of ACAT1 via importin α3. a,** Subcellular fractionation assay and immunoprecipitation were performed to pull down nuclear ACAT1-Flag from HCT116 cells expressing exogenous ACAT1. S54 and S60 were found to be phosphorylated in the nucleus via mass spectrometry analysis. **b,** Subcellular fractionation assay was performed with HCT116 cells transfected with ACAT1-Flag WT, S60A, or S60E. **c,** Representative images of immunofluorescence staining in HCT116 cells transfected with ACAT1-Flag S60A or S60E. **d,** The specificity of the anti-ACAT1 pS60 antibody was tested. **e,** HCT116 cells expressing ACAT1-Flag were treated with RPMI-1640 medium supplemented with or without 10% FBS, and immunoblot analysis was then performed. **f-h,** ACAT1-Flag/Acat1-Flag was immunoprecipitated from HCT116 cells (**f**) and CT26 cells (**g** and **h**) treated as indicated to detect the interaction with PPM1A/Ppm1a. **i,** The levels of endogenous PPM1A/Ppm1a were detected after HCT116 cells (top) or CT26 cells (bottom) were infected with lentivirus expressing shPPM1A (top) or shPpm1a (bottom). **j,k,** ACAT1-Flag was immunoprecipitated from HEK293T (**j**) and HCT116 (**k**) cells treated as indicated condition to detect the interaction between ACAT1 and importin α3. Immunoblots representative of three independent experiments are shown.


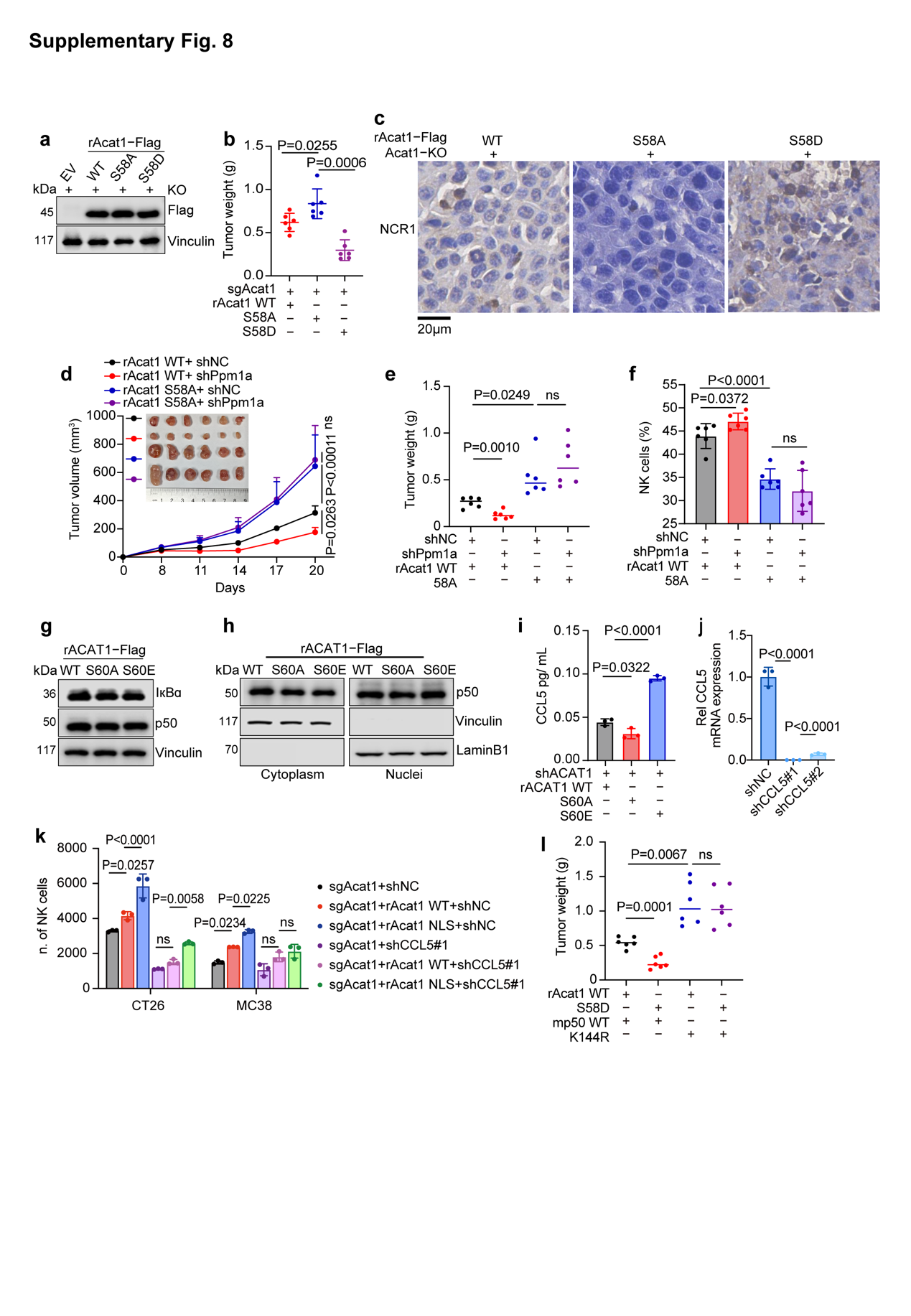


Supplementary Fig. 8: Related to Fig. 6

**ACAT1 pS60 promotes NK cell activation and recruitment. a,** Immunoblot analysis of Acat1-KO CT26 cells rescued with Acat1-Flag WT, S58A, or S58D. **b,** Tumor weight was calculated for the mice described in (**Fig. 6a**). **c,** Representative images of IHC staining with anti-NCR1 antibody in the tumors described in (**Fig. 6a**). **d-f,** Acat1-KO CT26 cells rescued with Acat1-Flag WT or S58A were coinfected with lentivirus expressing shNC or shPpm1a and subcutaneously injected into BALB/c mice (6 mice per group). Tumor growth (**d**) and tumor weight (**e**) were measured. Flow cytometric analysis was performed 13 days after injection to calculate the percentage of tumor-infiltrating NK cells (**f**). **g,** Immunoblot analysis of HCT116 cells transfected with ACAT1-Flag WT, S60A, or S60E. **h,** Subcellular fractionation assay was performed with HCT116 cells transfected with ACAT1-Flag WT, S60A, or S60E followed immunoblot analyses with indicated antibodies. **i,** ACAT1-depleted HCT116 cells rescued with EV, ACAT1-Flag WT or S60E were cultured for 48 h, after which the supernatants were collected. ELISAs were performed to quantify the secretion of CCL5. n = 3 biological replicates. **j,** qPCR analysis evaluating the efficiency of shCCL5#1 and #2 in CT26 cells. **k,** Acat1-KO CT26/MC38 cells re-expressing rAcat1 WT/NLS/NES were infected with shNC or shCCL5#1, and the number of NK cells migrating towards these infected cells was analyzed by flow cytometry. **l,** Tumor weight was calculated for the mice described in (**Fig. 6j**). Immunoblots representative of three independent experiments are shown. The data are shown as the means ± SDs. One-way ANOVA (**b, e, f, i** and **j**) or two-way ANOVA (**d,** **k**).
