# Supplementary figures and images for: Nuclear mitochondrial acetyl-CoA acetyltransferase 1 orchestrates natural killer cell-dependent antitumor immunity in colorectal cancer
Source: Signal Transduct Target Ther. 2025 Apr 28;10:138. doi: 10.1038/s41392-025-02221-y (PMC12034769; doi:10.1038/s41392-025-02221-y)

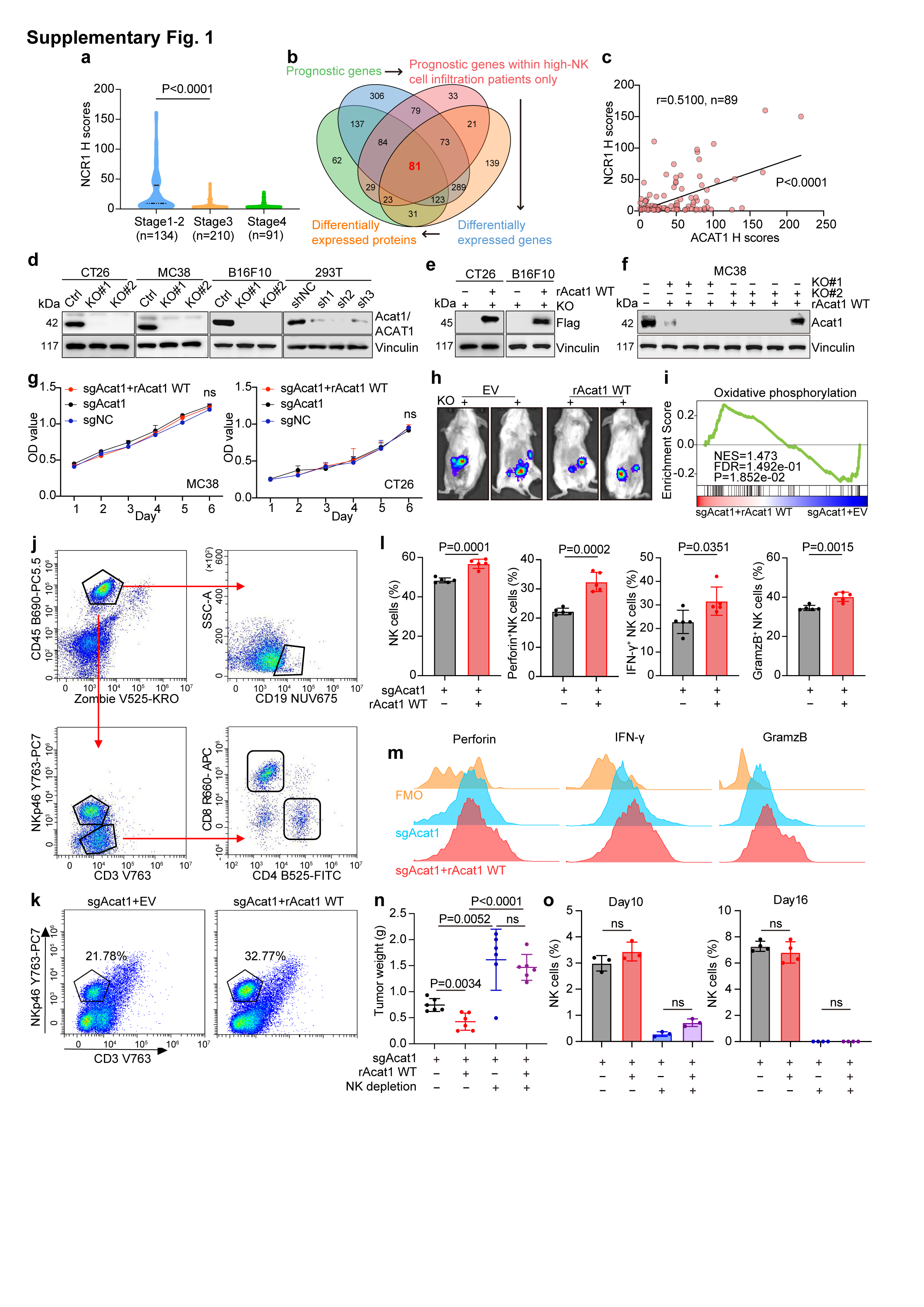

Supplement: Supplementary file 3 — Supplementary Fig.1 [file 41392_2025_2221_MOESM3_ESM.tif]

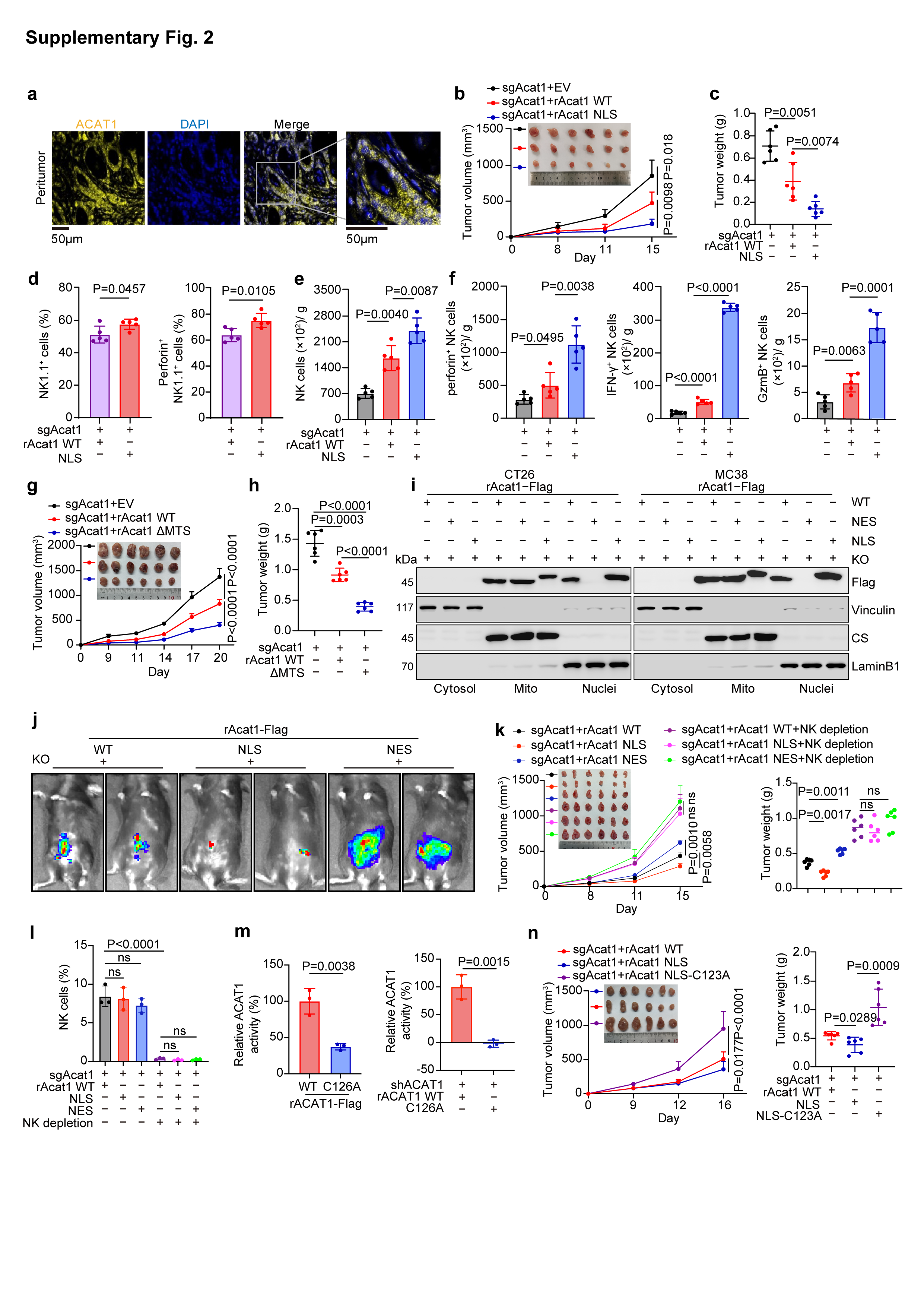

Supplement: Supplementary file 4 — Supplementary Fig.2 [file 41392_2025_2221_MOESM4_ESM.tif]

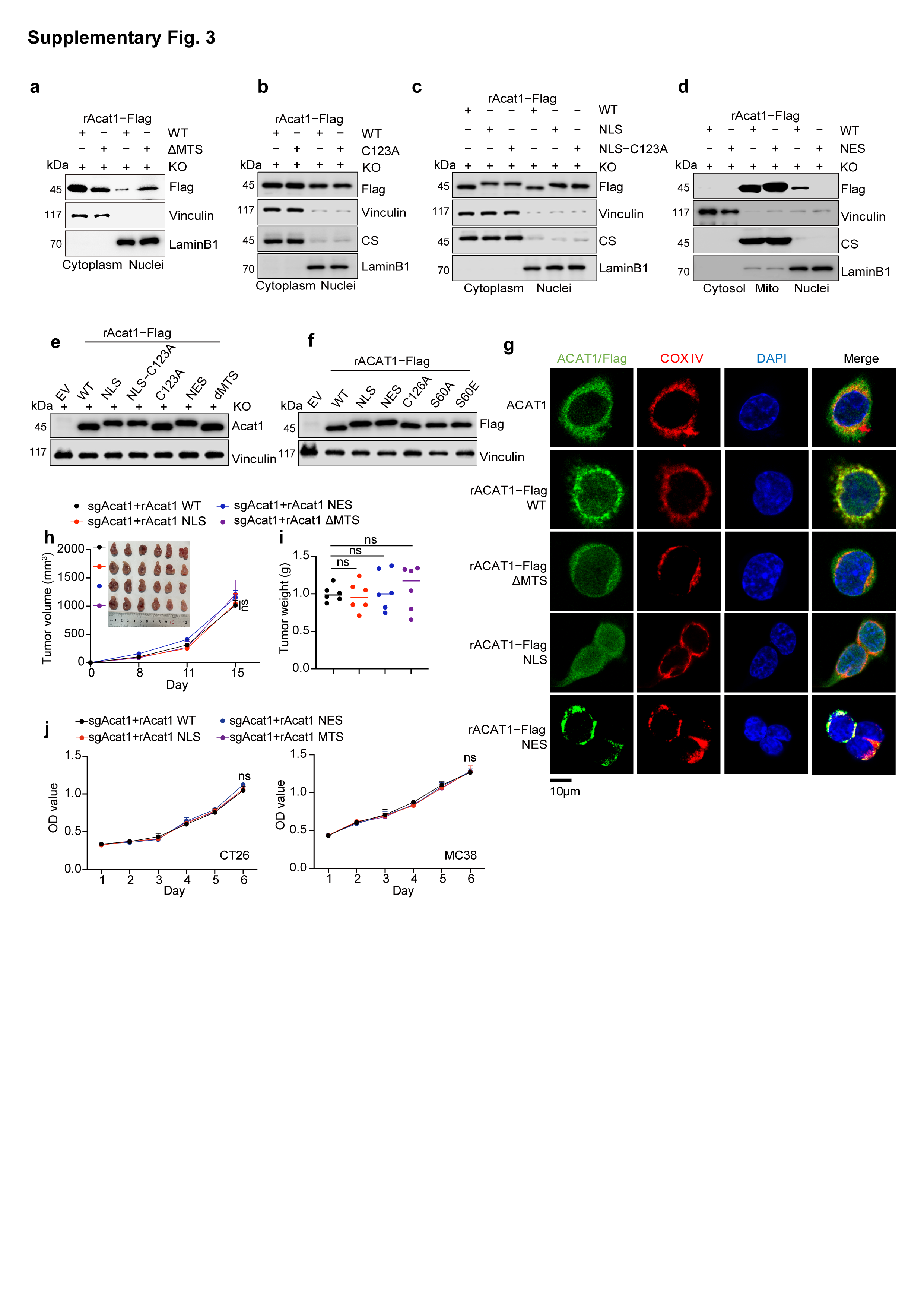

Supplement: Supplementary file 5 — Supplementary Fig.3 [file 41392_2025_2221_MOESM5_ESM.tif]

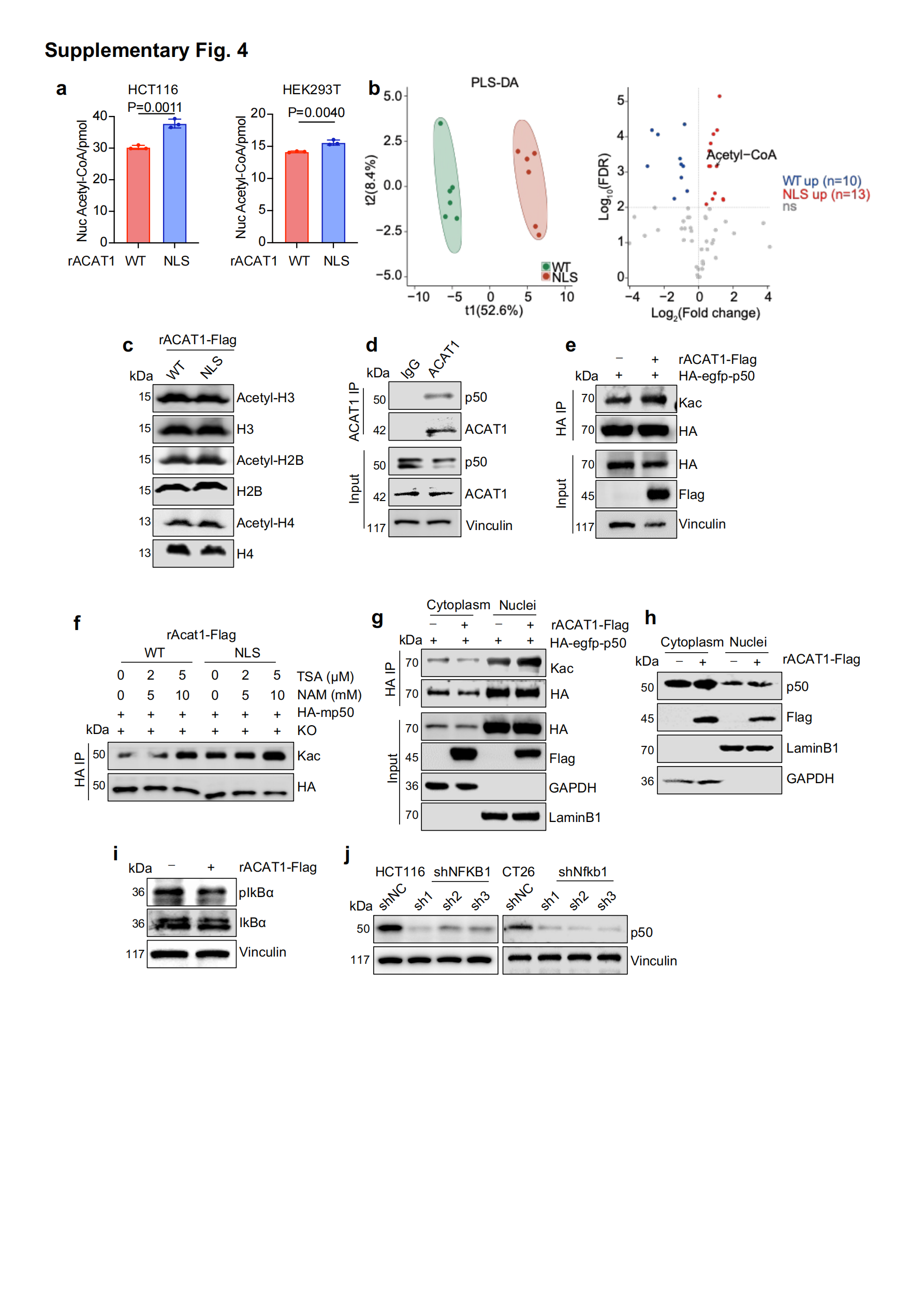

Supplement: Supplementary file 6 — Supplementary Fig.4 [file 41392_2025_2221_MOESM6_ESM.tif]

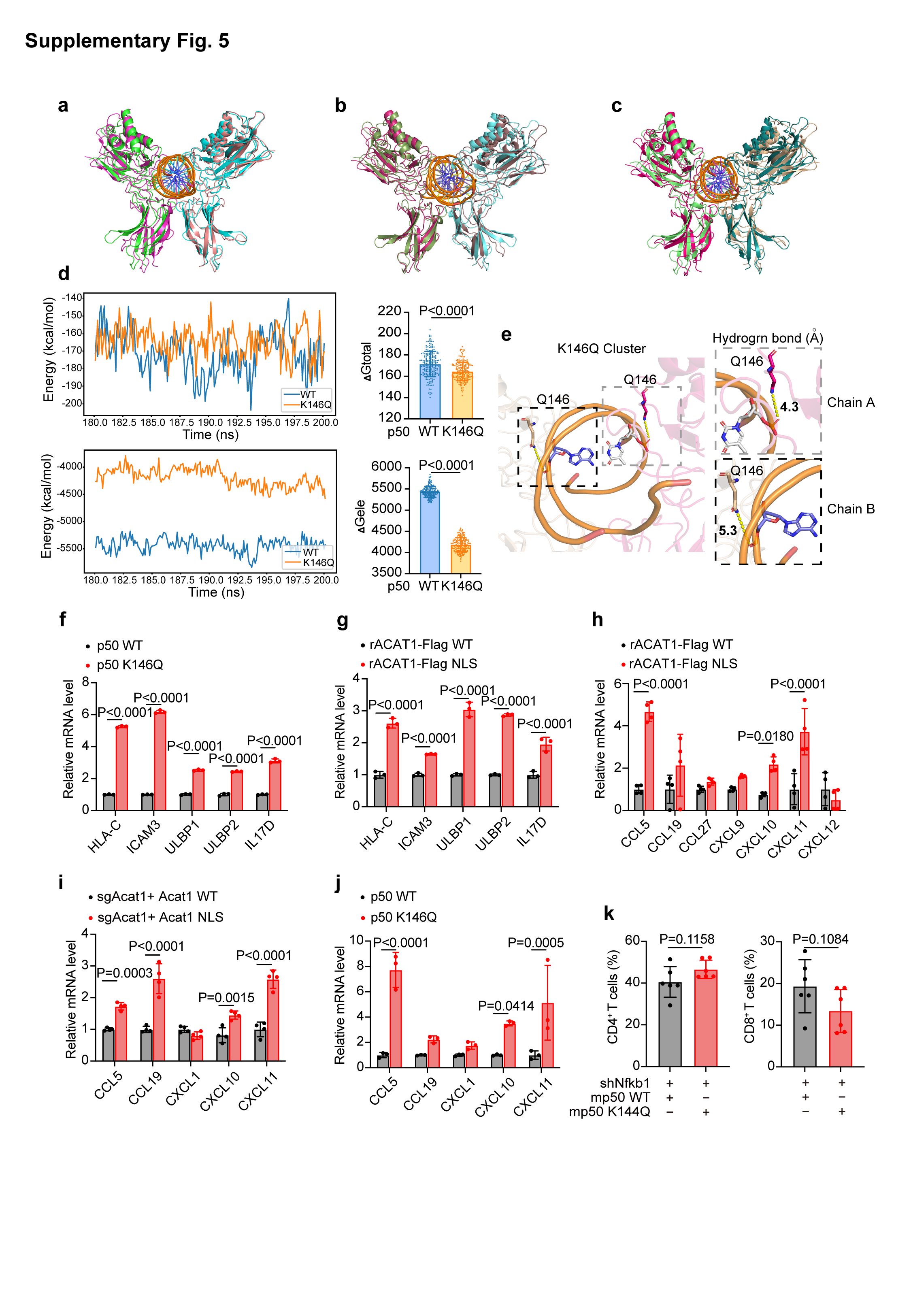

Supplement: Supplementary file 7 — Supplementary Fig.5 [file 41392_2025_2221_MOESM7_ESM.tif]

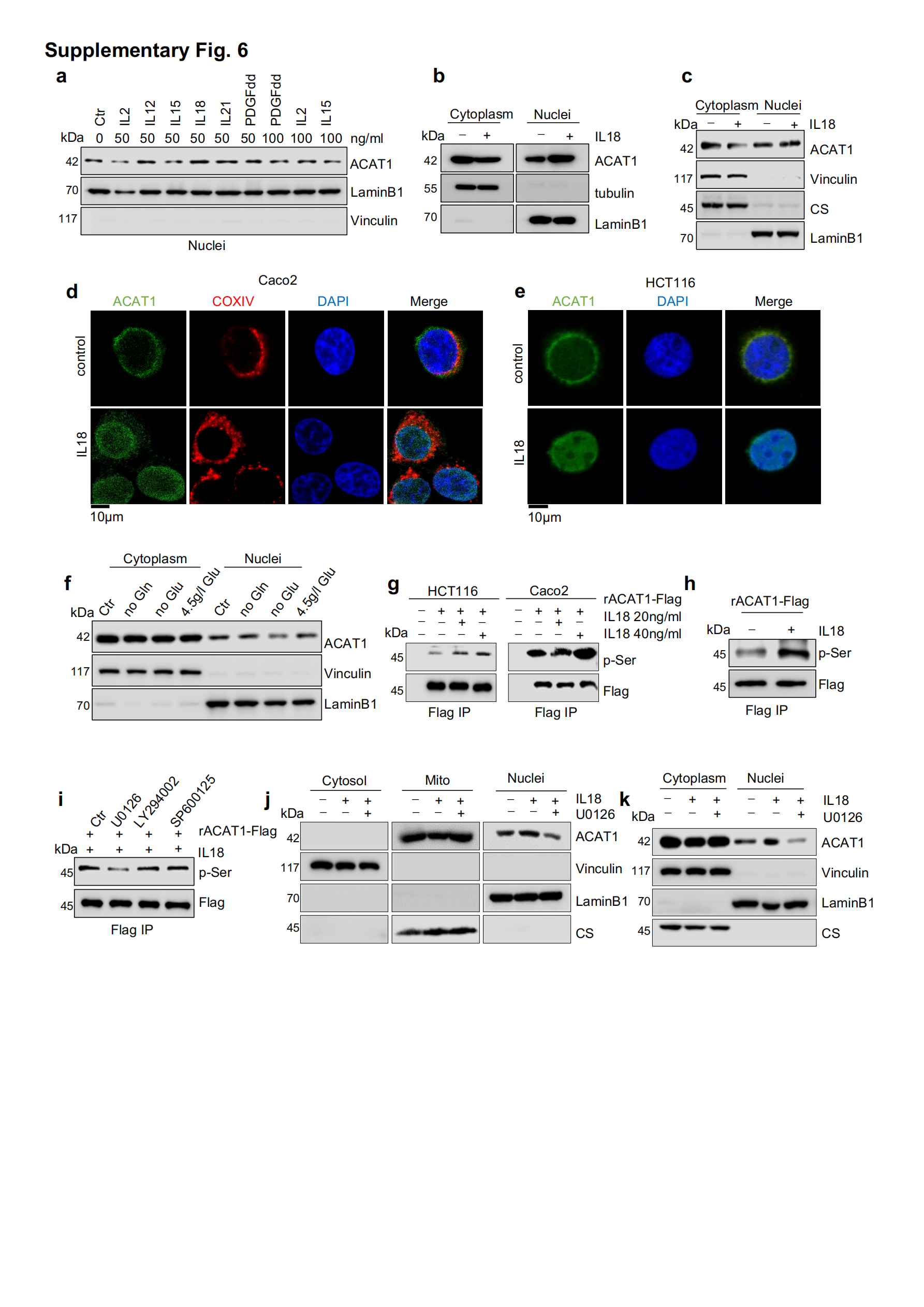

Supplement: Supplementary file 8 — Supplementary Fig.6 [file 41392_2025_2221_MOESM8_ESM.tif]

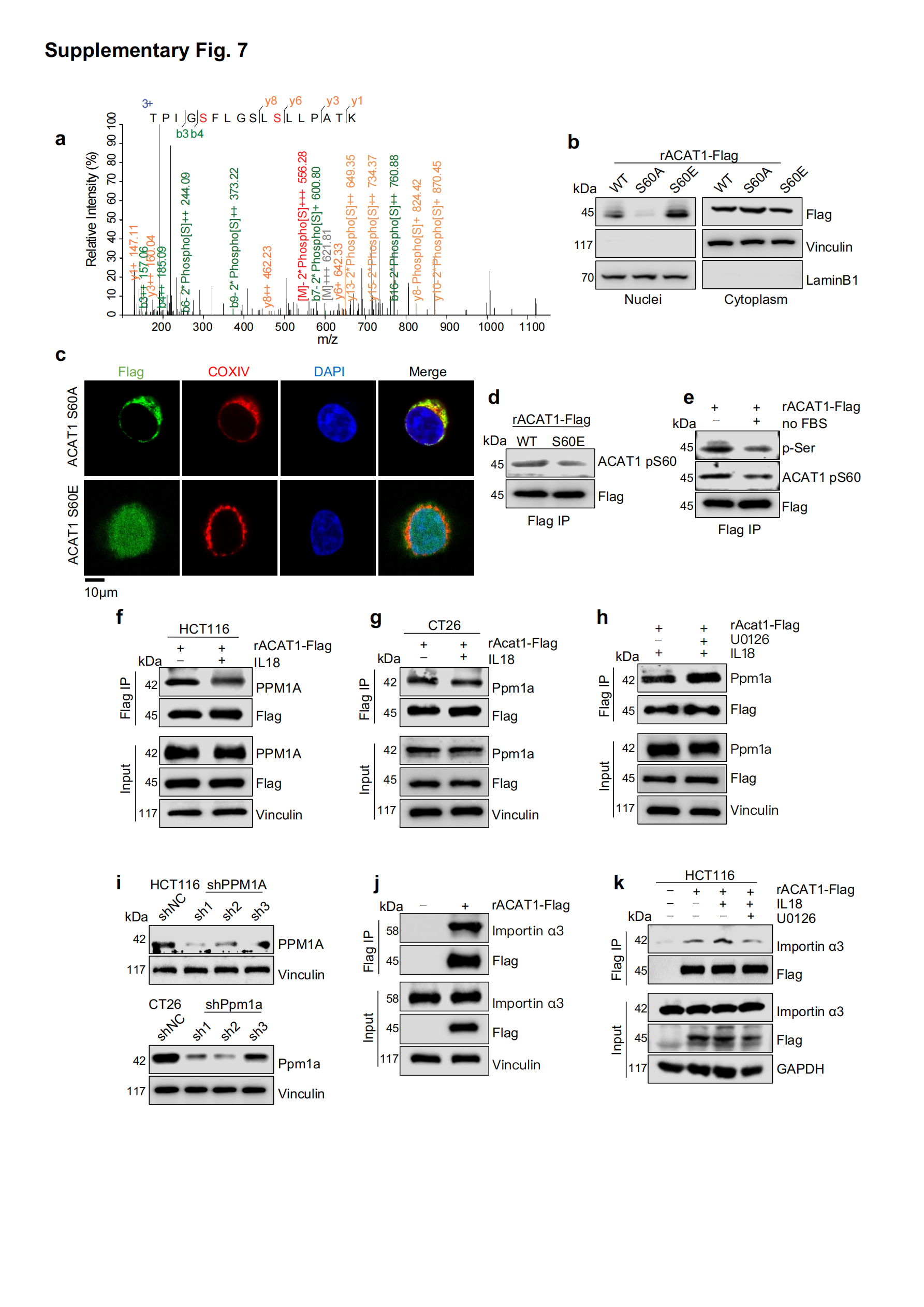

Supplement: Supplementary file 9 — Supplementary Fig.7 [file 41392_2025_2221_MOESM9_ESM.tif]

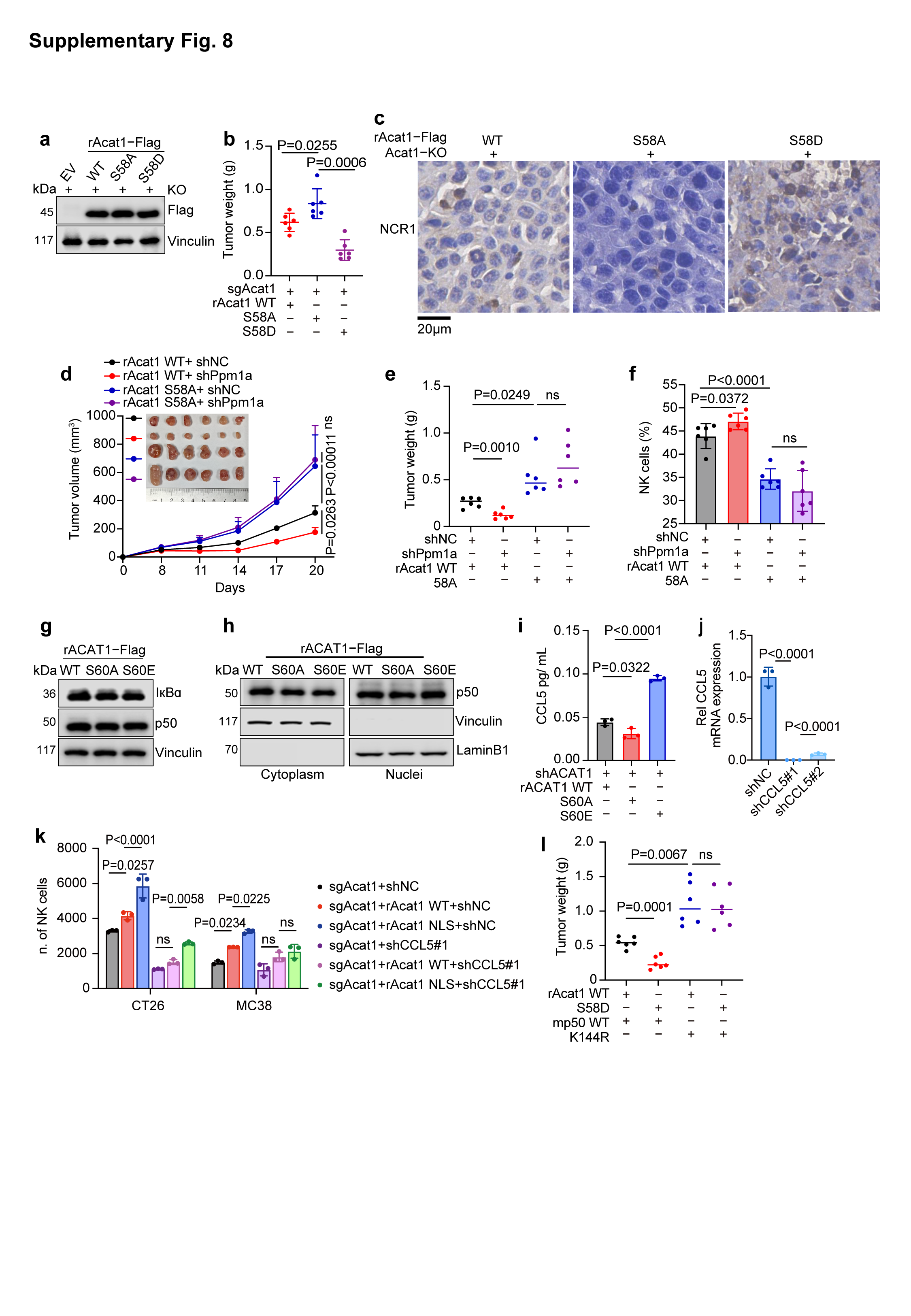

Supplement: Supplementary file 10 — Supplementary Fig.8 [file 41392_2025_2221_MOESM10_ESM.tif]
